# Identification of hub genes distinguishing subtypes in endometrial stromal sarcoma through comprehensive bioinformatics analysis

**DOI:** 10.1038/s41598-023-47668-7

**Published:** 2024-01-02

**Authors:** Ruiqi Zhang, Weilin Zhao, Xingyao Zhu, Yuhua Liu, Qi Ding, Caiyun Yang, Hong Zou

**Affiliations:** 1 Department of Pathology, The First Affiliated Hospital, Shihezi University School of Medicine, Xinjiang, 832002 China; 2 Department of Pathology, The Second Affiliated Hospital of Zhejiang University School of Medicine, Zhejiang, 310009 China; 3 Department of Pathology, Taihe Hospital, Hubei University of Medicine, Hubei, 442000 China

**Keywords:** Cancer, Genetics, Biomarkers, Medical research, Oncology

## Abstract

Diagnosing low-grade and high-grade endometrial stromal sarcoma (LG-ESS and HG-ESS) is a challenge. This study aimed to identify biomarkers. 22 ESS cases were analyzed using Illumina microarrays. Differentially expressed genes (DEGs) were identified via Limma. DEGs were analyzed with String and Cytoscape. Core genes were enriched with GO and KEGG, their pan-cancer implications and immune aspects were studied. 413 DEGs were found by exome sequencing, 2174 by GSE85383 microarray. 36 common genes were identified by Venn analysis, and 10 core genes including RBFOX1, PCDH7, FAT1 were selected. Core gene GO enrichment included cell adhesion, T cell proliferation, and KEGG focused on related pathways. Expression was evaluated across 34 cancers, identifying immune DEGs IGF1 and AVPR1A. Identifying the DEGs not only helps improve our understanding of LG-ESS, HG-ESS but also promises to be potential biomarkers for differential diagnosis between LG-ESS and HG-ESS and new therapeutic targets.

## Introduction

Endometrial stromal sarcoma (ESS), an infrequent mesometrial-origin tumor, exhibits marked malignancy levels^1,2^. The latest World Health Organization classification divides ESS into low-grade (LG-ESS) and high-grade (HG-ESS) categories based on severity^1^. Clinical studies underscore distinct treatment approaches and prognoses for these types, yet biomarkers for their differential diagnosis remain scarce. This study endeavors to identify potential biomarkers for distinguishing between HG-ESS and LG-ESS.

While the past decade's research methods for ESS have centered on molecular fusion gene markers crucial to diagnosis^3–6^, current literature predominantly comprises retrospective case reports. Studies, confined to immunohistochemistry and fusion genes, offer limited insights into genetic commonalities and disparities between LG-ESS and HG-ESS subtypes. Recent genomic innovations present opportunities to grasp comprehensive tumor tissue biology. Genome-wide single nucleotide polymorphism (SNP) analysis, assessing genomes and transcripts in small sample sets, can identify target genes effectively^7^. SNPs offer extensive human genome loci coverage, ease of high-throughput typing, and cost-efficient sequencing, catering to the rarity of ESS.

In this study, we employed exon microarray technology to sequence 22 wax block samples (15 LG-ESS and 7 HG-ESS cases). Combining these with GEO platform data facilitated initial differential expression gene (DEG) screening between LG-ESS and HG-ESS groups. STRING platform (Search Tool for Interacting Genes Retrieval) (Version 12.0) and Cytoscape software (Software platform for visualizing molecular interaction networks) (Version 3.7.2) analyses of core genes followed. Pan-cancer analysis, immune-related discussions, and functional enrichment shed light on LG-ESS and HG-ESS differential diagnosis, molecular pathways, and clinical treatment insights.

## Materials and methods

### Patient and tissue specimens

We included 22 tissue wax block samples (15 LG-ESS and 7 HG-ESS cases) from patients admitted at the Department of Pathology, First Affiliated Hospital, Shihezi University, School of Medicine, between 2012 and 2021.

### DNA extraction and SNP array

Tissue sections were obtained through laser capture microdissection (LCM) and processed for DNA extraction using the QIAamp DNA Micro Kit. DNA concentration was quantified using agarose gel electrophoresis and Nanodrop ND-2000. Genomic DNA (gDNA) concentrations up to 50 ng/L underwent whole genome amplification, followed by fragmentation, precipitation, and resuspension for hybridization onto the Illumina iScan Reader chip. After hybridization, the non-specifically bounding DNA was removed by washing, and the remaining specifically bound sites were extended by single bases, stained and scanned using Illumina iScan Reader. Campus Biotechnology Co., LTD supported the above sequencing process.

### Microarray data acquisition and standardization

From the Gene Expression Omnibus (GEO, https://www.ncbi.nlm.nih.gov/geo/) databases, 307 human endometrial stromal sarcoma datasets were retrieved using "Endometrial stromal sarcomas" as the keyword and "Homo sapiens" as the study subject, and the GSE85383 dataset (Last update date: August 31, 2017) was selected for analysis^8,9^. This dataset contained 9 LG-ESS and 4 HG-ESS samples, using Agilent GPL22303 SurePrint G3 Human GE 8 × 60 K Microarray. Differentially expressed genes (DEGs) were identified based on logFC values and adjusted p-values^10,11^ ( logFC (fold change) in upregulated ≥ 1.0 and downregulated genes ≤ minus 1.0, adjusted p-value < 0.05).

### Differential gene analysis

Raw data from iScan were analyzed using the GenomeStudio Genotyping module for normalization, clustering, and genotyping. Volcano and heat maps were utilized for visualization of DEGs.

### Venn analysis

Venn's analysis (http://bioinformatics.psb.ugent.be/webtools/Venn/), an online tool, was employed for meaningful DEGs intersection between exon microarray and GEO database results, yielding target genes.

### Differential gene core module construction and hub gene screening

The STRING database (Version 12.0) (https://cn.string-db.org/) was used for protein interaction analysis, generating a PPI network imported into Cytoscape software for visualization. In this study, the DEGs obtained from the screening between LG-ESS and HG-ESS have used the STRING database to get a PPI network, then have visualized by Cytoscape software. The top 10 ranked Hub genes were selected using cytoHubba and Maximal clique centrality (MCC) algorithm^12,13^.

### Functional enrichment analyses

DAVID platform (http://david-d.ncifcrf.gov/summary.jsp/) facilitated Gene Ontology (GO) and Kyoto Encyclopedia of Genes and Genomes (KEGG) enrichment analyses of DEGs, revealing enriched pathways and biological processes annotated by Hub genes.

### Hub genes' pan-cancer analysis

TIMER database (https://cistrome.shinyapps.io/timer/) was used to explore pan-cancer correlations of the ten core genes and their expression differences in 34 common human cancers.

### Immune-related differential gene analysis

The immunology database and analysis portal (ImmPort, https://www.immport.org/) database, encompassing almost all immune-related genes, was intersected with the differential genes obtained from what was accessed by Venn analysis to identify immune differential genes.

### Ethical considerations

Patient and family consent was obtained, adhering to ethical guidelines.

### Ethical approval and consent to participate

The present study was conducted in accordance with the Declaration of Helsinki for participant’s well-being and safety. We obtained 15 cases of LG-ESS and 7 cases of HG-ESS from patients at the First Affiliated Hospital of the School of Medicine, Shihezi University. Research involving human material, have been performed in accordance with the Declaration of Helsinki. Informed consent was obtained from each patient. The study protocol was approved by the Ethics Committee at the First Affiliated Hospital of the School of Medicine, Shihezi University.

## Results

### Patients and tissue specimens

The patient cohort, with an average age of 49.8 years (age range: 27–73), exhibited clinical symptoms such as irregular vaginal bleeding, abdominal pain, postmenopausal vaginal bleeding, and uterine fibroids. Diagnostic verification involved immunohistochemistry and consensus among at least three senior pathologists.

### Differential genes from SNP array

SNP microarray technology was utilized to explore gene-level disparities between HG-ESS and LG-ESS. After DNA extraction, qualified quality control, amplification, and hybridization, 413 differentially expressed genes (adjusted p value < 0.05) (Supplementary Table 1) were identified between LG-ESS and HG-ESS groups in this analysis^10,11^.

These results are available on the Human Genetic Resources Information Management Backup platform (https://ngdc.cncb.ac.cn/hgrip) and the China National Center for Bioinformation (CNCB, https://ngdc.cncb.ac.cn/omix/preview/ZXO0dVNm).

### Differential genes from GEO

**Figure 1:**
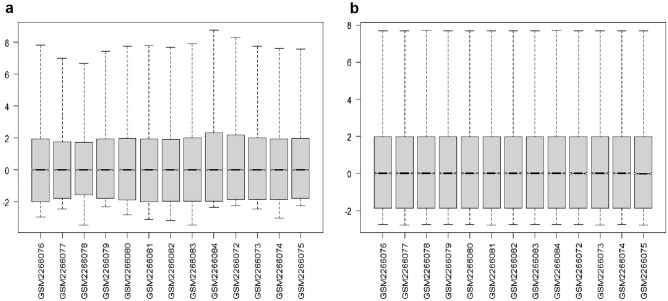
Data obtained from the GEO database were standardized using R language normalization. The raw data are shown in (**a**), while the normalized data are shown in (**b**).

**Figure 2:**
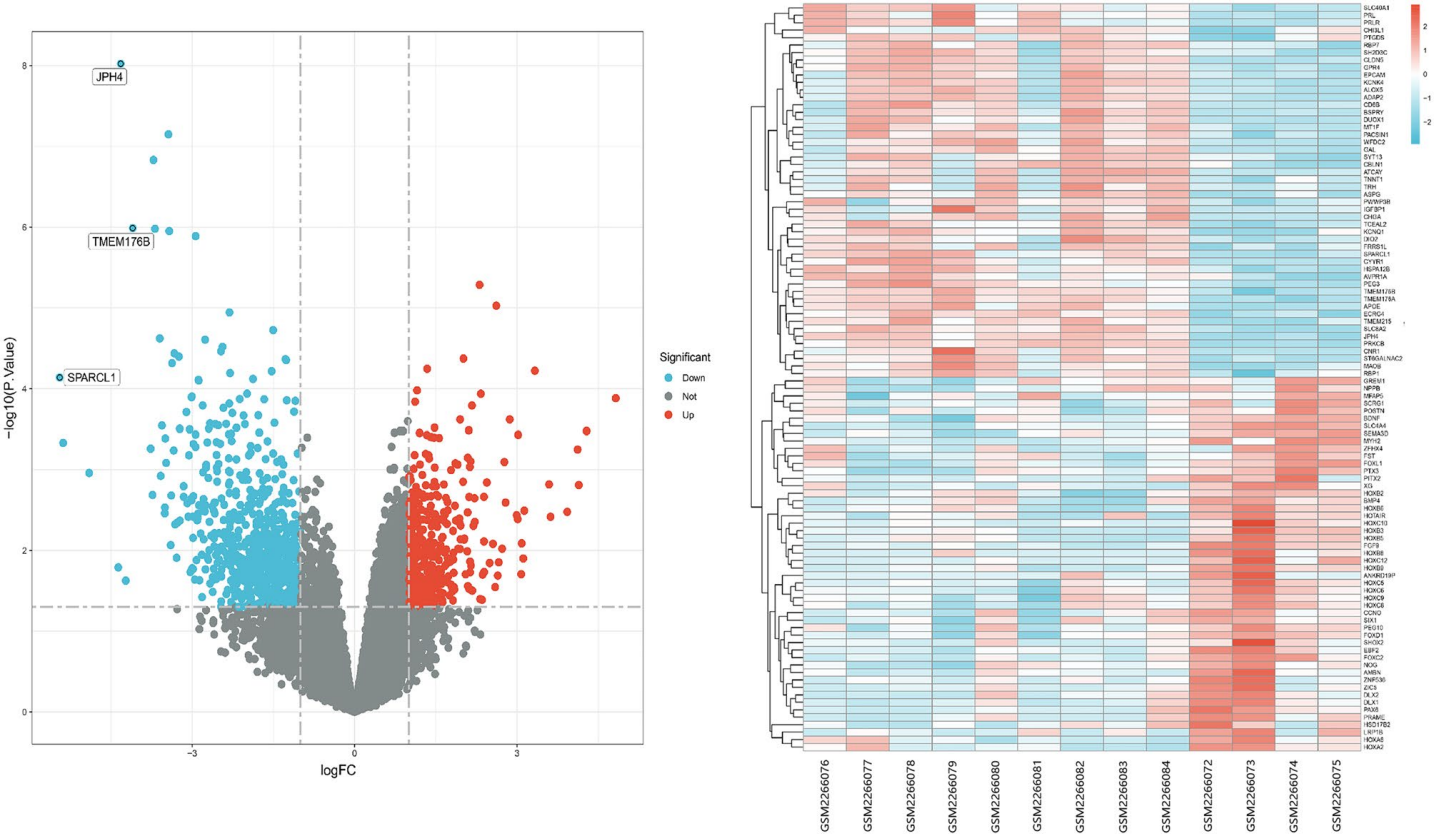
Analysis of differential gene expression in high and low-grade endometrial mesenchymal sarcomas within the GEO database. Red indicates high expression, while blue indicates low. Differentially expressed genes (DEGs) were identified based on logFC values and adjusted p-values (logFC (fold change) in upregulated ≥ 1.0 and downregulated genes ≤ minus 1.0, adjusted *p*-value < 0.05).

The expression profile data in GSE85383 obtained from the GEO database, whose original expression matrix has some differences (Fig. 1a), were standardized by R code to achieve comparability between each sample (Fig. 1b). 2174 differential genes were extracted (Supplementary Table 2), including 1225 up-regulated and 949 down-regulated genes in the HG-ESS group compared to the LG-ESS group with the visualization results shown in Fig. 2. DEGs were acquired by the measures of logFC (fold change) in upregulated ≥ 1.0 and downregulated genes ≤ minus 1.0, adjusted p value < 0.05^10,11^.

### Venn analysis

**Figure 3:**
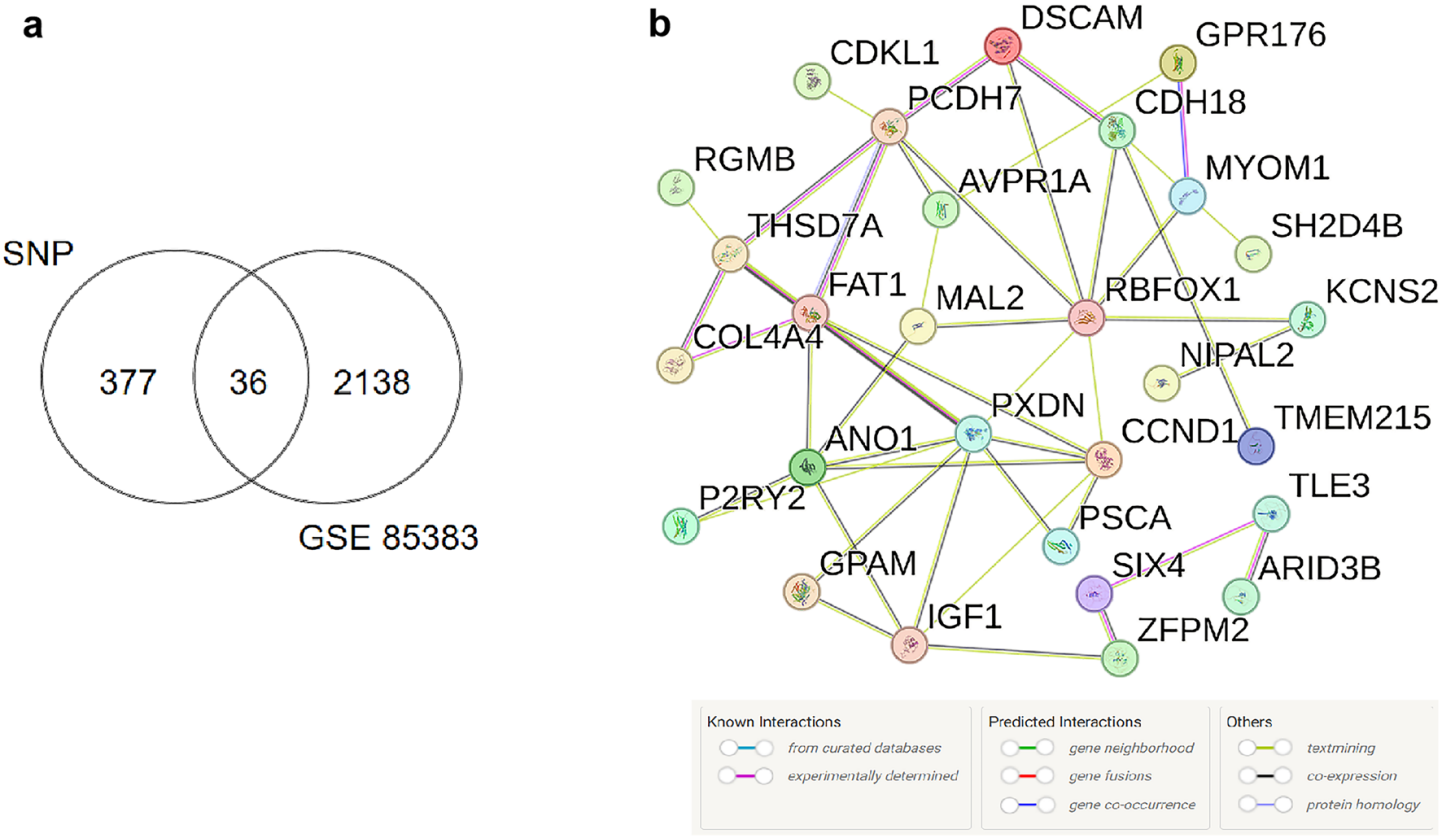
(**a**) Venn diagram depicting the overlap of differential genes identified through exon microarray sequencing and those from the GEO database. (**b**) Protein–Protein Interaction (PPI) network constructed using 36 co-expressed differential genes. The colored "balls" (or Nodes) represent proteins with direct effects; The lines between the "Nodes" represent the interaction between the two proteins, and the lines with different colors represent different interaction types.

Venn analysis revealed thirty-six shared genes from differential gene datasets obtained through exome chip sequencing and the GSE85383 database (Fig. 3a, Table 1).

**Table 1:** 36 differential genes shared by GSE85383 and whole genome exon microarray.

ID	logFC	AveExpr	t	Adjusted p-value	B	ID	logFC	AveExpr	t	Adjusted p-value	B
TMEM215	−3.59905	0.632208	−6.36569	2.38848E−05	2.767502	PCDH7	1.458674	2.262179	2.915264	0.011969	−2.76034
ANO1	−2.26008	−0.36114	−5.09656	0.000200207	0.923743	MYOM1	0.846907	0.168031	2.912129	0.012042	−2.76577
CDKL1	−1.31466	1.720534	−4.28986	0.000865168	−0.38442	MEX3B	1.024542	1.475148	2.744381	0.016621	−3.05485
AVPR1A	−3.09078	0.840141	−3.70822	0.002597161	−1.37841	NIPAL2	−1.55792	0.374762	−2.68386	0.018661	−3.15831
ZFPM2	1.720406	0.264728	3.600066	0.003195453	−1.56631	CASC8	1.036646	−2.00895	2.677233	0.018899	−3.16962
COL4A4	1.648617	1.922792	3.445591	0.004301089	−1.83561	ARID3B	0.927086	2.535178	2.643463	0.020157	−3.22709
PXDN	1.51259	5.104322	3.395512	0.004736993	−1.92307	MAL2	−2.46	0.243736	−2.62182	0.021006	−3.26382
KCNS2	−1.81381	−1.00928	−3.37522	0.004926081	−1.95853	GPAM	0.810188	1.4384	2.601997	0.021814	−3.29741
TLE3	1.262755	0.108287	3.315231	0.005530624	−2.06335	P2RY2	−1.69024	−0.11096	−2.5722	0.023085	−3.34777
CDH18	1.108811	−2.06506	3.216524	0.006692006	−2.23583	RBFOX1	−1.31976	−0.93157	−2.55491	0.023855	−3.3769
ARSG	−1.50268	1.212459	−3.19385	0.006991595	−2.27543	IGF1	−2.66995	1.562153	−2.5166	0.025652	−3.44128
MPP6	1.031841	0.867851	3.183601	0.007131411	−2.29333	DOCK5	0.88201	1.34373	2.364707	0.034141	−3.69352
RGMB	1.1115	0.890364	3.148575	0.007630613	−2.35448	LARS2	0.424636	2.53295	2.255162	0.041863	−3.87197
DSCAM	0.77037	−1.20364	3.148086	0.007637821	−2.35533	SH2D4B	0.591028	−2.01465	2.254468	0.041917	−3.87309
PSCA	1.260531	−0.25743	3.137808	0.007790965	−2.37327	CAPSL	0.664232	−0.74753	2.184743	0.047669	−3.98491
TARBP1	1.032928	1.147906	3.098376	0.008407489	−2.44204	GPR176	0.652708	2.200711	2.162982	0.049611	−4.01951
FAT1	1.58717	3.744343	3.088203	0.008574287	−2.45978	THSD7A	−2.14263	0.145424	−2.15951	0.049928	−4.02501
CCND1	1.421524	3.030863	3.045682	0.009307872	−2.53385	SIX4	1.103884	0.301445	2.159043	0.04997	−4.02575

### Differential gene core module construction and hub gene screening

**Figure 4:**
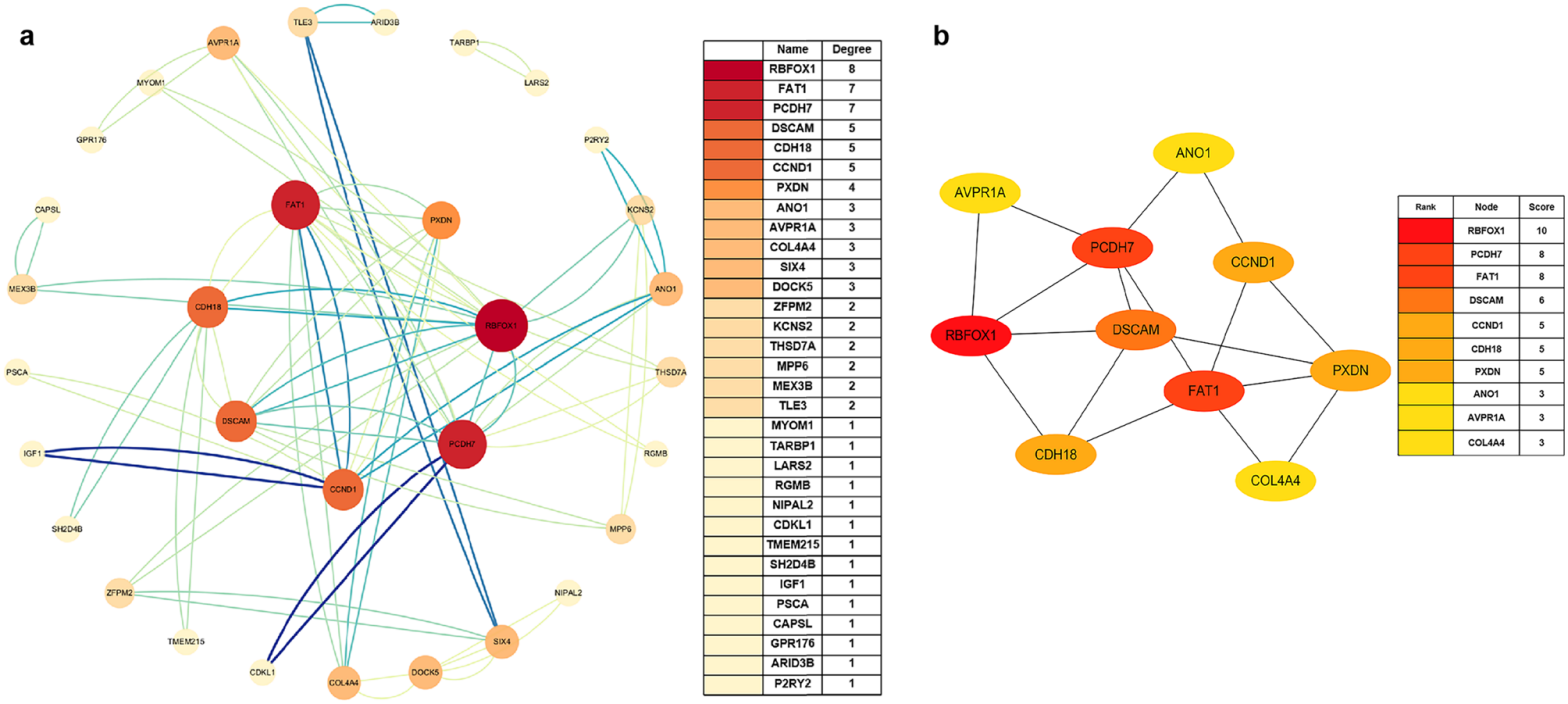
(**a**) Module of hub genes constructed using the 36 co-expressed differential genes. The value of the gene degree is represented by the size and color of the node. (**b**) Top ten core genes displayed in a protein interaction network connectivity diagram. The value of the gene rank is represented by the color of the node in the Maximal clique centrality (MCC) algorithm.

PPI network analysis via the STRING database generated protein interaction insights for the 36 shared differential genes (Fig. 3b). Then the PPI-related results were imported into the cytoHobba plugin in Cytoscape. The top 10 ranked core genes in the core module were screened as RBFOX1, PCDH7, FAT1, DSCAM, CCND1, CDH18, PXDN, ANO1, AVPR1A, COL4A4, where genes PCDH7, FAT1, DSCAM, CCND1, CDH18, PXDN, COL4A4 were up-regulated in the high-level group compared to the low-level group; genes RBFOX1, ANO1, AVPR1A were decreased in the high-level group compared to the low-level group. The above results were plotted in the protein interaction network Fig. 4a,b.

### Functional enrichment analyses of hub genes

GO analysis results shown in Table 2 were obtained by enrichment of the above 10 core genes, which mainly included biological processes related to cell adhesion, T cell proliferation, protein secretion, calcium ion binding, and transcriptional corepressor activity. KEGG pathway enrichment focused on adhesion-related PI3K-Akt and p53 signaling pathways(Table 3).

**Table 2:** Differential gene GO enrichment results of high and low grade endometrial stromal sarcomas.

Category	Term	Genes	Adjusted p-value
Biological Process	Homophilic cell adhesion via plasma membrane adhesion molecules	DSCAM, PCDH7, FAT1, CDH18	0.002381
Biological Process	Cell adhesion	DSCAM, PCDH7, PXDN, FAT1, RGMB	0.010451
Biological Process	Response to corticosterone	CCND1, AVPR1A	0.030717
Biological Process	Positive regulation of activated T cell proliferation	GPAM, IGF1	0.04424
Biological Process	Positive regulation of protein secretion	MYOM1, IGF1	0.077976
Biological Process	Phospholipase C-activating G-protein coupled receptor signaling pathway	ANO1, P2RY2	0.096532
Cellular Component	Integral component of plasma membrane	ANO1, GPR176, DSCAM, P2RY2, PCDH7, FAT1, AVPR1A	0.031435
Cellular Component	Plasma membrane	DOCK5, PSCA, DSCAM, PCDH7 AVPR1A, RGMB, ANO1, GPR176, GPAM KCNS2, P2RY2, FAT1, THSD7A, CDH18	0.044807
Cellular Component	Membrane raft	ANO1, MAL2, RGMB	0.061657
Molecular Function	Calcium ion binding	MEX3B, CAPSL, PCDH7, FAT1, CDH18	0.033854
Molecular Function	Transcription corepressor activity	TLE3, CCND1, ZFPM2	0.041216

**Table 3:** KEGG enrichment analysis of differential genes between high and low grade endometrial stromal sarcomas.

Pathway	Genes	adjusted p-value
Focal adhesion	CCND1, COL4A4, IGF1	0.028713
PI3K-Akt signaling pathway	CCND1, COL4A4, IGF1	0.079762
Melanoma	CCND1, IGF1	0.092986
p53 signaling pathway	CCND1, IGF1	0.09422
Glioma	CCND1, IGF1	0.096684

### Hub genes' pan-cancer analysis

**Figure 5:**
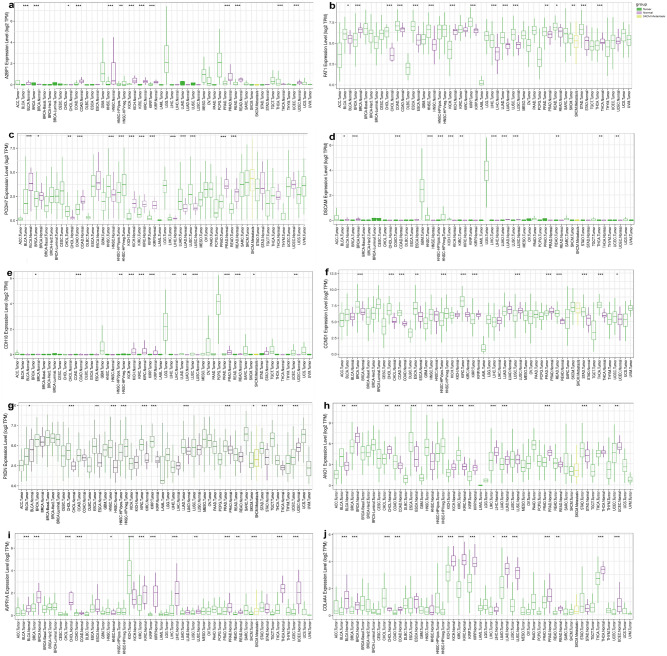
Expression profiles of the 10 hub genes across 34 common human cancers analyzed in a pan-cancer context. Green represents tumor samples, purle represents normal control samples and yellow represents SKCM Metastasis samples. (*p value < 0.05, **p value < 0.01, ***p value < 0.001).

Pan-cancer analysis across 34 common human cancers unveiled distinct expression patterns for the ten core genes, as shown in Fig. 5. In this study, the expression of the RBFOX1 gene (or A2BP1), which had decreased expression in the high-grade group compared with the low-grade group, differed in 13 cancer types (Fig. 5a). In four cancers, including breast cancer (BRCA), the expression levels of tumor tissues were higher than normal tissues; While in other eight cancers, such as colon cancer (COAD), head and neck squamous cell carcinoma (HNSC), the expression levels of tumor tissues were lower than normal tissues. The expression of the FAT1 gene increased in the high-grade group compared with the low-grade group and was differently expressed in 16 cancer species (Fig. 5b). In 12 kinds of cancers, such as Bladder urothelial carcinoma (BLCA), the expression level of the tumor tissue was higher than that of the normal tissue; In BRCA and Kidney Chromophobe (KICH), the expression level in tumor tissues was lower than that in normal tissues. The expression of the PCDH7 gene, which was higher in HG-ESS than LG-ESS, was different in 14 cancer species (Fig. 5c). In four carcinomas within cholangiocarcinoma (CHOL), their expression levels were higher in tumor tissues than in normal tissues. In BLCA and BRCA, etc., their expression levels in tumor tissues were lower. The DSCAM gene with increased expression occurred in the high-grade group in this study. It differed in 13 cancer types (Fig. 5d): in 11 cancers such as HNSC and Uterine Corpus Endometrial Carcinoma (UCEC), their expression levels in tumor tissues were higher; only in BRCA cancer, their expression levels in tumor tissues were lower than normal tissues. In this study, the expression of the CDH18 gene, which was increased in the HG-ESS, differed in 13 cancer types (Fig. 5e). In Lung squamous cell carcinoma (LUSC), the tumor tissues with higher expression of it. In 5 cancers, including COAD and Kidney clear cell carcinoma (KIRC), they were lower. The CCND1 gene with increased expression in the HG-ESS differed in 13 cancer types (Fig. 5f). In 9 cancers as Rectal Cancer (READ), the tumor’s expression was higher than the normal’s; the lower face could be seen in the tumor part rather than the standard part in Kidney Papillary Cell Carcinoma (KIRP) and LUSC. Mentioning the PXDN gene’s expression (Fig. 5g), which with an increased level in the HG-ESS, was unevenly expressed in 13 cancers. The expression levels in tumors higher than normal’s could be found in CHOL and HNSC carcinomas, while the expression levels in tumors lower than normal’s could be found in BLCA and UCEC. The expression of the ANO1 gene, which was lower in HG-ESS than LG-ESS, was different in 16 cancer species (Fig. 5h). In six carcinomas within Esophageal Cancer (ESCA), their expression levels were higher in tumor tissues than in normal tissues, while in CHOL, KICH, etc., their expression levels in tumor tissues were lower. The AVPR1A gene with decreased expression in the HG-ESS differed in 15 cancer types (Fig. 5i). In KICH and HNSC, their tumor’s expression was higher than the normal’s, while the lower expression could be seen in the tumor part rather than the normal part in BLCA and Prostate Cancer (PRAD), etc. The term of the COL4A4 gene, which was increased in the high-grade group compared with the low-grade group, differed in 15 cancer types (Fig. 5j). In CHOL cancer, it overexpressed in tumor tissues more than the normal part, while in 13 cancers, including BRCA and KICH, its expression level in tumor tissues was lower than normal.

### Immune-related differential gene analysis

**Figure 6:**
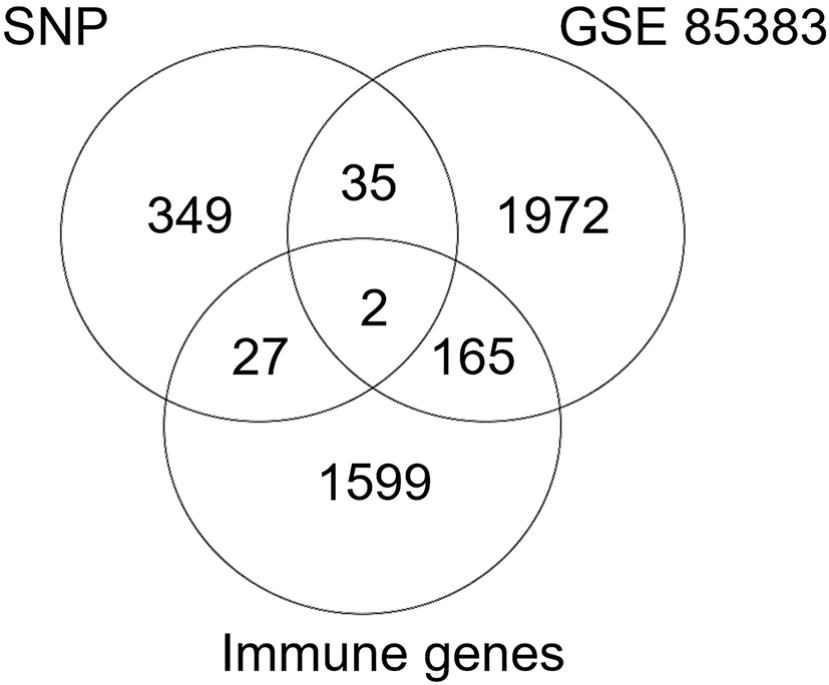
Venn diagram illustrating the overlap between immune-related genes and differentially expressed genes.

All immune-related genes 1793 were downloaded from the ImmPort official website. Through comparison with ImmPort's immune-related genes, two genes, IGF1 and AVPR1A, were identified with down-regulated expressions in the high-grade group compared to the low-grade group (Fig. 6).

## Discussion

ESS is a rare malignant neoplasm of the female genital tract, categorized into low-grade (LG-ESS) and high-grade (HG-ESS), presents distinctive clinical and prognostic variations^14,15^. However, distinguishing between these subtypes using conventional methods remains challenging. Advances in sequencing technology and data sharing platforms provide an opportunity to comprehend disease genotypes as a foundation for molecular targeted therapy^7^. This study identified genes with differential expression between HG-ESS and LG-ESS through sequencing and bioinformatics analysis, focusing on ten meaningful candidates (RBFOX1, PCDH7, FAT1, DSCAM, CCND1, CDH18, PXDN, ANO1, AVPR1A, COL4A4). Several of these genes, including CCND1 and ANO1, have been associated with ESS previously, reinforcing the reliability of this analysis. Rekhi B et al. suggested that positive expression of Cyclin D1 was positively associated with more aggressive ESSs by reporting a particular ESS^16^; A study by Lee CH and Subbaraya S et al. suggested that positive expression of Cyclin D1 and ANO1 could be used as diagnostic immunomarkers for HG-ESS associated with YWHAE gene rearrangement^17,18^; The unexplored effects of RBFOX1, PCDH7, FAT1, DSCAM, CDH18, PXDN, AVPR1A, and COL4A4 in ESS warrant further investigation.

RBFOX1, the RNA binding fox-1 homolog 1, previously associated with developmental coordination disorder and spinal cerebellar ataxia, has also been linked to tumors like gastric and colon cancers now. For example, in the study of colorectal cancer in British Bangladeshis, RBFOX1 deletion was associated with high prevalence, early onset, and frequent mucous tissue types of colorectal cancer^19^. In the study of Malignant Mesothelioma (MMt), a pure deletion mutation in RBFOX1/A2BP1 was also identified for the first time, and the possibility was raised that this gene could be deemed as a new suppressor in MMt^20^. It has also been documented that RBFOXI may be involved in the pathogenesis of acute kidney injury by inhibiting the inflammatory response and oxidative stress and reducing the apoptosis of HK-2 cells induced by the hypoxic environment^21^. In this paper, the relationship between the gene RBFOXI and ESS disease was discussed for the first time. Considering the sequencing of the RBFOXI gene in this study (the RBFOXI gene showed a decreased expression in the HG-ESS group compared with the LG-ESS group) and the results of the pan-cancer analysis in the TIMER database, it can be proposed that RBFOXI can be a differential gene with tumor suppressive effect between the HG-ESS and LG-ESS groups.

FAT atypical cadherin 1 (FAT1), a tumor suppressor through WNT/β-catenin, Hippo, and MAPK/ERK pathways, influences tumor progression and affects therapy response in various cancers. The FAT1 gene deficiency in breast cancer affects resistance to CDk4/6 inhibitor therapy^22^; FAT1 mutations are associated with poor survival in head and neck squamous cell carcinoma (HNSCC)^23,24^; Some studies suggest that FAT1 may be an immune response regulator involved in different inflammatory processes, as has been argued in gliomas and T-cell lymphomas^25,26^. Some researchers have also obtained different results: Kim et al. suggested that human papillomavirus (HPV)-negative HNSCC patients with FAT1 mutation showed a better prognosis^27^. In melanoma and NSCLC, FAT1 can inhibit the tumor initiation ability of NSCLC cells by activating the Hippo signaling pathway, resulting in a significantly better survival outcome in patients with FAT1 mutations than the wild type and correlating with better immunogenicity and ICI efficacy. Thus, FAT1 can be recommended as a biomarker for immunotherapy in patients with melanoma and NSCLC^24^. A recent study showed that immunotherapy is increasingly used in sarcomas, not limited to patients with solid tumors^28^. Based on these results, the present study suggests that the differential expression of FAT1 between HG-ESS and LG-ESS may have important biological significance. The differential expression of FAT1 between HG-ESS and LG-ESS could impact clinical outcomes and immunotherapy.

PCDH7, a cell–cell adhesion regulator, emerges as a prognostic factor across cancers. Its roles vary. PCDH7 was defined as a risk gene, and its high expression suppressed survival in lung cancer patients in a study by Chen Y et al.^29^. PCDH7 provides a potential therapeutic strategy in colon cancer: it can inhibit the MEK1/2/ERK/c-Fos axis by knocking down PCDH7, induce neurogenesis and autophagy, and enhance the effect of colon cancer cells on chemotherapy^30^. Since androgen receptors can target PCDH7, discussing the relationship between the degree of PCDH7 methylation and the growth, invasion, and apoptosis of AIPC cells in non-androgen-dependent prostate cancer (AIPC) provides a new idea for the treatment of AIPC^31^. However, in gastric cancer PRMT6-KO-GC cells, knockdown of tumor suppressor gene PCDH7 promoted cell migration and invasion^32^. Meanwhile, in cervical cancer, up-regulation of PCDH7 can significantly inhibit the proliferation, migration, and invasion of cancer cells, and PCDH7 is positively correlated with the survival rate of patients^33^. In summary, PCDH7 has been considered an independent prognostic factor in various cancers. Further experimental validation is needed to determine whether it can provide a new target and therapeutic idea for the treatment of HG-ESS through the hints in this paper.

Enrichment analyses unveiled pathways like cell adhesion, PI3K-Akt, and p53 signaling associated with tumor progression. Studies have shown that changes in cell surface receptor expression often occur in malignant tumors; The activation of adhesion signaling pathways plays an essential role in cell differentiation, development, proliferation, and apoptosis and influences tumor progression by participating in tumor invasion, motility, and metastasis processes. As one of the classical signal transduction pathways, the abnormal activation of the PI3K-Akt signaling pathway and a variety of downstream effector molecules is closely related to the biological characteristics of the malignant proliferation of tumor cells^34^, which is consistent with the information we obtained in the clinic that tumors in the high-grade group are larger, more mitotically active, more commonly necrotic, more extensively infiltrated, and infiltration can involve the myometrium, lymph and blood vessels^2^, and the above enrichment analysis further explains the high malignancy and poor prognostic outcome in the high-grade group from the genetic level. Moreover, immune-related genes were implicated, indicating potential for immunotherapy. They are not only involved in the biological progression of positive regulation of activated T cell proliferation, but we also obtained three immune-related genes, IGF1, AVPR1A, and FAT1, by bioinformatics analysis. With the above hints, the idea that patients may benefit from immunotherapy is more testable.

According to the latest guidelines, the treatment of ESS patients is still very limited by the lack of specific targeted therapies or immunotherapy. However, some pan-tumor targets such as neurotrophic receptor tyrosine kinase (NTRK) gene fusions, microsatellite instability (MSI), and tumor mutation burden (TMB) have been tried in clinical work as adjuvant therapy. For example, some studies have suggested that immunotherapy, such as pembrolizumab, may be an option for primary or relapsed patients with TMB ≥ 10 who are surgically unresectable or have multiple metastases throughout the body when more good treatment options are not available^21^. The discussion of FAT1, PCDH7, and other essential genes in this paper may prompt questions about these genes as therapeutic targets and independent prognostic indicators of ESS. Could these key genes be therapeutic targets and independent prognostic factors that distinguish HG-ESS from LG-ESS, and could they guide immunotherapy? Also, considering the activated PI3K-AKT pathway, could inhibitors of this pathway offer new therapeutic prospects? In the clinical context, genetic testing could guide targeted and immunotherapeutic approaches for patients with limited treatment options. In conclusion, this study initiates the exploration of candidate genes, offering new insights into ESS treatment. While limitations exist due to sample size and methods, further validation and experimental expansion are planned to advance these findings.

## Conclusion

In summary, our study, utilizing SNP sequencing and the GEO database, employed bioinformatics methods to analyze core genes, including RBFOX1, revealing insights into the molecular underpinnings of HG-ESS development. These core genes could potentially serve as biomarkers and therapeutic targets for HG-ESS.

### Supplementary Information

Supplementary Information.

## Data Availability

In the analysis process of this paper, after determining the parameters of each analysis step, we carried out at least three repeated experiments, so the analysis results of this study were obtained by more than three parallel experiments. The part of data that support the findings of this study are available from the online database (GEO, https://www.ncbi.nlm.nih.gov/geo/). The dataset analysed in this paper is GSE85383 dataset (Last update date: August 31, 2017). Datas, analyzed by SNP microarray technology, are available on the Human Genetic Resources Information Management Backup platform (https://ngdc.cncb.ac.cn/hgrip) and the China National Center for Bioinformation (CNCB, https://ngdc.cncb.ac.cn/omix/preview/ZXO0dVNm). SNP sequencing data saved in https://ngdc.cncb.ac.cn/omix/preview/ZXO0dVNm website, serial Numbers for OMIX004254-01. Other relevant data supporting the key findings of this study are available within the article and its Supplementary Material. It also can be obtained from the corresponding author upon reasonable request.

## Abbreviations

ESS: Endometrial stromal sarcoma
LG-ESS: Low-grade endometrial stromal sarcoma
HG-ESS: High-grade endometrial stromal sarcoma
DEGs: Differentially expressed genes
PPI: Protein–protein interaction network
GO: Gene ontology
KEGG: Kyoto encyclopedia of genes and genomes
BP: Biological process
CC: Cellular component
MF: Molecular function
SNP: Single nucleotide polymorphism
GEO: Gene expression omnibus
TIMER: Tumor immune estimation resource
IHC: Immunohistochemistry
