# Supplementary material for: Identification of hub genes distinguishing subtypes in endometrial stromal sarcoma through comprehensive bioinformatics analysis
Source: Sci Rep. 2024 Jan 2;14:37. doi: 10.1038/s41598-023-47668-7 (PMC10761685; doi:10.1038/s41598-023-47668-7)
Supplement: Supplementary file 1 — Supplementary Information. [file 41598_2023_47668_MOESM1_ESM.docx]

**Supplementary table 1.The differential genes between high grade and low grade endometrial stromal sarcomas were screened by exon microarray.**

| Gene | CHR | P | BP | Gene | CHR | P | BP |
| --- | --- | --- | --- | --- | --- | --- | --- |
| TMPRSS3 | 21 | 0.001051 | 43803167 | SHANK2 | 11 | 0.03571 | 70731148 |
| DNAAF1 | 16 | 0.001374 | 84203612 | SPACA7 | 13 | 0.03571 | 113097722 |
| GKN2 | 2 | 0.001399 | 69173570 | AGBL1 | 15 | 0.03571 | 87755594 |
| C3orf20 | 3 | 0.001508 | 14724345 | DNAH3 | 16 | 0.03571 | 20976360 |
| ESYT3 | 3 | 0.001508 | 138191232 | HEXDC | 17 | 0.03571 | 80391684 |
| LOC100507065 | 12 | 0.02564 | 66117558 | KIF19 | 17 | 0.03571 | 72351406 |
| ATG4C | 1 | 0.002879 | 63493164 | TOM1L1 | 17 | 0.03571 | 53001456 |
| PSCA | 8 | 0.002879 | 143761931 | ATP8B3 | 19 | 0.03571 | 1784944 |
| MPHOSPH10 | 2 | 0.002997 | 71366957 | ZNF135 | 19 | 0.03571 | 58572979 |
| LINC01268 | 6 | 0.002997 | 114196015 | CRNN | 1 | 0.03571 | 152454591 |
| PTCH1 | 9 | 0.002997 | 98482261 | TSPAN5 | 4 | 0.03571 | 99478756 |
| OR5B3 | 11 | 0.004022 | 58170342 | ADAMTS3 | 4 | 0.03571 | 73414286 |
| LINC01031 | 1 | 0.004022 | 193921548 | NPR3 | 5 | 0.03571 | 32786389 |
| DNAH11 | 7 | 0.004022 | 21607352 | LOC101927835 | 5 | 0.03571 | 163889280 |
| MIR7641-2 | 8 | 0.004022 | 93201255 | PDE1C | 7 | 0.03571 | 32273107 |
| CCNA1 | 13 | 0.004662 | 37178919 | COG5 | 7 | 0.03571 | 106938420 |
| CD180 | 5 | 0.004662 | 66538400 | FBXO32 | 8 | 0.03571 | 124526607 |
| ITIH2 | 10 | 0.004762 | 7755623 | ADRA2A | 10 | 0.03595 | 113042093 |
| IL1RAP | 3 | 0.004762 | 190480177 | NOS1 | 12 | 0.03595 | 117693817 |
| CARS | 11 | 0.004995 | 3036324 | LINC00520 | 14 | 0.03595 | 56295580 |
| NDC80 | 18 | 0.006536 | 2631687 | NUTM1 | 15 | 0.03595 | 34648935 |
| PAX1 | 20 | 0.006536 | 21741526 | KDM6B | 17 | 0.03595 | 7750436 |
| LRP12 | 8 | 0.006536 | 106142333 | ARL16 | 17 | 0.03595 | 79650828 |
| P2RY2 | 11 | 0.006993 | 72946020 | ZZEF1 | 17 | 0.03595 | 3951946 |
| Gene | CHR | P | BP | Gene | CHR | P | BP |
| SKP1P2 | 12 | 0.006993 | 17653834 | HGS | 17 | 0.03595 | 79664426 |
| SACS | 13 | 0.006993 | 23953924 | GSDMB | 17 | 0.03595 | 38069949 |
| GPC5 | 13 | 0.006993 | 93331886 | MYOM1 | 18 | 0.03595 | 3126811 |
| CSMD2 | 1 | 0.006993 | 34238283 | HNF4A-AS1 | 20 | 0.03595 | 43018260 |
| NLGN1 | 3 | 0.006993 | 173469241 | FGF12 | 3 | 0.03595 | 192312270 |
| ANKRD31 | 5 | 0.006993 | 74442920 | SORCS2 | 4 | 0.03595 | 7219933 |
| MROH5 | 8 | 0.04299 | 142488837 | MIR4457 | 5 | 0.03595 | 1308552 |
| NPTN | 15 | 0.007617 | 73929859 | HLA-DOA | 6 | 0.03595 | 33007752 |
| MYO15A | 17 | 0.008242 | 18023897 | CDKAL1 | 6 | 0.03595 | 21384613 |
| SLC4A1 | 17 | 0.008242 | 42338945 | THSD7A | 7 | 0.03595 | 11704583 |
| FXYD5 | 19 | 0.008242 | 35660508 | RGS22 | 8 | 0.03595 | 101011612 |
| COBL | 7 | 0.008242 | 51527696 | SORBS1 | 10 | 0.04056 | 97348734 |
| DOCK5 | 8 | 0.008242 | 25242601 | DHTKD1 | 10 | 0.04056 | 12143105 |
| DLC1 | 8 | 0.008242 | 13356802 | C11orf74 | 11 | 0.04056 | 37700681 |
| SCAF11 | 12 | 0.009657 | 46321514 | TSPAN9 | 12 | 0.04056 | 3250797 |
| LINC00593 | 15 | 0.009657 | 70208373 | AVPR1A | 12 | 0.04056 | 63789836 |
| KCNJ2 | 17 | 0.009657 | 68900605 | FGF14-IT1 | 13 | 0.04056 | 102959556 |
| SETBP1 | 18 | 0.009657 | 42481985 | ATP5S | 14 | 0.04056 | 50788213 |
| COA6 | 1 | 0.009657 | 234524045 | FUT8 | 14 | 0.04056 | 66699865 |
| MGAT4A | 2 | 0.009657 | 99279354 | JMJD7-PLA2G4B | 15 | 0.04056 | 42134097 |
| LOC102723828 | 4 | 0.009657 | 32962030 | TMC3-AS1 | 15 | 0.04056 | 81847279 |
| LINC00680-GUSBP4 | 6 | 0.009657 | 58344955 | SNTB2 | 16 | 0.04056 | 69224615 |
| SBSPON | 8 | 0.009657 | 73982161 | WRAP53 | 17 | 0.04056 | 7606722 |
| TRPA1 | 8 | 0.009657 | 73106916 | RHBDL3 | 17 | 0.04056 | 30625205 |
| SGSH | 17 | 0.0101 | 78184393 | ENO3 | 17 | 0.04056 | 4856376 |
| Gene | CHR | P | BP | Gene | CHR | P | BP |
| MICAL3 | 22 | 0.0101 | 18337880 | CHMP6 | 17 | 0.04056 | 78988362 |
| EPHB1 | 3 | 0.0101 | 135288025 | STAT3 | 17 | 0.04056 | 40514201 |
| CMTM7 | 3 | 0.0101 | 32439785 | MALT1 | 18 | 0.04056 | 56362913 |
| HCG9 | 6 | 0.0101 | 29963065 | EPS8L3 | 1 | 0.04056 | 110346355 |
| UNC13B | 9 | 0.0101 | 35289544 | TRMT6 | 20 | 0.04056 | 5923204 |
| OVOL1 | 11 | 0.01084 | 65579670 | C21orf91 | 21 | 0.04056 | 19169218 |
| LRIT3 | 4 | 0.01084 | 110773108 | GCAT | 22 | 0.04056 | 38205989 |
| CHKA | 11 | 0.01136 | 67867548 | LOC400940 | 2 | 0.04056 | 6500101 |
| CADM1 | 11 | 0.01136 | 115515326 | ARPP21 | 3 | 0.04056 | 36377812 |
| FAT1 | 4 | 0.01136 | 187678866 | ZNF385D-AS2 | 3 | 0.04056 | 22350158 |
| LRRC23 | 12 | 0.01282 | 7023123 | ATP10D | 4 | 0.04056 | 47578971 |
| LINC00544 | 13 | 0.01282 | 30670985 | CWC27 | 5 | 0.04056 | 64099769 |
| C17orf51 | 17 | 0.01282 | 21717727 | ANKH | 5 | 0.04056 | 14989346 |
| PNPLA3 | 22 | 0.01282 | 44332570 | CCDC152 | 5 | 0.04056 | 42782492 |
| CAND2 | 3 | 0.01282 | 12858028 | LINC01340 | 5 | 0.04056 | 97469921 |
| C7 | 5 | 0.01282 | 40955561 | LOC100132735 | 6 | 0.04056 | 140197079 |
| BRPF3 | 6 | 0.01282 | 36198577 | HLA-G | 6 | 0.04056 | 29794317 |
| AKNA | 9 | 0.01282 | 117103973 | IMPG1 | 6 | 0.04056 | 77566201 |
| FRA10AC1 | 10 | 0.01499 | 95454681 | ASPH | 8 | 0.04056 | 62603795 |
| PNLIPRP3 | 10 | 0.01499 | 118228763 | LRRC37A5P | 9 | 0.04056 | 114382444 |
| ARHGAP22 | 10 | 0.01499 | 49735563 | MIR4682 | 10 | 0.04299 | 121871353 |
| MAML2 | 11 | 0.01499 | 95766185 | SLC22A18 | 11 | 0.04299 | 2943671 |
| ANKRD12 | 18 | 0.01499 | 9255982 | CABP2 | 11 | 0.04299 | 67288594 |
| TIAM1 | 21 | 0.01499 | 32968981 | IGF1 | 12 | 0.04299 | 103152029 |
| ALK | 2 | 0.01499 | 29416366 | MAP1LC3B2 | 12 | 0.04299 | 117082846 |
| Gene | CHR | P | BP | Gene | CHR | P | BP |
| LOC101927829 | 3 | 0.01499 | 20995849 | CMKLR1 | 12 | 0.04299 | 108699032 |
| LCORL | 4 | 0.01499 | 19915956 | FLT1 | 13 | 0.04299 | 28929711 |
| LOC102546299 | 5 | 0.01499 | 164877178 | SEMA6D | 15 | 0.04299 | 48058071 |
| EYA4 | 6 | 0.01499 | 133655858 | DNAH2 | 17 | 0.04299 | 7735063 |
| NIPAL2 | 8 | 0.01499 | 99399509 | ARSG | 17 | 0.04299 | 66364804 |
| LINC00681 | 8 | 0.01499 | 12711863 | GALR1 | 18 | 0.04299 | 75579585 |
| PRRX2 | 9 | 0.01499 | 132444099 | APOBEC4 | 1 | 0.04299 | 183616926 |
| OR5B12 | 11 | 0.01515 | 58207203 | SPAG17 | 1 | 0.04299 | 118868405 |
| RBFOX1 | 16 | 0.01515 | 8088550 | COL4A4 | 2 | 0.04299 | 227867719 |
| ZNF233 | 19 | 0.01515 | 44778405 | LOC100506474 | 2 | 0.04299 | 14116991 |
| SPATA13 | 13 | 0.01522 | 24598384 | GPR35 | 2 | 0.04299 | 241558397 |
| MGA | 15 | 0.01522 | 42032383 | SERPINI1 | 3 | 0.04299 | 167578227 |
| SLC24A1 | 15 | 0.01522 | 65916527 | SPCS1 | 3 | 0.04299 | 52740182 |
| NKX2-5 | 5 | 0.01522 | 172737199 | MYRIP | 3 | 0.04299 | 40096618 |
| C9orf47 | 9 | 0.01522 | 91607869 | STPG2 | 4 | 0.04299 | 99037859 |
| CRTAM | 11 | 0.01818 | 122742028 | NMU | 4 | 0.04299 | 56621548 |
| CCND1 | 11 | 0.01977 | 69462910 | LOC340113 | 5 | 0.04299 | 33230034 |
| STXBP6 | 14 | 0.01977 | 26840301 | SND1 | 7 | 0.04299 | 127830114 |
| TAF1C | 16 | 0.01977 | 84213434 | IFRD1 | 7 | 0.04299 | 112087033 |
| TMEM171 | 5 | 0.01977 | 72419456 | ZMAT4 | 8 | 0.04299 | 40523282 |
| SH2D4B | 10 | 0.02288 | 82454295 | COL15A1 | 9 | 0.04299 | 101748234 |
| OR51I1 | 11 | 0.02288 | 5461991 | ARID3B | 15 | 0.04762 | 74888196 |
| LRRIQ1 | 12 | 0.02288 | 85466723 | NUP153 | 6 | 0.04762 | 17699322 |
| LINC00936 | 12 | 0.02288 | 90826178 | CASC21 | 8 | 0.04762 | 128323181 |
| LOC338797 | 12 | 0.02288 | 132085196 | RPSAP52 | 12 | 0.002879 | 66112855 |
| Gene | CHR | P | BP | Gene | CHR | P | BP |
| ATP11A | 13 | 0.02288 | 113479820 | LINC00466 | 1 | 0.002879 | 63493164 |
| PRSS54 | 16 | 0.02288 | 58314433 | FLJ34503 | 6 | 0.002997 | 114196015 |
| CKM | 19 | 0.02288 | 45810035 | LINC00476 | 9 | 0.002997 | 98482261 |
| FABP1 | 2 | 0.02288 | 88424066 | LOC102724710 | 8 | 0.004022 | 93201255 |
| CUL3 | 2 | 0.02288 | 225362478 | SERTM1 | 13 | 0.004662 | 37178919 |
| CACNA2D3 | 3 | 0.02288 | 55474073 | LOC101928858 | 5 | 0.004662 | 66538400 |
| TMEM215 | 9 | 0.02288 | 32784838 | GMNC | 3 | 0.004762 | 190480177 |
| PBX3 | 9 | 0.02288 | 128739400 | CBX3P2 | 18 | 0.006536 | 2631687 |
| STPG1 | 1 | 0.02301 | 24718075 | ZFPM2 | 8 | 0.006536 | 106142333 |
| ECE2 | 3 | 0.02301 | 183995341 | MIR3974 | 12 | 0.006993 | 17653834 |
| HLA-DQB2 | 6 | 0.02301 | 32767673 | CD276 | 15 | 0.007617 | 73929859 |
| LOC100507205 | 11 | 0.02482 | 42458745 | POM121L12 | 7 | 0.008242 | 51527696 |
| OR8A1 | 11 | 0.02482 | 124449980 | TLE3 | 15 | 0.009657 | 70208373 |
| LRRC4C | 11 | 0.02482 | 41294454 | CASC17 | 17 | 0.009657 | 68900605 |
| LOC102723968 | 13 | 0.02482 | 64585809 | TARBP1 | 1 | 0.009657 | 234524045 |
| CDKL1 | 14 | 0.02482 | 50799126 | LOC101928622 | 4 | 0.009657 | 32962030 |
| SPG7 | 16 | 0.02482 | 89587871 | LOC392232 | 8 | 0.009657 | 73106916 |
| TCN2 | 22 | 0.02482 | 31011610 | PPP2R3A | 3 | 0.0101 | 135288025 |
| PXDN | 2 | 0.02482 | 1777150 | SNX32 | 11 | 0.01084 | 65579670 |
| MIR3660 | 5 | 0.02482 | 89418270 | LINC00900 | 11 | 0.01136 | 115515326 |
| NFKBIL1 | 6 | 0.02482 | 31525319 | LOC339975 | 4 | 0.01136 | 187678866 |
| HLA-A | 6 | 0.02482 | 29927722 | LINC00365 | 13 | 0.01282 | 30670985 |
| SSR1 | 6 | 0.02482 | 7290437 | FAM27L | 17 | 0.01282 | 21717727 |
| TNPO3 | 7 | 0.02482 | 128597804 | SOD1 | 21 | 0.01499 | 32968981 |
| LOC100130298 | 8 | 0.02482 | 61955772 | VENTXP7 | 3 | 0.01499 | 20995849 |
| Gene | CHR | P | BP | Gene | CHR | P | BP |
| FANCC | 9 | 0.02482 | 97959500 | SLIT2 | 4 | 0.01499 | 19915956 |
| RGS3 | 9 | 0.02482 | 116478405 | CTB-7E3.1 | 5 | 0.01499 | 164877178 |
| PTEN | 10 | 0.02564 | 89912106 | KCNS2 | 8 | 0.01499 | 99399509 |
| PITRM1 | 10 | 0.02564 | 3202065 | KIAA1456 | 8 | 0.01499 | 12711863 |
| PLCE1 | 10 | 0.02564 | 96039597 | TMEM114 | 16 | 0.01515 | 8088550 |
| CFAP43 | 10 | 0.02564 | 105966404 | STC2 | 5 | 0.01522 | 172737199 |
| OR5B17 | 11 | 0.02564 | 58126305 | NOVA1 | 14 | 0.01977 | 26840301 |
| SLCO1B3 | 12 | 0.02564 | 21074122 | NRG3 | 10 | 0.02288 | 82454295 |
| SIX4 | 14 | 0.02564 | 61180657 | LINC00615 | 12 | 0.02288 | 90826178 |
| FSIP1 | 15 | 0.02564 | 40088761 | SFSWAP | 12 | 0.02288 | 132085196 |
| CCDC33 | 15 | 0.02564 | 74581073 | WNT5A | 3 | 0.02288 | 55474073 |
| ADAMTSL3 | 15 | 0.02564 | 84327771 | LOC101929116 | 9 | 0.02288 | 128739400 |
| RNF220 | 1 | 0.02564 | 45051656 | HLA-DOB | 6 | 0.02564 | 32770688 |
| UMODL1 | 21 | 0.02564 | 43510437 | HNRNPKP3 | 11 | 0.02482 | 42458745 |
| GNLY | 2 | 0.02564 | 85924729 | PANX3 | 11 | 0.02482 | 124449980 |
| KCNF1 | 2 | 0.02564 | 11114343 | LINC01052 | 13 | 0.02482 | 64585809 |
| KYNU | 2 | 0.02564 | 143743012 | MYT1L | 2 | 0.02482 | 1777150 |
| MAD2L1 | 4 | 0.02564 | 121429891 | LINC01339 | 5 | 0.02482 | 89418270 |
| ARRDC3-AS1 | 5 | 0.02564 | 91093966 | CLVS1 | 8 | 0.02482 | 61955772 |
| CDH18 | 5 | 0.02564 | 19771834 | ZNF618 | 9 | 0.02482 | 116478405 |
| CLINT1 | 5 | 0.02564 | 157551411 | RNLS | 10 | 0.02564 | 89912106 |
| VCAN | 5 | 0.02564 | 82889910 | SLCO1B7 | 12 | 0.02564 | 21074122 |
| KCNQ5 | 6 | 0.02564 | 73855954 | GPR176 | 15 | 0.02564 | 40088761 |
| AKAP12 | 6 | 0.02564 | 151670172 | FLJ33534 | 2 | 0.02564 | 11114343 |
| MAL2 | 8 | 0.02564 | 120224806 | PRDM5 | 4 | 0.02564 | 121429891 |
| Gene | CHR | P | BP | Gene | CHR | P | BP |
| AVPI1 | 10 | 0.03097 | 99439541 | NR2F1-AS1 | 5 | 0.02564 | 91093966 |
| ADARB2 | 10 | 0.03097 | 1462457 | LOC101927697 | 5 | 0.02564 | 157551411 |
| CNTN5 | 11 | 0.03097 | 100470306 | HAPLN1 | 5 | 0.02564 | 82889910 |
| OR51B2 | 11 | 0.03097 | 5344902 | LOC100128386 | 11 | 0.03097 | 100470306 |
| CEP126 | 11 | 0.03097 | 101832590 | LOC101928767 | 14 | 0.03097 | 86268812 |
| GGACT | 13 | 0.03097 | 101186056 | SLFN14 | 17 | 0.03097 | 33843512 |
| FLRT2 | 14 | 0.03097 | 86268812 | SKA1 | 18 | 0.03097 | 47837090 |
| SLFN12L | 17 | 0.03097 | 33843512 | LINC00323 | 21 | 0.03097 | 42511918 |
| DSC1 | 18 | 0.03097 | 28734749 | LARS2 | 3 | 0.03097 | 45276370 |
| RIT2 | 18 | 0.03097 | 40655271 | MIR548AB | 3 | 0.03097 | 102240202 |
| CXXC1 | 18 | 0.03097 | 47837090 | PCDH7 | 4 | 0.03097 | 29998461 |
| ZSCAN5B | 19 | 0.03097 | 56703248 | LOC729506 | 5 | 0.03097 | 8276128 |
| PLA2G4A | 1 | 0.03097 | 186946386 | GSE1 | 16 | 0.03251 | 85420064 |
| IL12RB2 | 1 | 0.03097 | 67800712 | HCG18 | 6 | 0.03251 | 30273371 |
| VAV3 | 1 | 0.03097 | 108313258 | HAS2 | 9 | 0.03251 | 121984358 |
| IL23R | 1 | 0.03097 | 67681669 | PLEKHG6 | 12 | 0.03333 | 6349328 |
| LIPI | 21 | 0.03097 | 15481365 | MPP6 | 7 | 0.03333 | 24355660 |
| DSCAM | 21 | 0.03097 | 42511918 | STAU2-AS1 | 8 | 0.03333 | 74274191 |
| TMEM158 | 3 | 0.03097 | 45276370 | LOC339622 | 21 | 0.03384 | 25808101 |
| FAM19A1 | 3 | 0.03097 | 68102663 | DDR1 | 6 | 0.03384 | 30821187 |
| ZPLD1 | 3 | 0.03097 | 102240202 | TBX5 | 12 | 0.03497 | 114484640 |
| MIR4275 | 4 | 0.03097 | 29998461 | TLE1 | 9 | 0.03497 | 84183462 |
| ARHGEF28 | 5 | 0.03097 | 73231666 | TUBGCP3 | 13 | 0.03571 | 113097722 |
| MTRR | 5 | 0.03097 | 8276128 | LINC00052 | 15 | 0.03571 | 87755594 |
| POC5 | 5 | 0.03097 | 75003678 | LCE5A | 1 | 0.03571 | 152454591 |
| Gene | CHR | P | BP | Gene | CHR | P | BP |
| NKAIN2 | 6 | 0.03097 | 124310564 | GPAM | 10 | 0.03595 | 113042093 |
| ZNRD1-AS1 | 6 | 0.04299 | 29954364 | PELI2 | 14 | 0.03595 | 56295580 |
| ITGAX | 16 | 0.03251 | 31374535 | HLA-DPA1 | 6 | 0.03595 | 33007752 |
| MIR5093 | 16 | 0.03251 | 85420064 | LINC00581 | 6 | 0.03595 | 21384613 |
| SHD | 19 | 0.03251 | 4283059 | ALDH18A1 | 10 | 0.04056 | 97348734 |
| TNNI1 | 1 | 0.03251 | 201386916 | LOC103312105 | 11 | 0.04056 | 37700681 |
| LINC01432 | 20 | 0.03251 | 22050503 | DPY19L2 | 12 | 0.04056 | 63789836 |
| AFF3 | 2 | 0.03251 | 100686486 | LINC00238 | 14 | 0.04056 | 66699865 |
| LOC101928519 | 6 | 0.03251 | 19129805 | PLA2G4B | 15 | 0.04056 | 42134097 |
| HCG17 | 6 | 0.03251 | 30273371 | MEX3B | 15 | 0.04056 | 81847279 |
| HCG27 | 6 | 0.03251 | 31167927 | BAIAP2-AS1 | 17 | 0.04056 | 78988362 |
| SNTB1 | 8 | 0.03251 | 121984358 | CSF1 | 1 | 0.04056 | 110346355 |
| CD9 | 12 | 0.03333 | 6349328 | LINC01247 | 2 | 0.04056 | 6500101 |
| SLC30A6 | 2 | 0.03333 | 32395315 | STAC | 3 | 0.04056 | 36377812 |
| LOC554223 | 6 | 0.03333 | 29775662 | UBE2E2 | 3 | 0.04056 | 22350158 |
| NPY | 7 | 0.03333 | 24355660 | LOC101929454 | 5 | 0.04056 | 14989346 |
| RDH10-AS1 | 8 | 0.03333 | 74274191 | RGMB | 5 | 0.04056 | 97469921 |
| OR51Q1 | 11 | 0.03384 | 5444353 | LOC100507477 | 6 | 0.04056 | 140197079 |
| CLEC16A | 16 | 0.03384 | 11229589 | HTR1B | 6 | 0.04056 | 77566201 |
| LOC101927869 | 21 | 0.03384 | 25808101 | DNAJC25-GNG10 | 9 | 0.04056 | 114382444 |
| SPAG16 | 2 | 0.03384 | 214867770 | PLPP4 | 10 | 0.04299 | 121871353 |
| ITGB6 | 2 | 0.03384 | 160957846 | LINC00485 | 12 | 0.04299 | 103152029 |
| CAPSL | 5 | 0.03384 | 35922462 | C12orf49 | 12 | 0.04299 | 117082846 |
| LINC00243 | 6 | 0.03384 | 30821187 | LINC01029 | 18 | 0.04299 | 75579585 |
| RBM19 | 12 | 0.03497 | 114484640 | TBX15 | 1 | 0.04299 | 118868405 |
| Gene | CHR | P | BP | Gene | CHR | P | BP |
| TPH2 | 12 | 0.03497 | 72407477 | LINC00276 | 2 | 0.04299 | 14116991 |
| CHMP4A | 14 | 0.03497 | 24679877 | LINC01330 | 3 | 0.04299 | 167578227 |
| LDHAL6B | 15 | 0.03497 | 59499179 | RAP1GDS1 | 4 | 0.04299 | 99076132 |
| HYDIN | 16 | 0.03497 | 71264561 | LOC644145 | 4 | 0.04299 | 56621548 |
| AFF1 | 4 | 0.03497 | 87924979 | TARS | 5 | 0.04299 | 33230034 |
| COL22A1 | 8 | 0.03497 | 139838912 | MIR129-1 | 7 | 0.04299 | 127830114 |
| LINC01507 | 9 | 0.03497 | 84183462 | CASC8 | 8 | 0.04762 | 128323181 |
| ANO1 | 11 | 0.03571 | 70002987 |  |  |  |  |

**Supplementary table 2.** **The differential genes between high grade and low grade endometrial stromal sarcomas in GSE85383.**

| id | logFC | AveExpr | t | P.Value | B | id | logFC | AveExpr | t | P.Value | B |
| --- | --- | --- | --- | --- | --- | --- | --- | --- | --- | --- | --- |
| JPH4 | -4.32123 | 3.013203 | -12.7237 | 9.47E-09 | 8.505724 | CASC8 | 1.036646 | -2.00895 | 2.677233 | 0.018899 | -3.16962 |
| TMEM176A | -3.43972 | 2.687578 | -10.7623 | 7.09E-08 | 7.229815 | TBX18 | 1.828921 | -0.6911 | 2.675503 | 0.018962 | -3.17257 |
| PRKCB | -3.71686 | 0.353816 | -10.1137 | 1.48E-07 | 6.727848 | PXDC1 | 0.810017 | 2.404609 | 2.67544 | 0.018964 | -3.17267 |
| TMEM176B | -4.09994 | 2.156868 | -8.54033 | 1.03E-06 | 5.307312 | FBXO2 | -2.0377 | 2.568546 | -2.67532 | 0.018968 | -3.17288 |
| SLC8A2 | -3.68713 | 0.318488 | -8.52584 | 1.05E-06 | 5.292757 | KIF4A | 1.508882 | 0.828296 | 2.67461 | 0.018994 | -3.17409 |
| ECRG4 | -3.42487 | 0.571165 | -8.47641 | 1.12E-06 | 5.24285 | FAM83C | 0.628859 | -1.76463 | 2.674419 | 0.019001 | -3.17441 |
| MGAT3 | -2.93784 | 1.530649 | -8.36652 | 1.29E-06 | 5.130681 | NIBAN3 | 0.816268 | -1.85035 | 2.674177 | 0.01901 | -3.17482 |
| SLC4A4 | 2.306867 | -0.09238 | 7.37102 | 5.18E-06 | 4.034002 | EPB42 | 0.50849 | -1.02781 | 2.672422 | 0.019074 | -3.17781 |
| FGF9 | 2.615748 | 0.706949 | 6.968512 | 9.40E-06 | 3.546955 | LINC00205 | 0.924814 | 0.74761 | 2.671037 | 0.019124 | -3.18017 |
| TC2N | -2.31312 | -0.60645 | -6.84261 | 1.14E-05 | 3.389187 | CLYBL | -0.83463 | 0.533955 | -2.67021 | 0.019154 | -3.18158 |
| LCA5L | -1.5053 | -1.45279 | -6.51505 | 1.89E-05 | 2.966328 | ZNF773 | 0.405 | 1.446587 | 2.669383 | 0.019185 | -3.18299 |
| TMEM215 | -3.59905 | 0.632208 | -6.36569 | 2.39E-05 | 2.767502 | LMF1 | 0.525506 | 1.616491 | 2.668954 | 0.0192 | -3.18372 |
| PDE8B | -2.76236 | 2.9522 | -6.34317 | 2.48E-05 | 2.737194 | MACROD1 | -0.6204 | 1.995826 | -2.66786 | 0.01924 | -3.18558 |
| PIANP | -2.44041 | 1.400915 | -6.21587 | 3.03E-05 | 2.564223 | VN1R1 | 1.365146 | -0.99167 | 2.667042 | 0.019271 | -3.18698 |
| RASL12 | -2.4691 | -0.88013 | -6.13758 | 3.44E-05 | 2.456465 | HEBP2 | -1.00069 | 4.61032 | -2.66704 | 0.019271 | -3.18698 |
| TCEAL2 | -3.33161 | 2.113961 | -6.09999 | 3.66E-05 | 2.404352 | TCEAL4 | -0.69554 | 5.486142 | -2.66471 | 0.019357 | -3.19095 |
| KCND3 | 0.962351 | -0.42953 | 2.681767 | 0.018736 | -3.16189 | DDOST | 0.355037 | 5.520345 | 2.160619 | 0.049826 | -4.02326 |
| MPPED1 | -2.1666 | 0.377228 | -2.68169 | 0.018739 | -3.16202 | ZSCAN31 | -0.90341 | 0.440818 | -2.16002 | 0.049881 | -4.02421 |
| TRIML2 | 1.453005 | -1.88158 | 2.681294 | 0.018753 | -3.16269 | THSD7A | -2.14263 | 0.145424 | -2.15951 | 0.049928 | -4.02501 |
| SGIP1 | -1.8055 | 1.090814 | -2.68104 | 0.018762 | -3.16314 | ERVW-1 | -0.41007 | -2.25614 | -2.1592 | 0.049956 | -4.0255 |
| GTF2A1 | -0.36216 | 2.826059 | -2.67984 | 0.018805 | -3.16517 | SIX4 | 1.103884 | 0.301445 | 2.159043 | 0.04997 | -4.02575 |
| LDLRAD3 | -0.61811 | -2.22943 | -2.67887 | 0.01884 | -3.16683 | NAALAD2 | -1.02887 | -1.90064 | -2.15889 | 0.049985 | -4.026 |
| CCDC160 | 1.094323 | -1.43904 | 2.677609 | 0.018886 | -3.16898 | ICAM2 | -1.86494 | 2.261298 | -2.15885 | 0.049988 | -4.02606 |
| KNTC1 | 0.944701 | 1.888925 | 2.677551 | 0.018888 | -3.16908 | SGPP2 | -2.09956 | -0.98836 | -2.15879 | 0.049993 | -4.02615 |
| id | logFC | AveExpr | t | P.Value | B | id | logFC | AveExpr | t | P.Value | B |
| APOE | -3.24659 | 5.810751 | -6.04366 | 4.01E-05 | 2.325812 | KREMEN1 | -1.04637 | 2.994157 | -2.66365 | 0.019396 | -3.19276 |
| HOXD13 | 2.008584 | -1.93153 | 6.00953 | 4.24E-05 | 2.277953 | SMG5 | 0.465615 | 3.645208 | 2.663155 | 0.019414 | -3.1936 |
| PSD | -1.2784 | 4.089715 | -5.99864 | 4.32E-05 | 2.262641 | SCN3B | -1.2207 | -0.07604 | -2.66312 | 0.019415 | -3.19365 |
| LRRC3 | -1.26409 | 0.759611 | -5.98221 | 4.43E-05 | 2.239499 | NECAB2 | -1.83181 | -0.22664 | -2.6627 | 0.019431 | -3.19438 |
| HSPA12B | -3.37405 | 0.796203 | -5.92967 | 4.83E-05 | 2.16519 | ZFHX4-AS1 | 0.645232 | -2.20856 | 2.660926 | 0.019497 | -3.19739 |
| CATIP | 1.33908 | -1.39926 | 5.832243 | 5.68E-05 | 2.026126 | TRIM68 | 0.423292 | 1.474352 | 2.660254 | 0.019522 | -3.19853 |
| MYH2 | 3.328563 | -0.62717 | 5.798107 | 6.01E-05 | 1.977016 | ALG13 | 0.637969 | 2.361972 | 2.658855 | 0.019574 | -3.20091 |
| PTP4A3 | -1.53357 | 1.928687 | -5.79027 | 6.09E-05 | 1.965706 | ZNF225 | 0.38669 | -0.16292 | 2.657976 | 0.019607 | -3.20241 |
| SNCA | -2.30158 | 1.859827 | -5.75809 | 6.43E-05 | 1.919193 | NBPF3 | 0.596792 | 0.191829 | 2.656704 | 0.019655 | -3.20457 |
| SPARCL1 | -5.44887 | 2.149036 | -5.68417 | 7.28E-05 | 1.811642 | DLX2 | 3.076325 | -0.21658 | 2.656316 | 0.019669 | -3.20523 |
| EBF4 | -1.87792 | 3.429496 | -5.66033 | 7.57E-05 | 1.776761 | ACSS3 | -2.1974 | 1.899875 | -2.65464 | 0.019732 | -3.20809 |
| CD6 | -2.88555 | -0.33993 | -5.64306 | 7.80E-05 | 1.751437 | LINC00312 | 2.05017 | 0.560633 | 2.654038 | 0.019755 | -3.20911 |
| ACP5 | -2.87913 | 3.484981 | -5.6318 | 7.95E-05 | 1.734886 | HBBP1 | 0.700156 | -1.08159 | 2.653568 | 0.019773 | -3.20991 |
| PTGDR2 | 1.152628 | -1.88383 | 5.467212 | 0.000105 | 1.490599 | WDR5B | 0.53191 | -0.7629 | 2.653443 | 0.019777 | -3.21012 |
| GNG7 | -1.98701 | 2.010497 | -5.41311 | 0.000115 | 1.409295 | GMFG | -2.09115 | 2.286885 | -2.65309 | 0.019791 | -3.21072 |
| FOXC2 | 2.33068 | -1.20972 | 5.410973 | 0.000116 | 1.40607 | KRTAP2-2 | 0.421321 | -2.17768 | 2.651612 | 0.019847 | -3.21324 |
| PEG3 | -3.00531 | -0.04781 | -5.36621 | 0.000125 | 1.338411 | MAGEH1 | -0.81392 | 3.601667 | -2.65138 | 0.019855 | -3.21363 |
| ST6GALNAC2 | -3.0127 | 1.31151 | -5.35449 | 0.000128 | 1.320631 | COL5A2 | 1.064867 | 4.987718 | 2.650892 | 0.019874 | -3.21446 |
| HOXB9 | 4.821517 | 0.705194 | 5.336873 | 0.000132 | 1.293881 | ZFHX4 | 2.363678 | -0.46859 | 2.649522 | 0.019926 | -3.21679 |
| NAP1L2 | -2.12275 | -0.95397 | -5.32454 | 0.000134 | 1.275113 | OPN5 | 0.365992 | -2.59659 | 2.649194 | 0.019938 | -3.21735 |
| RASL10B | -1.67108 | 1.32028 | -5.32051 | 0.000135 | 1.268979 | ASB6 | 0.638084 | 1.606144 | 2.648785 | 0.019954 | -3.21804 |
| STARD10 | -1.25389 | 2.735974 | -5.30254 | 0.00014 | 1.24159 | RCOR3 | 0.637093 | 1.330228 | 2.647118 | 0.020017 | -3.22088 |
| PALM | -1.09576 | 4.968433 | -5.29297 | 0.000142 | 1.226983 | N4BP3 | 0.575213 | -2.26398 | 2.646107 | 0.020056 | -3.2226 |
| TANC1 | 1.116543 | 3.626376 | 5.280678 | 0.000145 | 1.208185 | LINGO3 | -1.96217 | -1.23045 | -2.64605 | 0.020058 | -3.22269 |
| id | logFC | AveExpr | t | P.Value | B | id | logFC | AveExpr | t | P.Value | B |
| NMNAT3 | -2.31517 | -0.74307 | -5.25135 | 0.000153 | 1.163256 | METRNL | 0.807891 | 4.954236 | 2.645935 | 0.020063 | -3.22289 |
| C1QTNF4 | -2.80675 | 1.67902 | -5.21761 | 0.000162 | 1.111387 | NAA40 | 0.768158 | 0.235727 | 2.64356 | 0.020154 | -3.22692 |
| SEMA3D | 2.167048 | -0.71463 | 5.217036 | 0.000162 | 1.110498 | ARID3B | 0.927086 | 2.535178 | 2.643463 | 0.020157 | -3.22709 |
| NOVA2 | -2.42469 | 0.111099 | -5.17914 | 0.000173 | 1.052014 | TIGD1 | 0.809582 | 0.157304 | 2.643415 | 0.020159 | -3.22717 |
| SYNGR1 | -1.11966 | 1.866955 | -5.11825 | 0.000193 | 0.957539 | NRXN2 | -1.83087 | 2.351864 | -2.64274 | 0.020185 | -3.22831 |
| GAL | -3.10834 | 2.885191 | -5.11177 | 0.000195 | 0.947446 | RBPMS | -0.80186 | 3.277918 | -2.64156 | 0.020231 | -3.23032 |
| ANO1 | -2.26008 | -0.36114 | -5.09656 | 0.0002 | 0.923743 | EFNB3 | -1.45544 | 2.58371 | -2.64108 | 0.020249 | -3.23113 |
| PCSK1N | -1.78031 | 3.553611 | -5.0595 | 0.000214 | 0.865813 | SUFU | 0.723515 | 0.348398 | 2.640934 | 0.020255 | -3.23139 |
| RIMS2 | -2.05285 | 0.21618 | -5.05627 | 0.000215 | 0.860751 | FA2H | -1.32806 | -1.53887 | -2.64053 | 0.02027 | -3.23207 |
| HOXB3 | 2.863706 | -0.89407 | 4.99318 | 0.00024 | 0.761606 | ACOXL | -2.53933 | 0.344861 | -2.64009 | 0.020287 | -3.23282 |
| FZD7 | 1.948208 | 2.532654 | 4.99303 | 0.00024 | 0.76137 | IRF2BP1 | 0.398953 | 0.205655 | 2.638863 | 0.020335 | -3.2349 |
| MAGEB6 | 0.979712 | -2.30758 | 4.965359 | 0.000253 | 0.71768 | UST | 1.182819 | 0.527576 | 2.63825 | 0.020359 | -3.23594 |
| LRG1 | -1.48128 | -0.79766 | -4.93913 | 0.000265 | 0.676157 | LCN10 | -0.77993 | -2.16481 | -2.63785 | 0.020374 | -3.23662 |
| RSPO1 | -2.67152 | 0.07323 | -4.93047 | 0.000269 | 0.662421 | MTUS1 | -1.55391 | -0.52814 | -2.63754 | 0.020386 | -3.23716 |
| CASC15 | -2.37301 | 2.393567 | -4.9229 | 0.000272 | 0.650402 | GDAP1L1 | -2.01035 | -0.13628 | -2.63725 | 0.020398 | -3.23765 |
| ENTPD3 | -2.57405 | 0.783709 | -4.92078 | 0.000273 | 0.647045 | SLC19A3 | 0.541254 | -1.57361 | 2.637096 | 0.020404 | -3.2379 |
| NEIL1 | -2.02613 | 3.035084 | -4.91844 | 0.000275 | 0.643327 | ZIC5 | 2.643283 | -0.97375 | 2.63522 | 0.020477 | -3.24109 |
| ANKRD65 | -2.52059 | 0.691599 | -4.89987 | 0.000284 | 0.613802 | RNF39 | -1.39484 | -0.80091 | -2.63486 | 0.020491 | -3.24171 |
| MAOB | -3.5592 | 0.81941 | -4.89586 | 0.000286 | 0.607411 | VSIG10L | -0.82407 | 1.234573 | -2.63309 | 0.02056 | -3.2447 |
| ATF7IP2 | 1.476009 | 1.787612 | 4.862745 | 0.000303 | 0.554608 | SLC24A5 | 0.645087 | -0.95124 | 2.632958 | 0.020565 | -3.24493 |
| GNG8 | -2.18274 | 1.342566 | -4.86104 | 0.000304 | 0.551891 | ZNF814 | 0.688474 | 0.47736 | 2.631216 | 0.020633 | -3.24789 |
| ATP2A3 | -2.69529 | 0.514507 | -4.85086 | 0.00031 | 0.535608 | KYAT3 | -0.55793 | 1.845508 | -2.63086 | 0.020647 | -3.24849 |
| DUOX1 | -3.23047 | -0.3324 | -4.84529 | 0.000313 | 0.526699 | FAM167A-AS1 | -0.97687 | -2.05477 | -2.63078 | 0.020651 | -3.24862 |
| KCNQ1 | -3.04682 | 0.325525 | -4.83694 | 0.000318 | 0.513336 | ADARB1 | 0.603154 | 3.323448 | 2.629864 | 0.020687 | -3.25018 |
| id | logFC | AveExpr | t | P.Value | B | id | logFC | AveExpr | t | P.Value | B |
| C4BPB | 2.107545 | -1.63769 | 4.82104 | 0.000327 | 0.487863 | STK10 | 0.75328 | 2.594568 | 2.629859 | 0.020687 | -3.25019 |
| HOXC12 | 4.283664 | -1.11576 | 4.810716 | 0.000333 | 0.471298 | WWC2 | 1.102839 | 0.346119 | 2.629606 | 0.020697 | -3.25062 |
| TRIM34 | 0.894099 | 0.171761 | 4.80985 | 0.000334 | 0.469908 | LINC01139 | 0.456267 | -2.48443 | 2.628991 | 0.020721 | -3.25166 |
| NBR2 | 0.838215 | -0.1427 | 4.8096 | 0.000334 | 0.469507 | ADGRE1 | 0.744164 | 0.402689 | 2.628805 | 0.020728 | -3.25198 |
| STAP2 | -1.76159 | 2.00557 | -4.78477 | 0.000349 | 0.429592 | OR2AE1 | -0.64257 | -2.15795 | -2.62873 | 0.020732 | -3.25211 |
| ZNF470 | 0.735401 | 0.648747 | 4.781455 | 0.000351 | 0.424262 | CEP250 | 0.403101 | 2.403417 | 2.627488 | 0.020781 | -3.25421 |
| ADGRL2 | 1.198494 | 1.601743 | 4.779006 | 0.000353 | 0.42032 | SULT1C3 | -0.7923 | -1.98949 | -2.6266 | 0.020816 | -3.25571 |
| SEC14L6 | -2.27958 | -0.59632 | -4.76582 | 0.000361 | 0.399072 | CNTNAP3 | 1.734723 | -0.43595 | 2.626541 | 0.020818 | -3.25582 |
| TSHZ2 | -2.32408 | 1.35811 | -4.75828 | 0.000366 | 0.386923 | BMP8A | -1.65877 | 0.147949 | -2.62629 | 0.020828 | -3.25624 |
| BEX2 | -2.9393 | 2.660413 | -4.75558 | 0.000368 | 0.382564 | NAP1L3 | -0.80399 | 0.850925 | -2.62569 | 0.020852 | -3.25726 |
| FOXL1 | 3.020972 | -0.22346 | 4.750783 | 0.000371 | 0.374819 | MRPL34 | -0.45498 | 4.685747 | -2.62568 | 0.020852 | -3.25728 |
| FSTL1 | 1.334726 | 6.911552 | 4.744646 | 0.000375 | 0.36491 | TANK | -0.45157 | 3.091441 | -2.62545 | 0.020861 | -3.25767 |
| DCLK2 | 1.403677 | 1.256374 | 4.718465 | 0.000394 | 0.322569 | ALG9 | 0.526356 | 0.782834 | 2.62496 | 0.020881 | -3.2585 |
| FAM99B | 1.443102 | -1.71818 | 4.710488 | 0.000399 | 0.309649 | SAA4 | 0.633624 | -1.79604 | 2.624231 | 0.02091 | -3.25974 |
| A1BG | -0.87467 | 3.856926 | -4.70839 | 0.000401 | 0.306247 | KLHL20 | 0.425336 | 1.740517 | 2.624003 | 0.020919 | -3.26012 |
| CEP83-DT | 1.467191 | -1.43854 | 4.706182 | 0.000402 | 0.30267 | TDG | 0.457829 | 3.339738 | 2.623898 | 0.020923 | -3.2603 |
| ZNF560 | 1.560694 | -1.9327 | 4.700361 | 0.000407 | 0.293232 | TMEM161B | 0.420797 | 2.06784 | 2.622075 | 0.020996 | -3.26339 |
| GPR4 | -3.498 | 0.347468 | -4.69371 | 0.000412 | 0.282438 | XRCC4 | 0.645695 | 1.554667 | 2.621929 | 0.021002 | -3.26364 |
| GATM | -2.27209 | -0.43928 | -4.65432 | 0.000442 | 0.218396 | MAL2 | -2.46 | 0.243736 | -2.62182 | 0.021006 | -3.26382 |
| RAB3IL1 | -1.61005 | 2.196934 | -4.65035 | 0.000445 | 0.211935 | CDCA7 | 0.848982 | 1.86464 | 2.621084 | 0.021036 | -3.26508 |
| SPINT1 | -2.5508 | 1.55755 | -4.63663 | 0.000457 | 0.189573 | MMS22L | 0.740302 | -0.11091 | 2.617927 | 0.021162 | -3.27043 |
| DRD2 | -2.72254 | 0.433759 | -4.63221 | 0.00046 | 0.182356 | SMYD2 | 0.533698 | 2.671906 | 2.617838 | 0.021166 | -3.27058 |
| CLDN5 | -5.38533 | 1.426682 | -4.6251 | 0.000466 | 0.170758 | LOC145474 | 1.225701 | 1.31086 | 2.61776 | 0.021169 | -3.27071 |
| WT1 | -2.48291 | 1.698288 | -4.61224 | 0.000477 | 0.149738 | CFAP73 | -0.68463 | -1.35646 | -2.61765 | 0.021174 | -3.2709 |
| CYYR1 | -2.97836 | -0.66121 | -4.6081 | 0.000481 | 0.142969 | SERPINF2 | 0.500197 | -1.24295 | 2.616796 | 0.021208 | -3.27234 |
| id | logFC | AveExpr | t | P.Value | B | id | logFC | AveExpr | t | P.Value | B |
| RAB42 | -1.92036 | 0.910976 | -4.57492 | 0.000511 | 0.08863 | ARHGAP20 | 0.789715 | 1.18049 | 2.615981 | 0.021241 | -3.27373 |
| GLP2R | 0.680988 | -2.00023 | 4.564116 | 0.000521 | 0.070906 | MAGI1 | 0.734427 | -0.32217 | 2.615234 | 0.021271 | -3.27499 |
| ESR1 | -2.93883 | 0.959449 | -4.56257 | 0.000523 | 0.068373 | GNG4 | -1.2858 | 2.652005 | -2.61517 | 0.021274 | -3.27509 |
| PKIG | -0.97699 | 2.708218 | -4.55033 | 0.000535 | 0.048267 | AQP10 | 0.729786 | -0.88775 | 2.614757 | 0.021291 | -3.2758 |
| TVP23A | -1.79189 | -0.84987 | -4.54504 | 0.00054 | 0.039565 | LINC00607 | 0.925186 | -2.06144 | 2.614256 | 0.021311 | -3.27665 |
| SLC40A1 | -3.76803 | 1.566393 | -4.53513 | 0.00055 | 0.02326 | NEURL1 | -0.98469 | 1.532779 | -2.61399 | 0.021322 | -3.2771 |
| HOXB5 | 4.117802 | 0.404126 | 4.522559 | 0.000563 | 0.002565 | EVI5 | 0.569201 | 0.860374 | 2.613682 | 0.021334 | -3.27762 |
| WFDC2 | -3.34195 | 1.977692 | -4.50686 | 0.000579 | -0.02331 | ADAM15 | 0.826164 | 3.079309 | 2.612988 | 0.021362 | -3.2788 |
| DAPK2 | -1.92032 | 1.549533 | -4.47169 | 0.000618 | -0.08142 | DOCK7 | 0.502991 | 2.575127 | 2.61297 | 0.021363 | -3.27883 |
| LRRC6 | -1.05473 | 0.470687 | -4.46057 | 0.000631 | -0.09983 | TREM1 | -2.62307 | 0.795802 | -2.61189 | 0.021407 | -3.28065 |
| GPR162 | -1.50569 | 3.511606 | -4.45996 | 0.000631 | -0.10083 | CDKN1B | 1.017438 | 1.531183 | 2.610092 | 0.021481 | -3.2837 |
| FZD1 | 1.312512 | 2.59451 | 4.453925 | 0.000638 | -0.11083 | FMO6P | 0.856719 | -1.18419 | 2.609929 | 0.021487 | -3.28398 |
| PRDM1 | -2.55115 | 1.489532 | -4.4373 | 0.000658 | -0.13839 | ZMYND12 | -1.03848 | -0.83002 | -2.60873 | 0.021536 | -3.28601 |
| CCNT2-AS1 | 1.107658 | -1.40379 | 4.43415 | 0.000662 | -0.14362 | DHX35 | 0.482271 | 2.913841 | 2.607527 | 0.021586 | -3.28805 |
| ASPG | -3.06036 | 0.184796 | -4.43265 | 0.000664 | -0.1461 | FAM133B | 0.495928 | 2.424013 | 2.607397 | 0.021591 | -3.28827 |
| IGF2BP2 | 1.364646 | 1.591075 | 4.432439 | 0.000664 | -0.14646 | CENPF | 1.432413 | 3.470989 | 2.607251 | 0.021597 | -3.28851 |
| RIC3 | -1.95571 | -1.12429 | -4.43065 | 0.000666 | -0.14942 | ARRB1 | -0.79676 | 0.598935 | -2.60721 | 0.021599 | -3.28858 |
| SGCD | 2.089144 | 0.78057 | 4.394381 | 0.000713 | -0.20972 | FABP7 | 0.365323 | -2.20191 | 2.606357 | 0.021634 | -3.29003 |
| SYT17 | -2.32517 | 0.227706 | -4.38535 | 0.000725 | -0.22475 | ADAMTS8 | -2.00947 | 1.336648 | -2.60559 | 0.021665 | -3.29133 |
| FLRT2 | 1.375813 | 0.471356 | 4.381637 | 0.00073 | -0.23095 | GALNT2 | 1.357778 | 1.827821 | 2.604473 | 0.021711 | -3.29322 |
| PYCARD | -1.64378 | 3.739015 | -4.37459 | 0.000739 | -0.24269 | ANKRD26P3 | 0.883615 | -2.25519 | 2.604024 | 0.02173 | -3.29398 |
| KNDC1 | -1.35805 | -0.47269 | -4.37176 | 0.000743 | -0.24741 | PANK4 | 0.50732 | 1.529454 | 2.603935 | 0.021734 | -3.29413 |
| TMEM74B | -2.18496 | 1.882816 | -4.36964 | 0.000746 | -0.25095 | ACTN3 | -1.19107 | -1.01863 | -2.60242 | 0.021797 | -3.2967 |
| EBF2 | 2.140815 | -0.76111 | 4.338359 | 0.000791 | -0.30318 | GPAM | 0.810188 | 1.4384 | 2.601997 | 0.021814 | -3.29741 |
| id | logFC | AveExpr | t | P.Value | B | id | logFC | AveExpr | t | P.Value | B |
| LGALS3 | -1.43548 | 5.424195 | -4.33193 | 0.0008 | -0.31393 | NECTIN4 | 1.070275 | -0.59045 | 2.601121 | 0.02185 | -3.29889 |
| XG | 2.766188 | -0.76604 | 4.329462 | 0.000804 | -0.31806 | SETD2 | 0.389322 | 1.763204 | 2.599605 | 0.021913 | -3.30146 |
| KCNK4 | -3.48197 | 1.79751 | -4.31549 | 0.000825 | -0.34146 | FLJ30679 | 0.642048 | -2.14185 | 2.599502 | 0.021918 | -3.30163 |
| HOXB4 | 1.860365 | 0.561331 | 4.309165 | 0.000835 | -0.35205 | EN1 | 1.632544 | -1.11602 | 2.599273 | 0.021927 | -3.30202 |
| SCUBE2 | -2.51391 | 1.613111 | -4.30011 | 0.000849 | -0.36723 | DECR2 | 0.486838 | 3.17714 | 2.59817 | 0.021973 | -3.30388 |
| XPR1 | 1.206629 | 1.559512 | 4.290942 | 0.000863 | -0.3826 | PRR27 | 1.063633 | -1.32707 | 2.598035 | 0.021979 | -3.30411 |
| CDK6 | 1.901655 | 2.114753 | 4.290302 | 0.000864 | -0.38368 | DIRAS1 | -1.53451 | 3.468169 | -2.59785 | 0.021987 | -3.30443 |
| CDKL1 | -1.31466 | 1.720534 | -4.28986 | 0.000865 | -0.38442 | LDHD | -1.16316 | 1.017132 | -2.59749 | 0.022002 | -3.30504 |
| TCEAL5 | -2.74247 | 1.594956 | -4.28591 | 0.000872 | -0.39105 | PITPNM3 | -1.08492 | 0.649346 | -2.59747 | 0.022003 | -3.30506 |
| HOXC5 | 2.127974 | -0.22841 | 4.254604 | 0.000924 | -0.44366 | ZNF695 | 1.354206 | -0.70564 | 2.597053 | 0.02202 | -3.30577 |
| HID1 | -2.74434 | 2.2801 | -4.24445 | 0.000942 | -0.46075 | NAGS | -1.07466 | 2.393141 | -2.59572 | 0.022076 | -3.30804 |
| SRGAP2 | 1.47975 | 3.85166 | 4.243779 | 0.000943 | -0.46187 | ELAPOR2 | 1.346824 | 0.632694 | 2.595629 | 0.02208 | -3.30818 |
| CLIC6 | -2.54773 | -0.30299 | -4.24375 | 0.000943 | -0.46192 | CRIP3 | -1.44755 | -0.26082 | -2.59519 | 0.022098 | -3.30892 |
| SPANXN3 | 0.729781 | -2.22393 | 4.239877 | 0.00095 | -0.46845 | FCRL5 | 0.910912 | -1.9357 | 2.594551 | 0.022125 | -3.31001 |
| KLHL29 | 1.093126 | 3.304524 | 4.225509 | 0.000976 | -0.49266 | KLHL12 | 0.486027 | 1.502742 | 2.594321 | 0.022135 | -3.3104 |
| INTS7 | 0.971997 | 0.692476 | 4.225157 | 0.000976 | -0.49325 | SERTAD1 | -0.56517 | 4.906521 | -2.59382 | 0.022156 | -3.31124 |
| SSX7 | 0.65828 | -2.16812 | 4.21687 | 0.000991 | -0.50722 | PIGS | 0.564615 | 4.095052 | 2.593769 | 0.022158 | -3.31133 |
| HEMGN | 0.723641 | -2.17342 | 4.213311 | 0.000998 | -0.51323 | AKR1E2 | 1.050609 | -1.05044 | 2.592805 | 0.022199 | -3.31296 |
| CACNB4 | 1.780702 | -0.29417 | 4.205753 | 0.001012 | -0.52598 | RDH13 | -0.48857 | 1.863145 | -2.59198 | 0.022234 | -3.31436 |
| SLC1A6 | -2.53955 | -0.41845 | -4.19017 | 0.001042 | -0.5523 | PGF | -2.06437 | 2.622878 | -2.59143 | 0.022257 | -3.31528 |
| ITGB4 | -2.32341 | 1.321864 | -4.18987 | 0.001043 | -0.55282 | ANKRD35 | -2.23266 | 2.988341 | -2.59066 | 0.02229 | -3.31659 |
| CRYGN | -2.00245 | 1.315735 | -4.17584 | 0.001071 | -0.57653 | XPO4 | 0.454194 | 0.274749 | 2.590297 | 0.022305 | -3.3172 |
| RUBCNL | -2.30487 | 0.688418 | -4.17507 | 0.001072 | -0.57784 | NEK11 | -1.15685 | -0.08897 | -2.58996 | 0.022319 | -3.31777 |
| LAMC3 | -2.79957 | 2.805553 | -4.17198 | 0.001078 | -0.58306 | SCGB1A1 | 0.401906 | 0.762387 | 2.589944 | 0.02232 | -3.3178 |
| PRL | -4.904 | 1.793253 | -4.16225 | 0.001098 | -0.59954 | TPBG | 1.143575 | 3.431534 | 2.588498 | 0.022381 | -3.32024 |
| id | logFC | AveExpr | t | P.Value | B | id | logFC | AveExpr | t | P.Value | B |
| PRSS50 | -1.75528 | -1.35612 | -4.15673 | 0.00111 | -0.60888 | SYTL1 | -1.66281 | 2.084207 | -2.5884 | 0.022386 | -3.32041 |
| FMNL1 | -1.33643 | 1.028167 | -4.14408 | 0.001136 | -0.63031 | FAM189B | 0.599756 | 3.318122 | 2.588215 | 0.022393 | -3.32072 |
| RPL34-DT | 1.249126 | -1.84073 | 4.131931 | 0.001163 | -0.65092 | MED31 | 0.66689 | -0.12561 | 2.587305 | 0.022432 | -3.32226 |
| RASAL1 | -2.11667 | 0.209079 | -4.12538 | 0.001177 | -0.66203 | ATMIN | -0.38863 | 2.042482 | -2.58708 | 0.022442 | -3.32263 |
| CNR1 | -3.58334 | -0.13184 | -4.11661 | 0.001196 | -0.67693 | NUF2 | 1.562213 | 1.22795 | 2.586099 | 0.022484 | -3.32429 |
| NLRP5 | 1.018912 | -1.44793 | 4.115063 | 0.0012 | -0.67955 | FGD5 | -2.55685 | 2.307608 | -2.5852 | 0.022522 | -3.32581 |
| C1orf115 | -2.03047 | -0.22606 | -4.10804 | 0.001216 | -0.69147 | KLHL13 | -1.42809 | 0.696949 | -2.58498 | 0.022532 | -3.32619 |
| OR3A1 | 1.004665 | -2.16682 | 4.099293 | 0.001236 | -0.70635 | MTFR2 | 1.214005 | 0.002217 | 2.584931 | 0.022534 | -3.32627 |
| WDR86 | -2.701 | 3.507527 | -4.08527 | 0.001269 | -0.73019 | HAVCR1 | 0.624869 | -1.88566 | 2.583616 | 0.02259 | -3.32849 |
| CCDC88B | -1.61417 | 2.28902 | -4.08461 | 0.001271 | -0.73132 | UTF1 | -1.28642 | 3.1241 | -2.583 | 0.022616 | -3.32953 |
| TEPP | -2.01663 | -1.26899 | -4.07971 | 0.001282 | -0.73965 | GDF9 | 0.441742 | -2.12934 | 2.582955 | 0.022618 | -3.3296 |
| A4GALT | -1.46713 | 2.805032 | -4.07081 | 0.001304 | -0.75481 | HERC2 | 0.396424 | 2.400811 | 2.582942 | 0.022619 | -3.32963 |
| ZFYVE21 | -0.69471 | 4.926868 | -4.06324 | 0.001323 | -0.76771 | GPR20 | -1.6816 | 2.493266 | -2.5829 | 0.022621 | -3.32969 |
| MYZAP | -1.97009 | 0.19756 | -4.06016 | 0.001331 | -0.77296 | DAPK1 | 1.076182 | -0.261 | 2.582013 | 0.022659 | -3.3312 |
| SNAI3 | -2.06698 | -1.10836 | -4.05836 | 0.001335 | -0.77602 | TTBK1 | -1.78272 | -0.24222 | -2.58154 | 0.022679 | -3.332 |
| GNA14 | -2.01513 | -0.2897 | -4.05831 | 0.001335 | -0.77612 | PARP8 | 0.681779 | 0.906966 | 2.580422 | 0.022728 | -3.33388 |
| GABRA1 | 1.037078 | -2.28191 | 4.055877 | 0.001341 | -0.78026 | HDAC1 | -0.46954 | 4.430983 | -2.5801 | 0.022741 | -3.33443 |
| SPTBN4 | -1.12727 | 0.516906 | -4.05303 | 0.001349 | -0.78511 | RAD21-AS1 | 0.809684 | -2.01705 | 2.579883 | 0.022751 | -3.33479 |
| LARP1B | 0.904465 | 0.061132 | 4.047595 | 0.001363 | -0.79438 | LPCAT2 | -1.48207 | 1.290652 | -2.57896 | 0.022791 | -3.33635 |
| DMTN | -1.69439 | 2.160175 | -4.04523 | 0.001369 | -0.79841 | MGAT5 | 0.915733 | 0.916304 | 2.577978 | 0.022833 | -3.33801 |
| KLK1 | -2.53914 | -0.60026 | -4.0311 | 0.001406 | -0.82253 | CSDC2 | -1.08621 | 2.143928 | -2.57753 | 0.022853 | -3.33876 |
| ANXA10 | 2.002815 | -1.47142 | 4.028954 | 0.001411 | -0.82619 | NUP88 | 0.37312 | 2.669085 | 2.577489 | 0.022854 | -3.33883 |
| CRIM1-DT | 1.473987 | 2.769218 | 4.026105 | 0.001419 | -0.83105 | VAMP5 | -0.6239 | 3.270952 | -2.57701 | 0.022875 | -3.33964 |
| CEACAM5 | 0.940029 | -2.22922 | 4.024125 | 0.001424 | -0.83444 | STAG3 | 1.003078 | 0.591067 | 2.576948 | 0.022878 | -3.33975 |
| HOXC10 | 2.440918 | -1.53814 | 4.018154 | 0.00144 | -0.84464 | P4HA1 | 1.237291 | 2.194957 | 2.575613 | 0.022936 | -3.342 |
| id | logFC | AveExpr | t | P.Value | B | id | logFC | AveExpr | t | P.Value | B |
| MCOLN3 | -2.31307 | 0.416238 | -4.01714 | 0.001443 | -0.84636 | TGM2 | -2.95263 | 3.936677 | -2.5752 | 0.022954 | -3.34271 |
| PRKAR1A | -0.63719 | 1.809405 | -4.00668 | 0.001472 | -0.86424 | SMIM1 | -1.88767 | 1.622044 | -2.57388 | 0.023012 | -3.34493 |
| ASAP2 | 0.986083 | 2.193494 | 4.003433 | 0.001481 | -0.8698 | PLCXD1 | 0.572311 | 2.044707 | 2.57371 | 0.023019 | -3.34521 |
| PTX3 | 3.590097 | 1.95174 | 3.98969 | 0.00152 | -0.89331 | P2RY2 | -1.69024 | -0.11096 | -2.5722 | 0.023085 | -3.34777 |
| HS3ST3B1 | 1.647686 | -0.77155 | 3.988262 | 0.001524 | -0.89575 | ARHGDIG | -2.18959 | 1.110678 | -2.57161 | 0.023111 | -3.34876 |
| RGL3 | -2.32381 | 1.382353 | -3.98626 | 0.00153 | -0.89919 | DOK7 | -2.61237 | 0.299835 | -2.57085 | 0.023145 | -3.35004 |
| HOXB6 | 4.140039 | 1.120209 | 3.979848 | 0.001549 | -0.91015 | KRTAP13-2 | 0.90896 | -1.32352 | 2.57074 | 0.023149 | -3.35023 |
| LYPD6 | 1.314572 | 0.572422 | 3.975454 | 0.001562 | -0.91768 | SNHG10 | 0.887973 | -1.81385 | 2.570162 | 0.023175 | -3.3512 |
| CLCN4 | 1.443756 | -0.13572 | 3.963341 | 0.001598 | -0.93843 | ABHD6 | -0.39967 | 2.603825 | -2.5696 | 0.023199 | -3.35214 |
| FLVCR1-DT | 1.504208 | 0.119796 | 3.962377 | 0.001601 | -0.94008 | GSG1L | -1.51632 | -1.2478 | -2.5686 | 0.023244 | -3.35384 |
| PACSIN1 | -3.15945 | 1.185196 | -3.96164 | 0.001603 | -0.94134 | B3GNT3 | -1.72321 | 0.894287 | -2.56742 | 0.023296 | -3.35583 |
| ZNF404 | 1.132865 | 1.039616 | 3.951625 | 0.001634 | -0.95852 | UGT3A1 | 0.417028 | -0.46712 | 2.566947 | 0.023317 | -3.35662 |
| CCDC184 | -1.31179 | -0.83124 | -3.9385 | 0.001675 | -0.98103 | ENAH | 0.972333 | 2.942875 | 2.56589 | 0.023364 | -3.35841 |
| HTRA3 | -1.69369 | 1.046372 | -3.928 | 0.001708 | -0.99906 | NXF3 | 0.749364 | 0.358906 | 2.564813 | 0.023411 | -3.36022 |
| PRR5-ARHGAP8 | -1.58155 | 0.590863 | -3.91629 | 0.001747 | -1.01917 | H19 | -2.86721 | 2.212523 | -2.56464 | 0.023419 | -3.36051 |
| GALNT7 | 0.78018 | 2.647791 | 3.906966 | 0.001778 | -1.03519 | DUOXA1 | -1.65889 | 1.347226 | -2.56187 | 0.023542 | -3.36518 |
| CCKBR | -1.92721 | 0.077384 | -3.90545 | 0.001783 | -1.0378 | LIG3 | 0.513313 | 1.200849 | 2.560648 | 0.023597 | -3.36724 |
| CPNE2 | -0.87431 | 2.880043 | -3.90151 | 0.001797 | -1.04457 | EGR4 | -2.28592 | 1.327454 | -2.56029 | 0.023613 | -3.36785 |
| NUDT13 | 0.781257 | -0.12145 | 3.894116 | 0.001822 | -1.05729 | HAS3 | 1.361177 | 0.065055 | 2.55982 | 0.023634 | -3.36864 |
| PTPMT1 | -1.02682 | 3.253806 | -3.88405 | 0.001857 | -1.07461 | TNFRSF14 | -1.25038 | 2.396565 | -2.55962 | 0.023643 | -3.36898 |
| SHE | -2.19179 | -0.64076 | -3.88374 | 0.001858 | -1.07514 | IGFBP1 | -4.22837 | 1.1836 | -2.5591 | 0.023667 | -3.36985 |
| BAG1 | -0.7281 | 2.57556 | -3.87686 | 0.001883 | -1.08699 | SEPTIN6 | -0.85616 | 2.049122 | -2.55907 | 0.023668 | -3.36991 |
| CASC2 | -0.96951 | -1.30179 | -3.87427 | 0.001892 | -1.09144 | RTP1 | 0.735661 | -1.90129 | 2.558983 | 0.023672 | -3.37005 |
| MYO1B | 0.783898 | 0.499638 | 3.87387 | 0.001893 | -1.09213 | GBAP1 | 0.921603 | 2.50873 | 2.558891 | 0.023676 | -3.3702 |
| id | logFC | AveExpr | t | P.Value | B | id | logFC | AveExpr | t | P.Value | B |
| GARNL3 | -1.6002 | 1.569363 | -3.87301 | 0.001897 | -1.09361 | RCSD1 | -1.93871 | -0.81489 | -2.55878 | 0.023681 | -3.37038 |
| CCSAP | 1.182762 | 1.106223 | 3.868897 | 0.001911 | -1.1007 | GTPBP10 | 0.781687 | -0.97767 | 2.556781 | 0.023771 | -3.37376 |
| MAMDC4 | 1.060679 | 1.923746 | 3.865838 | 0.001923 | -1.10596 | ITGA6 | 0.801823 | 1.271153 | 2.556212 | 0.023797 | -3.37471 |
| ARHGEF3 | -1.74075 | 1.601633 | -3.86099 | 0.00194 | -1.11432 | SLC6A2 | -2.04326 | -0.67006 | -2.55601 | 0.023806 | -3.37505 |
| SLC22A16 | -2.31289 | 0.017872 | -3.86043 | 0.001943 | -1.11529 | DEFB129 | 0.470809 | -1.11438 | 2.555652 | 0.023822 | -3.37566 |
| BCL2L10 | -1.33728 | -0.22918 | -3.85935 | 0.001946 | -1.11713 | EPHB3 | 0.844299 | 2.566625 | 2.555209 | 0.023842 | -3.3764 |
| CIB2 | -1.21419 | 2.7734 | -3.85799 | 0.001952 | -1.11948 | BCL6B | -2.03143 | -0.91143 | -2.55501 | 0.023851 | -3.37674 |
| FNDC11 | -1.84372 | 0.204018 | -3.85093 | 0.001978 | -1.13165 | RBFOX1 | -1.31976 | -0.93157 | -2.55491 | 0.023855 | -3.3769 |
| CFAP91 | -1.68509 | -1.25227 | -3.8418 | 0.002013 | -1.14739 | SLC25A35 | 0.920888 | -0.27528 | 2.554166 | 0.023889 | -3.37816 |
| FLJ46906 | 1.250236 | 3.588449 | 3.84088 | 0.002016 | -1.14898 | SH3KBP1 | 0.549184 | 2.471668 | 2.554118 | 0.023891 | -3.37824 |
| SIPA1L1 | 0.947021 | 3.960664 | 3.836786 | 0.002032 | -1.15604 | CEBPB | -0.87208 | 5.209745 | -2.55395 | 0.023899 | -3.37852 |
| LINC00472 | 1.50994 | -1.51012 | 3.832615 | 0.002048 | -1.16324 | OXER1 | -1.11688 | 1.308868 | -2.55384 | 0.023904 | -3.37872 |
| INHBA-AS1 | 1.18046 | -1.78784 | 3.831054 | 0.002054 | -1.16593 | LOC100128361 | 0.548409 | 0.514167 | 2.553347 | 0.023926 | -3.37954 |
| BSPRY | -3.73307 | 0.122058 | -3.83092 | 0.002055 | -1.16615 | LTB4R | 0.746863 | 1.106888 | 2.552678 | 0.023957 | -3.38067 |
| SLC52A1 | -2.19169 | 0.780127 | -3.82833 | 0.002065 | -1.17063 | SKIV2L | 0.390827 | 3.342894 | 2.551058 | 0.024031 | -3.38339 |
| SCARF2 | 1.13218 | 3.292753 | 3.825189 | 0.002077 | -1.17605 | GMCL1 | 0.546801 | 0.711936 | 2.550864 | 0.024039 | -3.38372 |
| ALOX5 | -3.3864 | 0.31439 | -3.82201 | 0.00209 | -1.18153 | C10orf62 | 0.363481 | -2.18706 | 2.55034 | 0.024063 | -3.3846 |
| TPPP3 | -1.85481 | 1.075098 | -3.82177 | 0.002091 | -1.18195 | DARS2 | 0.859951 | 0.243381 | 2.54925 | 0.024113 | -3.38644 |
| CBLN1 | -2.97936 | 0.481834 | -3.81245 | 0.002128 | -1.19805 | SPC24 | 1.49644 | -0.69255 | 2.54914 | 0.024118 | -3.38662 |
| LOC646903 | 1.412702 | -0.17714 | 3.812264 | 0.002129 | -1.19836 | PLA2G7 | -2.17594 | -0.31881 | -2.54911 | 0.02412 | -3.38667 |
| FAM131B | -1.66906 | 0.379707 | -3.81048 | 0.002136 | -1.20143 | CNMD | -2.24832 | 0.351451 | -2.54716 | 0.024209 | -3.38995 |
| MIDEAS | 0.62736 | 2.257589 | 3.809373 | 0.002141 | -1.20335 | RILPL2 | -0.72112 | 3.418314 | -2.54617 | 0.024254 | -3.39161 |
| MB21D2 | 1.33807 | 0.291985 | 3.808882 | 0.002143 | -1.2042 | ICOS | -0.90704 | -1.23533 | -2.545 | 0.024308 | -3.39359 |
| MAGEB2 | 1.543152 | -0.78932 | 3.808453 | 0.002145 | -1.20494 | FADS6 | -1.67671 | -1.01377 | -2.54228 | 0.024434 | -3.39815 |
| id | logFC | AveExpr | t | P.Value | B | id | logFC | AveExpr | t | P.Value | B |
| PAX6 | 2.388813 | -0.9203 | 3.802883 | 0.002168 | -1.21456 | DLGAP1  -AS1 | -0.74959 | 3.186418 | -2.54196 | 0.024449 | -3.39871 |
| PRLR | -3.15949 | -0.02327 | -3.80284 | 0.002168 | -1.21464 | TYSND1 | 0.580617 | 0.221144 | 2.541944 | 0.024449 | -3.39873 |
| SCN9A | 2.114309 | -1.17769 | 3.801186 | 0.002175 | -1.21749 | E2F3 | 0.538608 | 2.489975 | 2.541448 | 0.024472 | -3.39956 |
| RSKR | 0.999897 | 0.37956 | 3.794028 | 0.002204 | -1.22986 | CAMKV | -1.31296 | -0.61923 | -2.5397 | 0.024554 | -3.4025 |
| FOCAD | 0.846267 | 4.725224 | 3.791858 | 0.002214 | -1.23361 | DCP2 | 0.588964 | 0.957979 | 2.53943 | 0.024566 | -3.40295 |
| NECAB3 | 0.928507 | 2.250599 | 3.791211 | 0.002216 | -1.23473 | C3orf62 | 0.940682 | -1.01937 | 2.539386 | 0.024568 | -3.40303 |
| SCML1 | 1.275248 | 0.370288 | 3.788494 | 0.002228 | -1.23943 | SLC9A3R2 | -0.96607 | 3.410762 | -2.53866 | 0.024602 | -3.40425 |
| TUBA4A | -1.92798 | 3.795637 | -3.78582 | 0.002239 | -1.24405 | BATF2 | -1.71864 | 0.370838 | -2.53818 | 0.024624 | -3.40505 |
| EPHA4 | 1.686255 | 2.338609 | 3.785362 | 0.002241 | -1.24484 | SMIM29 | -0.75004 | 4.963315 | -2.53793 | 0.024636 | -3.40548 |
| BDNF | 2.213513 | 1.014287 | 3.781278 | 0.002259 | -1.2519 | CACNB3 | -0.5537 | 3.735823 | -2.53745 | 0.024659 | -3.40629 |
| ADRA1B | 1.113896 | 0.983479 | 3.779823 | 0.002265 | -1.25442 | TLCD5 | 0.616882 | 1.40006 | 2.53716 | 0.024672 | -3.40677 |
| PABPC1L2B | -2.45966 | 0.03736 | -3.77831 | 0.002272 | -1.25703 | CNTN1 | -1.44482 | -0.18345 | -2.53518 | 0.024765 | -3.4101 |
| FRRS1L | -3.01686 | 0.826399 | -3.77801 | 0.002273 | -1.25756 | SPANXN4 | 0.561123 | -2.2652 | 2.53512 | 0.024768 | -3.4102 |
| EML4 | 0.600008 | 4.354177 | 3.776841 | 0.002278 | -1.25958 | LCK | -1.46091 | -0.37135 | -2.53492 | 0.024777 | -3.41054 |
| AOC2 | 0.954152 | -0.93922 | 3.776394 | 0.00228 | -1.26035 | UBE4A | 0.333989 | 4.146682 | 2.534317 | 0.024805 | -3.41155 |
| DPPA5 | 1.035187 | -1.77298 | 3.774702 | 0.002287 | -1.26327 | HSF2BP | 0.817827 | -0.95988 | 2.533716 | 0.024834 | -3.41256 |
| DNPH1 | -0.90998 | 4.946181 | -3.77272 | 0.002296 | -1.26671 | AKAP14 | 0.762245 | -1.63514 | 2.532547 | 0.024889 | -3.41452 |
| CATSPERZ | -2.79151 | 2.955223 | -3.76946 | 0.00231 | -1.27234 | FOXD2-AS1 | 1.716871 | -0.27501 | 2.532259 | 0.024902 | -3.415 |
| SYCE3 | -1.32197 | -0.44042 | -3.76806 | 0.002316 | -1.27477 | KIF24 | 1.090156 | -0.47241 | 2.530979 | 0.024963 | -3.41715 |
| ANKRD23 | 0.723588 | 0.331492 | 3.763143 | 0.002338 | -1.28327 | PGBD3 | 0.630806 | 0.699799 | 2.530836 | 0.02497 | -3.41739 |
| ADAP2 | -3.01197 | 2.712119 | -3.74963 | 0.002399 | -1.30665 | SOX15 | 0.598652 | -1.65345 | 2.530496 | 0.024986 | -3.41797 |
| TRIM62 | 0.864635 | -0.64698 | 3.746031 | 0.002416 | -1.31289 | GON4L | 0.444151 | 2.56376 | 2.530107 | 0.025004 | -3.41862 |
| FAM110C | -1.74991 | 0.634057 | -3.74286 | 0.002431 | -1.31839 | UBE2Q2 | -0.29053 | 3.632932 | -2.52966 | 0.025025 | -3.41936 |
| GNB4 | 0.871438 | 2.942494 | 3.741652 | 0.002436 | -1.32048 | DAXX | -0.52221 | 3.505683 | -2.52947 | 0.025034 | -3.41969 |
| id | logFC | AveExpr | t | P.Value | B | id | logFC | AveExpr | t | P.Value | B |
| KDM5B | 1.028168 | 3.513988 | 3.727245 | 0.002504 | -1.34543 | GADL1 | 0.566259 | -2.49415 | 2.528062 | 0.025101 | -3.42205 |
| IGSF22 | -1.37015 | -0.61227 | -3.72712 | 0.002505 | -1.34565 | OR1J1 | 0.788906 | -0.92272 | 2.527965 | 0.025106 | -3.42222 |
| VLDLR-AS1 | 0.894356 | -1.63027 | 3.725774 | 0.002511 | -1.34798 | PROC | -1.99227 | 0.399557 | -2.52699 | 0.025152 | -3.42385 |
| NRROS | -1.4091 | -0.24543 | -3.72447 | 0.002518 | -1.35025 | H2AC16 | 1.382791 | -0.0118 | 2.526675 | 0.025167 | -3.42438 |
| CCR4 | 0.923559 | -1.83727 | 3.724004 | 0.00252 | -1.35105 | POTEA | 0.585139 | -2.13857 | 2.526606 | 0.02517 | -3.4245 |
| ANKRD18DP | 1.715123 | -0.42768 | 3.723416 | 0.002523 | -1.35207 | DOLPP1 | 0.641692 | 2.455291 | 2.525632 | 0.025217 | -3.42613 |
| SPTBN1 | 0.875715 | -0.65966 | 3.71829 | 0.002548 | -1.36096 | CRNDE | 1.174309 | 2.312317 | 2.524921 | 0.025251 | -3.42733 |
| PNMT | -2.5889 | 0.336609 | -3.7175 | 0.002551 | -1.36232 | CADM3 | -2.2547 | -0.06884 | -2.52483 | 0.025255 | -3.42748 |
| HOXB8 | 2.787177 | -1.46213 | 3.716727 | 0.002555 | -1.36366 | TOP3A | 0.347108 | 1.450512 | 2.524223 | 0.025284 | -3.4285 |
| WT1-AS | -2.57787 | 2.771616 | -3.71586 | 0.002559 | -1.36517 | TUBBP5 | -1.81708 | -0.63643 | -2.52172 | 0.025404 | -3.43269 |
| CERS4 | -1.14992 | 1.879659 | -3.70994 | 0.002589 | -1.37543 | IGF2R | 0.844066 | 3.00192 | 2.52116 | 0.025431 | -3.43364 |
| AVPR1A | -3.09078 | 0.840141 | -3.70822 | 0.002597 | -1.37841 | OR6Q1 | 0.704909 | -1.5636 | 2.520764 | 0.02545 | -3.4343 |
| ZNF853 | -1.25054 | 0.917133 | -3.70724 | 0.002602 | -1.38011 | GCA | -1.73127 | 1.578956 | -2.52073 | 0.025452 | -3.43436 |
| TMEM131L | 1.005354 | 1.460632 | 3.704327 | 0.002617 | -1.38517 | SH3RF3 | 0.877996 | -0.17436 | 2.520626 | 0.025457 | -3.43453 |
| PRR29 | -1.87748 | -0.4336 | -3.70412 | 0.002618 | -1.38552 | GRAMD1B | 0.747348 | -0.16351 | 2.520117 | 0.025482 | -3.43539 |
| PTPRM | 1.184909 | 3.26199 | 3.698703 | 0.002645 | -1.39492 | PLEKHG7 | 0.388542 | -2.41861 | 2.520028 | 0.025486 | -3.43554 |
| CYP27A1 | -1.76344 | 3.359116 | -3.69837 | 0.002647 | -1.3955 | GRB10 | 0.610916 | 3.230204 | 2.519266 | 0.025523 | -3.43682 |
| MAP7 | -1.46505 | -0.32954 | -3.69671 | 0.002655 | -1.39837 | ZIC4 | 0.732958 | -0.24932 | 2.519229 | 0.025524 | -3.43688 |
| PFN2 | 1.119172 | 1.487145 | 3.696544 | 0.002656 | -1.39867 | ZNF20 | 0.46525 | 0.254072 | 2.51889 | 0.025541 | -3.43744 |
| SLC30A2 | -2.40876 | 0.187101 | -3.68315 | 0.002725 | -1.42192 | ZNF185 | -1.62347 | 1.481797 | -2.51843 | 0.025563 | -3.43822 |
| RGS11 | -2.11123 | 3.125669 | -3.67906 | 0.002746 | -1.42901 | IL36A | 0.668264 | -1.72554 | 2.518344 | 0.025567 | -3.43836 |
| PROM1 | -2.75657 | 0.495689 | -3.67762 | 0.002754 | -1.43151 | TNFSF11 | -1.35198 | -1.18478 | -2.51806 | 0.025581 | -3.43883 |
| CLDN3 | -2.87497 | 0.706117 | -3.67674 | 0.002759 | -1.43303 | SPRED2 | 0.877772 | 0.739853 | 2.517598 | 0.025603 | -3.43961 |
| LINC00632 | 0.83924 | -0.78917 | 3.675086 | 0.002767 | -1.43591 | IGF1 | -2.66995 | 1.562153 | -2.5166 | 0.025652 | -3.44128 |
| SLC6A12 | -1.96749 | -0.33418 | -3.67411 | 0.002772 | -1.43759 | HSPB9 | 0.595711 | -0.54236 | 2.516136 | 0.025674 | -3.44206 |
| id | logFC | AveExpr | t | P.Value | B | id | logFC | AveExpr | t | P.Value | B |
| ZNF608 | 1.852582 | -1.49256 | 3.66891 | 0.0028 | -1.44663 | ANXA13 | -1.46524 | -1.02134 | -2.51555 | 0.025703 | -3.44305 |
| LAMC1 | 1.405957 | 4.328876 | 3.66738 | 0.002808 | -1.44929 | MYADML | 0.605181 | -2.06176 | 2.515549 | 0.025703 | -3.44305 |
| DMRTC1 | -2.63228 | -0.76142 | -3.65841 | 0.002857 | -1.46487 | RBM15-AS1 | 0.707308 | -1.92066 | 2.515334 | 0.025713 | -3.44341 |
| ACVR1B | 0.851649 | -0.19909 | 3.656197 | 0.002869 | -1.46871 | KCND2 | -1.92013 | 0.561777 | -2.5153 | 0.025715 | -3.44347 |
| CASP3 | 0.751099 | 3.925104 | 3.648831 | 0.00291 | -1.48151 | TSPO2 | 0.61421 | 1.225309 | 2.514217 | 0.025768 | -3.44528 |
| RBP1 | -3.50525 | 4.181539 | -3.64268 | 0.002945 | -1.4922 | CNTN4 | -1.78393 | 0.811703 | -2.51406 | 0.025776 | -3.44555 |
| FAM3B | -1.52118 | -1.21523 | -3.63341 | 0.002997 | -1.50832 | PPP2R5A | 0.58356 | 0.916973 | 2.513741 | 0.025791 | -3.44608 |
| PLCE1-AS1 | 0.901517 | -2.21433 | 3.628801 | 0.003024 | -1.51632 | FBP1 | -1.66913 | -1.1223 | -2.51188 | 0.025882 | -3.4492 |
| PTGER3 | -1.9346 | -1.30106 | -3.61699 | 0.003093 | -1.53686 | CCDC134 | 0.668922 | 0.566684 | 2.510929 | 0.025929 | -3.45079 |
| MYH7 | 0.696819 | -2.06702 | 3.615442 | 0.003103 | -1.53956 | FAIM | -1.00064 | 2.600727 | -2.51069 | 0.02594 | -3.45118 |
| ZNF513 | 0.706395 | 2.771499 | 3.615014 | 0.003105 | -1.5403 | FER1L6 | 0.529514 | -2.15659 | 2.510523 | 0.025949 | -3.45147 |
| FRMD4A | 1.235323 | -0.47975 | 3.6083 | 0.003145 | -1.55198 | TMEM52 | -2.1776 | 1.415686 | -2.51049 | 0.02595 | -3.45153 |
| SPATA5 | 0.925149 | -1.77263 | 3.607595 | 0.00315 | -1.55321 | KLHL38 | 0.512784 | -0.55092 | 2.509342 | 0.026007 | -3.45345 |
| ZNF385A | -0.77537 | 2.693299 | -3.60645 | 0.003157 | -1.5552 | IFNK | 0.649905 | -2.38786 | 2.508916 | 0.026028 | -3.45416 |
| MSL3P1 | 1.062737 | 0.327647 | 3.601083 | 0.003189 | -1.56454 | LOX | 1.602158 | 3.836917 | 2.508839 | 0.026031 | -3.45429 |
| ZFPM2 | 1.720406 | 0.264728 | 3.600066 | 0.003195 | -1.56631 | PHLPP1 | 0.863126 | -0.49943 | 2.508026 | 0.026071 | -3.45566 |
| ABCC8 | -2.8034 | 1.867808 | -3.59783 | 0.003209 | -1.57019 | FBXO25 | -0.43942 | 3.90098 | -2.50719 | 0.026113 | -3.45706 |
| FOXD1 | 3.133214 | 1.70448 | 3.596364 | 0.003218 | -1.57275 | ANKZF1 | 0.583758 | 1.645729 | 2.506739 | 0.026135 | -3.45781 |
| ZNF112 | 0.556122 | 1.576086 | 3.595084 | 0.003226 | -1.57498 | IFITM4P | -1.39523 | 4.222393 | -2.50599 | 0.026172 | -3.45906 |
| LAD1 | -2.60119 | -0.74263 | -3.5942 | 0.003232 | -1.57652 | CRYL1 | -0.59569 | 3.236993 | -2.50564 | 0.026189 | -3.45965 |
| TENT4B | 1.114409 | -1.51538 | 3.590318 | 0.003256 | -1.58327 | IBA57 | 0.564808 | 1.80715 | 2.505416 | 0.0262 | -3.46003 |
| SOWAHD | -1.60161 | 1.358482 | -3.58911 | 0.003263 | -1.58537 | RINL | -1.17917 | 2.086933 | -2.50511 | 0.026216 | -3.46055 |
| CD74 | -2.766 | 0.745113 | -3.5875 | 0.003273 | -1.58818 | MKRN7P | -1.38607 | -1.65363 | -2.50449 | 0.026246 | -3.46158 |
| FBXO43 | 1.397453 | -0.42595 | 3.584525 | 0.003292 | -1.59336 | SRD5A2 | -1.47122 | -0.0742 | -2.50405 | 0.026268 | -3.46232 |
| ULBP3 | 1.329959 | -1.15739 | 3.583473 | 0.003299 | -1.59519 | RSPH1 | -1.08486 | -0.86086 | -2.50221 | 0.02636 | -3.46539 |
| id | logFC | AveExpr | t | P.Value | B | id | logFC | AveExpr | t | P.Value | B |
| MATK | -2.02731 | 0.759853 | -3.58271 | 0.003304 | -1.59652 | OTC | 0.503752 | -1.97669 | 2.501266 | 0.026407 | -3.46697 |
| PRAME | 3.926107 | 0.22716 | 3.579115 | 0.003327 | -1.60278 | CREB5 | 0.917321 | 0.760173 | 2.500425 | 0.026449 | -3.46838 |
| GTF2IRD1P1 | 1.054776 | -2.16988 | 3.577839 | 0.003335 | -1.605 | OIP5 | 1.333938 | 1.31052 | 2.500059 | 0.026467 | -3.46899 |
| TRAF4 | 0.979317 | 2.701218 | 3.576833 | 0.003341 | -1.60675 | SLITRK2 | 0.884825 | -1.85894 | 2.49845 | 0.026548 | -3.47169 |
| SERPINA9 | 1.648126 | -1.42256 | 3.574603 | 0.003356 | -1.61063 | PLPBP | -0.49773 | 2.856644 | -2.49809 | 0.026566 | -3.47228 |
| BLVRB | -0.72647 | 4.327074 | -3.57061 | 0.003381 | -1.61759 | C1orf56 | 0.522818 | 2.352685 | 2.497837 | 0.026579 | -3.47271 |
| FBN2 | 2.086915 | 2.671425 | 3.570532 | 0.003382 | -1.61772 | PCBP3 | -1.19764 | 0.232066 | -2.49779 | 0.026581 | -3.47279 |
| GASK1B | -2.23007 | 3.345645 | -3.56801 | 0.003398 | -1.62212 | CDC42SE1 | 0.680332 | 1.769448 | 2.49761 | 0.02659 | -3.47309 |
| SH3TC2 | 1.020383 | -2.1423 | 3.566711 | 0.003407 | -1.62438 | CDKN2C | 1.201105 | 2.168036 | 2.497028 | 0.026619 | -3.47407 |
| CRISP3 | 0.984154 | -2.09894 | 3.566296 | 0.00341 | -1.6251 | TOP2A | 1.541292 | 1.162754 | 2.496614 | 0.02664 | -3.47476 |
| NPR1 | -1.35115 | 2.501956 | -3.56462 | 0.003421 | -1.62802 | ZNF202 | 0.566821 | 0.439055 | 2.496009 | 0.026671 | -3.47577 |
| HRCT1 | 1.373082 | -0.13422 | 3.564395 | 0.003422 | -1.62841 | MXRA5 | 1.784875 | 2.233995 | 2.495691 | 0.026687 | -3.4763 |
| DISP2 | -1.48754 | 2.553042 | -3.55873 | 0.00346 | -1.63829 | PKIA | 0.957853 | 1.899782 | 2.495412 | 0.026701 | -3.47677 |
| DIO2 | -3.50697 | 3.123294 | -3.55818 | 0.003463 | -1.63923 | SGO2 | 1.022745 | 0.528486 | 2.494766 | 0.026733 | -3.47785 |
| SYN2 | -2.58199 | 1.100482 | -3.55607 | 0.003477 | -1.64291 | PTPRJ | 0.631936 | -2.10578 | 2.494033 | 0.02677 | -3.47908 |
| LINC01553 | 0.801864 | -2.13495 | 3.554904 | 0.003485 | -1.64495 | PLXNB2 | 0.678126 | 4.480365 | 2.493473 | 0.026799 | -3.48001 |
| NNAT | -1.76166 | 3.506093 | -3.5521 | 0.003504 | -1.64983 | MFAP4 | -1.47926 | 5.608046 | -2.49291 | 0.026827 | -3.48095 |
| CKMT1A | -2.89288 | 0.619947 | -3.5518 | 0.003506 | -1.65035 | PDIA4 | 0.406274 | 3.429503 | 2.491985 | 0.026874 | -3.4825 |
| SLC41A1 | 0.725256 | 3.014841 | 3.550287 | 0.003516 | -1.65299 | CARD9 | -1.46265 | 1.630728 | -2.49191 | 0.026878 | -3.48262 |
| TEKT2 | -1.87841 | 0.716967 | -3.54787 | 0.003533 | -1.6572 | EGFL6 | -1.64003 | 1.35772 | -2.49047 | 0.026951 | -3.48503 |
| HOXA4 | 1.415508 | 0.501874 | 3.546727 | 0.00354 | -1.65919 | PDE4DIP | 1.063114 | 1.220497 | 2.48981 | 0.026985 | -3.48614 |
| JCHAIN | 0.563235 | 0.213952 | 3.543485 | 0.003562 | -1.66484 | NIPSNAP3B | -0.95505 | -1.57815 | -2.48979 | 0.026986 | -3.48617 |
| COL5A1 | 1.486876 | 5.86207 | 3.542631 | 0.003568 | -1.66633 | CEP192 | 0.466793 | 1.982837 | 2.489139 | 0.027019 | -3.48726 |
| CDIP1 | -0.81862 | 3.078692 | -3.54221 | 0.003571 | -1.66707 | NCAM2 | 0.731109 | 1.153433 | 2.488424 | 0.027056 | -3.48845 |
| PI15 | -2.31156 | -0.22477 | -3.54027 | 0.003585 | -1.67045 | KCNG4 | 0.76648 | -0.6303 | 2.488209 | 0.027067 | -3.48881 |
| id | logFC | AveExpr | t | P.Value | B | id | logFC | AveExpr | t | P.Value | B |
| SLC4A7 | 1.013654 | 0.856849 | 3.537545 | 0.003603 | -1.6752 | AHCTF1 | 0.463751 | 1.64199 | 2.486892 | 0.027134 | -3.49101 |
| MACIR | 0.966963 | 1.857711 | 3.537082 | 0.003607 | -1.676 | KLHL7 | 0.715656 | 1.278762 | 2.486553 | 0.027151 | -3.49158 |
| GLS2 | -2.12203 | -0.84397 | -3.53694 | 0.003608 | -1.67624 | PDK4 | -1.14287 | 0.731512 | -2.4858 | 0.02719 | -3.49283 |
| PGM2L1 | -1.10029 | 2.256483 | -3.534 | 0.003628 | -1.68137 | ZNF157 | 0.716307 | -0.34463 | 2.48537 | 0.027212 | -3.49355 |
| FAM72A | 1.665051 | 0.006965 | 3.533671 | 0.00363 | -1.68195 | COX15 | 0.485638 | -0.58271 | 2.484988 | 0.027232 | -3.49419 |
| MYPN | 0.761612 | -1.99754 | 3.530611 | 0.003652 | -1.68728 | TYMSOS | 0.981855 | -0.80542 | 2.483016 | 0.027333 | -3.49749 |
| HOXC8 | 2.990852 | 0.239239 | 3.529292 | 0.003661 | -1.68958 | NUCKS1 | 0.518339 | 1.305937 | 2.482444 | 0.027363 | -3.49844 |
| FAM226A | -1.32204 | -0.25712 | -3.52784 | 0.003671 | -1.69211 | LARGE1 | 0.792943 | 3.521032 | 2.482397 | 0.027365 | -3.49852 |
| PKP4 | 0.880117 | 1.447487 | 3.526626 | 0.00368 | -1.69423 | OSTN | 0.319555 | -2.23244 | 2.482316 | 0.027369 | -3.49865 |
| EMC1 | 0.894948 | 2.464123 | 3.523613 | 0.003701 | -1.69948 | TIMELESS | 0.681467 | 3.18781 | 2.481702 | 0.027401 | -3.49968 |
| FANCB | 0.970277 | 0.322322 | 3.522003 | 0.003713 | -1.70229 | COL12A1 | 1.428332 | 5.067773 | 2.481199 | 0.027427 | -3.50052 |
| CTIF | 0.660679 | 3.583023 | 3.520526 | 0.003723 | -1.70486 | DDX31 | 0.487647 | 1.306043 | 2.47983 | 0.027498 | -3.50281 |
| JUP | -1.32166 | 4.047509 | -3.52023 | 0.003725 | -1.70538 | PNCK | -1.73137 | -0.16369 | -2.47975 | 0.027502 | -3.50293 |
| SLC6A4 | -1.00415 | -0.86724 | -3.5182 | 0.00374 | -1.70892 | RAB27A | -1.10195 | 0.358395 | -2.47952 | 0.027514 | -3.50332 |
| BNIPL | 0.870198 | -2.31744 | 3.515923 | 0.003756 | -1.71289 | CHD7 | 1.094533 | 0.283191 | 2.479286 | 0.027526 | -3.50371 |
| OR52R1 | 0.949317 | -1.60255 | 3.515252 | 0.003761 | -1.71406 | NRN1 | 0.881711 | -1.0458 | 2.4785 | 0.027567 | -3.50503 |
| NFIB | 1.151696 | 1.575357 | 3.512022 | 0.003785 | -1.71969 | SPAST | 0.527086 | 0.517558 | 2.47847 | 0.027569 | -3.50508 |
| CHGA | -2.98974 | -0.48214 | -3.50935 | 0.003804 | -1.72436 | SEMA3B | -1.15606 | 4.957712 | -2.47758 | 0.027615 | -3.50656 |
| PSMG3-AS1 | 0.783347 | 1.28702 | 3.508679 | 0.003809 | -1.72552 | G2E3 | 0.506441 | 0.729828 | 2.477393 | 0.027625 | -3.50687 |
| SH2D3C | -3.07387 | 0.701161 | -3.50754 | 0.003817 | -1.7275 | HOXC13 | 2.092419 | -0.5393 | 2.477282 | 0.027631 | -3.50706 |
| DLX1 | 3.618334 | -0.2225 | 3.505995 | 0.003829 | -1.7302 | SPDYE2 | 0.495592 | 0.763618 | 2.475871 | 0.027704 | -3.50941 |
| PELI3 | -0.9281 | 3.43979 | -3.50347 | 0.003847 | -1.7346 | HSP90B1 | 0.369247 | 6.502531 | 2.475811 | 0.027708 | -3.50951 |
| MOV10 | 0.961572 | 2.021322 | 3.499268 | 0.003879 | -1.74194 | CYTH3 | 0.622647 | 4.70798 | 2.473558 | 0.027826 | -3.51327 |
| CKMT2 | -2.29197 | -0.03172 | -3.49704 | 0.003895 | -1.74582 | XRCC3 | 0.733248 | 2.425832 | 2.473285 | 0.02784 | -3.51373 |
| FAM117A | -0.71199 | 2.060585 | -3.49517 | 0.003909 | -1.74908 | SCAMP5 | -1.03554 | 1.994325 | -2.47264 | 0.027874 | -3.51481 |
| id | logFC | AveExpr | t | P.Value | B | id | logFC | AveExpr | t | P.Value | B |
| EME1 | 1.065038 | 0.417428 | 3.489985 | 0.003949 | -1.75813 | CASP10 | 0.600627 | -1.92066 | 2.472581 | 0.027877 | -3.5149 |
| DMKN | -2.89736 | 2.944098 | -3.48913 | 0.003955 | -1.75963 | SH3GL1P2 | 0.487862 | 0.166039 | 2.472278 | 0.027893 | -3.51541 |
| SLC7A8 | -1.62587 | -0.11568 | -3.48533 | 0.003984 | -1.76625 | ANKRD29 | -0.98574 | 1.055874 | -2.47157 | 0.02793 | -3.51659 |
| EYA2 | -2.24091 | 0.06027 | -3.48482 | 0.003988 | -1.76715 | DCLRE1C | 0.337709 | 1.746737 | 2.471045 | 0.027958 | -3.51746 |
| STAT5A | -0.97538 | 0.864209 | -3.48383 | 0.003996 | -1.76886 | HMGA2 | 1.789215 | 1.17774 | 2.470087 | 0.028008 | -3.51906 |
| PHYH | -0.69819 | 1.754674 | -3.48207 | 0.004009 | -1.77193 | CRYGD | 0.585285 | -0.95183 | 2.469892 | 0.028019 | -3.51938 |
| HOXB13 | 1.935413 | -1.79981 | 3.480642 | 0.00402 | -1.77443 | CEACAM1 | -1.13247 | -0.34119 | -2.46858 | 0.028088 | -3.52158 |
| BAZ1A | 1.041006 | 2.104754 | 3.480603 | 0.004021 | -1.7745 | CD300LB | 0.448272 | 0.602005 | 2.46695 | 0.028175 | -3.52429 |
| NDOR1 | 0.841696 | 3.56048 | 3.474919 | 0.004065 | -1.78442 | AGBL5 | 0.761347 | 0.860934 | 2.46626 | 0.028211 | -3.52544 |
| SUSD3 | -2.1775 | 3.492062 | -3.47435 | 0.004069 | -1.78541 | ILF3 | 0.56783 | 1.524578 | 2.466155 | 0.028217 | -3.52561 |
| HOTAIR | 3.011625 | -0.15826 | 3.472906 | 0.004081 | -1.78793 | PRTFDC1 | -0.97554 | 1.089498 | -2.46604 | 0.028223 | -3.5258 |
| OSBPL6 | 1.573094 | 1.939008 | 3.469442 | 0.004108 | -1.79398 | LRRN4CL | -1.9617 | 1.926182 | -2.46541 | 0.028256 | -3.52685 |
| PAPLN | -1.7242 | 1.749278 | -3.46924 | 0.00411 | -1.79434 | CHP1 | -0.41563 | 4.655695 | -2.46438 | 0.028312 | -3.52858 |
| IRF5 | -1.60697 | 2.314268 | -3.46754 | 0.004123 | -1.79729 | VANGL1 | 0.579066 | 3.264453 | 2.463947 | 0.028335 | -3.52929 |
| FOXC1 | 1.257378 | 0.984767 | 3.46498 | 0.004143 | -1.80177 | PMPCA | 0.485415 | 4.025491 | 2.463554 | 0.028356 | -3.52995 |
| BEX5 | -2.08729 | -0.62399 | -3.4632 | 0.004158 | -1.80488 | C2CD6 | 0.982651 | -0.04398 | 2.461924 | 0.028443 | -3.53266 |
| IRGQ | 0.753306 | 0.380576 | 3.462855 | 0.00416 | -1.80548 | ZNF536 | 2.594982 | -0.92774 | 2.461809 | 0.028449 | -3.53286 |
| CXCL16 | -1.53206 | 1.182174 | -3.46038 | 0.00418 | -1.80979 | NRAP | 1.834373 | -1.36948 | 2.46116 | 0.028484 | -3.53394 |
| DACT3 | -0.96043 | 3.382802 | -3.46 | 0.004183 | -1.81046 | PMS2P9 | 0.422879 | 0.857579 | 2.460962 | 0.028495 | -3.53427 |
| ARHGAP26 | 1.33015 | 0.678201 | 3.459853 | 0.004185 | -1.81072 | GDPD1 | -0.67619 | -2.06713 | -2.46002 | 0.028545 | -3.53584 |
| ZNF681 | 0.678129 | -1.88258 | 3.458144 | 0.004198 | -1.8137 | SRRT | 0.545543 | 1.520769 | 2.458902 | 0.028606 | -3.5377 |
| DTX1 | -2.42032 | 1.358308 | -3.45759 | 0.004203 | -1.81466 | TMEM47 | 1.262254 | 0.946021 | 2.458827 | 0.02861 | -3.53782 |
| ESRP1 | -2.14819 | -0.17634 | -3.45649 | 0.004212 | -1.81659 | SRCIN1 | -1.64475 | -0.96908 | -2.45822 | 0.028642 | -3.53883 |
| TMEM121B | -1.42958 | 0.941203 | -3.4546 | 0.004227 | -1.81989 | GBA | 0.787875 | 4.117459 | 2.458173 | 0.028645 | -3.53891 |
| PGR | -2.90718 | 1.545819 | -3.45316 | 0.004239 | -1.82239 | SLC22A9 | 0.487004 | -2.30867 | 2.456692 | 0.028725 | -3.54138 |
| id | logFC | AveExpr | t | P.Value | B | id | logFC | AveExpr | t | P.Value | B |
| HIVEP1 | 0.829777 | 1.739759 | 3.451745 | 0.00425 | -1.82487 | SREBF2 | 0.637286 | 3.389307 | 2.45625 | 0.028749 | -3.54211 |
| HOXA3 | 1.869736 | 1.386579 | 3.449515 | 0.004269 | -1.82876 | TMEM64 | 0.525175 | 0.072722 | 2.456067 | 0.028759 | -3.54241 |
| EPCAM | -3.22753 | 0.706901 | -3.44887 | 0.004274 | -1.82989 | BLOC1S6 | -0.33183 | 3.69965 | -2.45508 | 0.028812 | -3.54406 |
| DNALI1 | -1.79258 | 0.588252 | -3.44669 | 0.004292 | -1.8337 | BAALC | -1.69877 | 0.00113 | -2.45467 | 0.028835 | -3.54474 |
| COL4A4 | 1.648617 | 1.922792 | 3.445591 | 0.004301 | -1.83561 | CDH6 | 1.372946 | 1.450345 | 2.454666 | 0.028835 | -3.54475 |
| TLCD1 | 0.850024 | 1.661667 | 3.44372 | 0.004317 | -1.83888 | TNFRSF10A | -1.42435 | 0.287924 | -2.4542 | 0.02886 | -3.54553 |
| IPO9 | 1.456688 | 0.328709 | 3.441866 | 0.004332 | -1.84212 | C19orf57 | 0.992606 | 0.556305 | 2.454042 | 0.028869 | -3.54578 |
| GLI2 | 0.940539 | 1.957352 | 3.440223 | 0.004346 | -1.84498 | PTH | 0.572422 | -2.45083 | 2.453834 | 0.02888 | -3.54613 |
| CPAMD8 | -2.29076 | 2.589414 | -3.43664 | 0.004376 | -1.85125 | RAD54L | 1.263523 | 1.976401 | 2.453408 | 0.028903 | -3.54684 |
| CATSPERB | -1.30121 | -1.67345 | -3.4359 | 0.004382 | -1.85253 | LINC00602 | 0.66284 | -1.96913 | 2.452939 | 0.028929 | -3.54762 |
| PAOX | -0.71458 | 3.083022 | -3.43552 | 0.004385 | -1.8532 | EPM2AIP1 | 0.950189 | 0.276729 | 2.451794 | 0.028991 | -3.54952 |
| CD14 | -2.68191 | 3.376665 | -3.43114 | 0.004423 | -1.86084 | HECW2 | 0.875133 | -2.07103 | 2.451791 | 0.028991 | -3.54953 |
| HOXA2 | 2.218791 | 0.673701 | 3.429842 | 0.004434 | -1.86311 | USP51 | 1.373249 | -1.18086 | 2.451148 | 0.029027 | -3.5506 |
| THNSL2 | -1.81253 | 2.153859 | -3.4293 | 0.004438 | -1.86406 | GDNF | 1.38555 | -0.10685 | 2.450487 | 0.029063 | -3.5517 |
| RANBP17 | 0.657022 | 1.982586 | 3.427464 | 0.004454 | -1.86726 | NAV2-AS4 | 0.823998 | -1.40364 | 2.44979 | 0.029101 | -3.55286 |
| IFITM1 | -2.30142 | 5.154012 | -3.42297 | 0.004493 | -1.87511 | MAP3K15 | -0.93714 | -2.01414 | -2.44964 | 0.029109 | -3.5531 |
| ARHGAP10 | 0.875281 | 1.764208 | 3.421301 | 0.004507 | -1.87803 | PKD1L3 | 0.511572 | -2.17444 | 2.448834 | 0.029153 | -3.55445 |
| ABCA3 | -1.57416 | 2.863124 | -3.41869 | 0.00453 | -1.88258 | CLDN15 | 0.72451 | 0.91938 | 2.448278 | 0.029184 | -3.55537 |
| MT1F | -3.27629 | 1.56695 | -3.41707 | 0.004544 | -1.88542 | EEF1AKMT4-ECE2 | -1.97522 | -0.05868 | -2.44824 | 0.029186 | -3.55544 |
| DQX1 | 1.939934 | -1.23225 | 3.415312 | 0.00456 | -1.88849 | FAM20C | 0.579246 | 4.918811 | 2.448001 | 0.029199 | -3.55583 |
| C21orf58 | 0.709068 | 1.314076 | 3.41524 | 0.00456 | -1.88861 | NKAIN4 | -2.23125 | 2.212595 | -2.44783 | 0.029208 | -3.55611 |
| FLG | 1.076253 | -1.49257 | 3.410078 | 0.004606 | -1.89763 | GPR87 | 0.586887 | -1.90573 | 2.446975 | 0.029256 | -3.55754 |
| XXYLT1 | 0.836195 | 2.806155 | 3.410052 | 0.004606 | -1.89767 | GRIN1 | -1.08275 | -1.78462 | -2.4466 | 0.029276 | -3.55816 |
| SHROOM1 | -1.06552 | 1.22224 | -3.40646 | 0.004638 | -1.90394 | CD38 | -1.79177 | -0.43931 | -2.44359 | 0.029443 | -3.56317 |
| id | logFC | AveExpr | t | P.Value | B | id | logFC | AveExpr | t | P.Value | B |
| LOC100240734 | 1.457088 | -1.27458 | 3.405439 | 0.004647 | -1.90573 | GALNT18 | 0.671984 | 4.174764 | 2.443487 | 0.029448 | -3.56333 |
| FOXF1 | 1.705278 | 0.823464 | 3.40098 | 0.004687 | -1.91352 | E2F8 | 1.513049 | -0.40578 | 2.442209 | 0.029519 | -3.56546 |
| IFITM2 | -1.51555 | 5.595633 | -3.40042 | 0.004692 | -1.9145 | PRICKLE2 | -0.71704 | 1.67791 | -2.4414 | 0.029565 | -3.56681 |
| NGF | 1.164125 | 0.123666 | 3.398172 | 0.004713 | -1.91843 | ARRDC2 | -0.52291 | 2.090287 | -2.44139 | 0.029565 | -3.56682 |
| EGFL7 | -1.14997 | 3.749644 | -3.396 | 0.004733 | -1.92223 | ELOA | 0.342452 | 1.859082 | 2.441094 | 0.029581 | -3.56731 |
| PXDN | 1.51259 | 5.104322 | 3.395512 | 0.004737 | -1.92307 | PRR22 | 1.081513 | 0.144972 | 2.440987 | 0.029587 | -3.56749 |
| SYT13 | -3.3162 | 1.157786 | -3.39417 | 0.004749 | -1.92541 | OR5K4 | 0.64382 | -2.46436 | 2.440908 | 0.029592 | -3.56762 |
| PTPRK | 0.871875 | 3.527586 | 3.394049 | 0.00475 | -1.92563 | FAM238A | 0.527865 | -2.25854 | 2.440546 | 0.029612 | -3.56822 |
| PPIC | 0.689764 | 3.258298 | 3.393878 | 0.004752 | -1.92593 | KDM4A | 0.541745 | 2.290839 | 2.440464 | 0.029616 | -3.56836 |
| MGC70870 | -1.73821 | -0.59095 | -3.39275 | 0.004762 | -1.9279 | LINC01801 | 0.663727 | -1.79178 | 2.439335 | 0.029679 | -3.57023 |
| MARCHF3 | 1.425671 | 0.649898 | 3.389845 | 0.004789 | -1.93297 | DCDC2B | 0.675909 | -2.13348 | 2.439207 | 0.029687 | -3.57044 |
| ZNF100 | 0.633749 | 1.102947 | 3.386805 | 0.004817 | -1.93828 | IRX5 | 2.11157 | 0.832008 | 2.439067 | 0.029694 | -3.57068 |
| LAIR2 | 0.849583 | -2.02354 | 3.38557 | 0.004829 | -1.94044 | NUP188 | 0.969926 | 2.199663 | 2.438652 | 0.029718 | -3.57137 |
| TCN1 | 0.666992 | -1.9359 | 3.385145 | 0.004833 | -1.94118 | MYEF2 | 1.238586 | -0.92962 | 2.438386 | 0.029733 | -3.57181 |
| CPXM1 | -2.01276 | 3.167391 | -3.38465 | 0.004837 | -1.94205 | RAI1 | 0.614492 | 2.911786 | 2.438032 | 0.029752 | -3.5724 |
| P3H1 | 1.061177 | 3.566092 | 3.38314 | 0.004851 | -1.94469 | TSKU | -0.92857 | 3.179589 | -2.43788 | 0.029761 | -3.57265 |
| TMEM117 | 0.959935 | 1.918953 | 3.380941 | 0.004872 | -1.94853 | DRC7 | 0.707169 | -1.38146 | 2.437834 | 0.029763 | -3.57272 |
| JADE1 | 1.09012 | 1.012033 | 3.379348 | 0.004887 | -1.95131 | MMEL1 | -0.96992 | -0.60319 | -2.43705 | 0.029807 | -3.57402 |
| PRKCE | 1.025667 | 0.377796 | 3.378452 | 0.004895 | -1.95288 | CLDN17 | -0.42059 | -2.04242 | -2.43702 | 0.029809 | -3.57407 |
| ATP11C | 0.832148 | 1.894661 | 3.378305 | 0.004897 | -1.95313 | KLHL21 | 0.52255 | 4.310788 | 2.436888 | 0.029816 | -3.5743 |
| ABCC6 | -0.94131 | 0.497841 | -3.37818 | 0.004898 | -1.95336 | XYLT1 | 0.970099 | 1.156184 | 2.435793 | 0.029878 | -3.57611 |
| ADAMTSL1 | 1.958564 | 1.125204 | 3.375769 | 0.004921 | -1.95756 | TTTY8 | 0.70748 | -2.18825 | 2.435525 | 0.029893 | -3.57656 |
| KCNS2 | -1.81381 | -1.00928 | -3.37522 | 0.004926 | -1.95853 | RGS9 | -1.53729 | 0.445625 | -2.43457 | 0.029947 | -3.57813 |
| HOXC6 | 2.193492 | 3.057554 | 3.372581 | 0.004951 | -1.96313 | SPATC1L | 1.194971 | 1.602524 | 2.433736 | 0.029994 | -3.57953 |
| id | logFC | AveExpr | t | P.Value | B | id | logFC | AveExpr | t | P.Value | B |
| COL4A1 | 1.419746 | 5.388096 | 3.367654 | 0.004998 | -1.97174 | MPV17L | -0.85446 | 0.180199 | -2.43326 | 0.030021 | -3.58032 |
| USP13 | 0.924485 | 1.777801 | 3.364833 | 0.005026 | -1.97667 | PUS7 | 0.609267 | 2.713284 | 2.430876 | 0.030156 | -3.58427 |
| FKBP2 | -0.47592 | 6.242318 | -3.36005 | 0.005072 | -1.98504 | EPHA5-AS1 | 1.280733 | -1.15905 | 2.430431 | 0.030181 | -3.58501 |
| HECW1 | 1.525737 | -1.2923 | 3.357784 | 0.005095 | -1.98899 | MFAP3 | 0.556586 | 1.273117 | 2.429873 | 0.030213 | -3.58593 |
| MAPK8IP2 | -1.64449 | 1.098593 | -3.35699 | 0.005102 | -1.99037 | SFT2D3 | 0.548584 | 1.132333 | 2.429763 | 0.030219 | -3.58612 |
| AK9 | -1.12193 | -1.61883 | -3.35637 | 0.005109 | -1.99146 | FSHR | 0.600204 | -1.65827 | 2.428821 | 0.030272 | -3.58768 |
| PHF11 | -1.20168 | 1.935975 | -3.35314 | 0.00514 | -1.9971 | SLC13A2 | 0.772881 | -0.17099 | 2.428467 | 0.030293 | -3.58827 |
| TP53BP2 | 1.075952 | 1.784782 | 3.349112 | 0.005181 | -2.00414 | RORB | -1.71322 | 2.537557 | -2.4282 | 0.030308 | -3.58871 |
| GIT1 | 0.514998 | 0.593072 | 3.348892 | 0.005183 | -2.00453 | OGFRP1 | 0.472686 | -1.78094 | 2.428046 | 0.030317 | -3.58896 |
| NLGN4X | -2.30602 | 2.66296 | -3.34791 | 0.005193 | -2.00625 | PRKAR1B | -0.8236 | 1.685311 | -2.42597 | 0.030435 | -3.59241 |
| TRIM47 | -0.86027 | 4.01711 | -3.3474 | 0.005198 | -2.00713 | GALNT5 | 1.841619 | -0.4674 | 2.425783 | 0.030446 | -3.59272 |
| SOX7 | -1.30308 | -1.57835 | -3.34551 | 0.005217 | -2.01044 | PLAC8L1 | 0.953114 | 0.277029 | 2.425743 | 0.030448 | -3.59278 |
| TSPAN15 | -1.65764 | 0.345861 | -3.34177 | 0.005254 | -2.01698 | CDH20 | 1.802752 | -1.42132 | 2.425201 | 0.030479 | -3.59368 |
| ADSS1 | -0.85997 | 1.890312 | -3.33927 | 0.00528 | -2.02135 | ARR3 | 0.794535 | -1.32874 | 2.424958 | 0.030493 | -3.59408 |
| IFNLR1 | -1.48106 | -0.77512 | -3.3343 | 0.005331 | -2.03003 | NRF1 | 0.421595 | -0.16407 | 2.424494 | 0.03052 | -3.59485 |
| GPR89B | 0.629713 | 1.788897 | 3.332744 | 0.005347 | -2.03275 | ZNF555 | -0.60437 | 1.589686 | -2.42431 | 0.030531 | -3.59516 |
| ABHD18 | 0.670549 | 0.155089 | 3.33097 | 0.005365 | -2.03585 | PRRG4 | -1.03588 | -0.49146 | -2.42426 | 0.030533 | -3.59523 |
| ISG20 | -1.10641 | 2.800794 | -3.32962 | 0.005379 | -2.0382 | TMEM178B | 1.431136 | 0.658807 | 2.423379 | 0.030584 | -3.5967 |
| LINC02389 | 0.695722 | -1.5737 | 3.328704 | 0.005389 | -2.03981 | DNTT | 0.505266 | -2.39008 | 2.422876 | 0.030613 | -3.59753 |
| HAPLN4 | -1.38879 | 0.103355 | -3.3256 | 0.005421 | -2.04523 | ZNF850 | 0.838093 | -1.21606 | 2.422628 | 0.030627 | -3.59795 |
| FOXP1 | 1.037505 | 1.960629 | 3.322903 | 0.005449 | -2.04994 | TRAF3IP3 | -1.95272 | -0.48775 | -2.42186 | 0.030671 | -3.59922 |
| C18orf32 | 0.784223 | -1.69553 | 3.318292 | 0.005498 | -2.058 | MLH1 | 0.330425 | 4.419958 | 2.421447 | 0.030695 | -3.5999 |
| TLE3 | 1.262755 | 0.108287 | 3.315231 | 0.005531 | -2.06335 | REXO5 | 0.827037 | -0.43395 | 2.420562 | 0.030746 | -3.60137 |
| TMEM71 | -0.74492 | -1.54006 | -3.31403 | 0.005543 | -2.06545 | CA11 | -1.19499 | 2.250097 | -2.41853 | 0.030864 | -3.60474 |
| C1orf198 | 1.209476 | 5.817956 | 3.311596 | 0.00557 | -2.06971 | OOEP | 0.734927 | -2.11469 | 2.418287 | 0.030878 | -3.60514 |
| id | logFC | AveExpr | t | P.Value | B | id | logFC | AveExpr | t | P.Value | B |
| KCTD14 | -1.66588 | -0.17298 | -3.31102 | 0.005576 | -2.07072 | GRIN2C | -2.34732 | 2.608367 | -2.41814 | 0.030886 | -3.60538 |
| ACSS1 | -1.1303 | 1.99003 | -3.31089 | 0.005577 | -2.07094 | ERCC4 | 0.524674 | -0.23396 | 2.416884 | 0.030959 | -3.60746 |
| TMEM86A | -1.21656 | -0.939 | -3.30652 | 0.005624 | -2.07858 | ZFR2 | -1.39229 | -1.30735 | -2.41677 | 0.030966 | -3.60765 |
| NBAS | 0.818179 | 3.443051 | 3.304056 | 0.005651 | -2.08288 | FST | 2.153972 | 1.872246 | 2.416037 | 0.031009 | -3.60886 |
| CES5A | 0.534622 | -2.29258 | 3.302355 | 0.00567 | -2.08586 | KAAG1 | 0.620875 | -2.14396 | 2.415695 | 0.031029 | -3.60943 |
| RFX5 | 0.774792 | 2.258433 | 3.301261 | 0.005682 | -2.08777 | DOCK8 | -1.46028 | -0.32851 | -2.41511 | 0.031063 | -3.6104 |
| QRICH2 | 0.859054 | 0.232087 | 3.300683 | 0.005688 | -2.08878 | SMC6 | 0.498436 | 2.475887 | 2.41473 | 0.031085 | -3.61102 |
| C19orf54 | 0.71335 | -0.00829 | 3.299834 | 0.005697 | -2.09026 | LINC00643 | 0.849216 | -1.88481 | 2.414033 | 0.031126 | -3.61218 |
| RAB39B | -2.14261 | -0.3241 | -3.2998 | 0.005698 | -2.09032 | SNCAIP | -1.47745 | 2.12256 | -2.41386 | 0.031136 | -3.61247 |
| PLXNA2 | 1.818358 | -1.0888 | 3.296901 | 0.00573 | -2.09539 | WNT2 | -2.23041 | -0.02399 | -2.41352 | 0.031156 | -3.61303 |
| CHMP1B | -0.57348 | 2.354173 | -3.29443 | 0.005757 | -2.0997 | UBXN1 | -0.43913 | 5.741486 | -2.41313 | 0.031178 | -3.61367 |
| TOX | -1.00068 | -1.91996 | -3.28913 | 0.005816 | -2.10896 | LBR | 0.743765 | 4.34321 | 2.411693 | 0.031263 | -3.61605 |
| ETV5 | 1.042384 | 0.467143 | 3.288722 | 0.005821 | -2.10968 | CLEC2L | -1.40302 | -0.58885 | -2.41098 | 0.031305 | -3.61723 |
| DBNDD2 | -1.00754 | 3.04432 | -3.28646 | 0.005847 | -2.11363 | SOX4 | -0.74352 | 2.214723 | -2.41098 | 0.031305 | -3.61723 |
| AATK | -1.18994 | -0.66802 | -3.28636 | 0.005848 | -2.11381 | EPPIN | 0.419769 | -2.38614 | 2.410813 | 0.031314 | -3.6175 |
| ARMH1 | 1.019233 | -0.04599 | 3.278708 | 0.005935 | -2.12718 | NOXA1 | -1.07665 | 2.360176 | -2.41045 | 0.031336 | -3.6181 |
| MFSD6 | -1.15268 | 1.981928 | -3.27741 | 0.00595 | -2.12945 | CHMP4B | -0.48017 | 2.954882 | -2.41015 | 0.031353 | -3.6186 |
| NMUR1 | -2.40681 | -0.2491 | -3.2771 | 0.005953 | -2.12999 | CLIC5 | -0.81131 | 3.080729 | -2.41013 | 0.031355 | -3.61864 |
| IMMP2L | -1.20669 | 2.094016 | -3.2767 | 0.005958 | -2.13069 | GUCY1A1 | -2.23687 | 1.521988 | -2.40977 | 0.031376 | -3.61923 |
| AASS | 1.053701 | 2.104062 | 3.272435 | 0.006007 | -2.13814 | SEC16A | 0.460872 | 2.921785 | 2.409361 | 0.0314 | -3.61991 |
| DDX11 | 0.721562 | 2.655893 | 3.272068 | 0.006011 | -2.13878 | HOXC9 | 2.172761 | 2.258678 | 2.408978 | 0.031423 | -3.62054 |
| MDN1 | 0.696164 | 0.268582 | 3.270366 | 0.006031 | -2.14176 | CMTM3 | -0.59977 | 4.817421 | -2.40768 | 0.031499 | -3.62269 |
| EXOC3L4 | -1.82362 | 0.55924 | -3.26979 | 0.006038 | -2.14277 | CD244 | -0.5792 | -2.35816 | -2.40661 | 0.031562 | -3.62445 |
| IRF2BPL | 0.820386 | 1.838104 | 3.26951 | 0.006041 | -2.14325 | SLC35G5 | 0.667328 | 0.296156 | 2.406115 | 0.031592 | -3.62527 |
| LINC00634 | 0.573016 | -1.3915 | 3.268254 | 0.006056 | -2.14545 | TH | -1.07666 | 1.096619 | -2.40574 | 0.031614 | -3.62589 |
| id | logFC | AveExpr | t | P.Value | B | id | logFC | AveExpr | t | P.Value | B |
| COL9A2 | -2.36899 | 2.521496 | -3.26456 | 0.006099 | -2.1519 | RPL39L | 0.853714 | 1.780438 | 2.404639 | 0.03168 | -3.62771 |
| RASAL3 | -2.50572 | 1.652256 | -3.26388 | 0.006107 | -2.15309 | MSTO1 | 0.43942 | 3.180454 | 2.403558 | 0.031744 | -3.6295 |
| PAMR1 | -2.58716 | 4.351417 | -3.2627 | 0.006121 | -2.15515 | CDCA4 | 1.166002 | -0.78729 | 2.403498 | 0.031747 | -3.6296 |
| RTP4 | -2.36247 | 0.987409 | -3.26155 | 0.006135 | -2.15717 | H3C11 | 1.808953 | 0.065662 | 2.402833 | 0.031787 | -3.6307 |
| SPEN | 0.633049 | 1.433094 | 3.261078 | 0.00614 | -2.15799 | AMN | -0.5632 | 2.918073 | -2.40277 | 0.031791 | -3.6308 |
| PGGHG | -0.80757 | 2.84132 | -3.25807 | 0.006176 | -2.16324 | BARHL2 | 0.912108 | -1.05359 | 2.402383 | 0.031814 | -3.63144 |
| SLA | -2.84323 | 1.166433 | -3.25791 | 0.006178 | -2.16352 | FCHO1 | -1.21254 | 2.346065 | -2.40165 | 0.031858 | -3.63265 |
| TMEM150C | -1.59599 | 1.095253 | -3.25688 | 0.00619 | -2.16533 | LINC02593 | 1.255533 | 1.198882 | 2.40138 | 0.031874 | -3.6331 |
| CYP2S1 | -1.8576 | 0.295954 | -3.25075 | 0.006264 | -2.17603 | GSAP | -1.44127 | 0.418481 | -2.4011 | 0.031891 | -3.63357 |
| SELENOV | -2.33238 | -0.53846 | -3.24921 | 0.006283 | -2.17872 | TMCC1 | -0.40238 | 2.659005 | -2.40023 | 0.031943 | -3.635 |
| MGAT4FP | 0.813987 | -2.02279 | 3.248045 | 0.006297 | -2.18076 | CSN1S1 | 0.551633 | -0.83778 | 2.40014 | 0.031948 | -3.63515 |
| SULT2B1 | -0.99243 | -1.80687 | -3.24661 | 0.006314 | -2.18327 | C5orf38 | 1.205825 | -0.3046 | 2.398756 | 0.032031 | -3.63743 |
| PLA2G4F | 0.475133 | -0.79483 | 3.245759 | 0.006325 | -2.18476 | ZNF770 | -0.35078 | 2.116306 | -2.39871 | 0.032034 | -3.63751 |
| HHEX | -1.25571 | -1.29764 | -3.2449 | 0.006335 | -2.18625 | LRRC4B | -1.49394 | 1.673425 | -2.39848 | 0.032048 | -3.63789 |
| FAM234B | 0.853992 | 0.414178 | 3.243995 | 0.006346 | -2.18784 | ARHGEF2 | 0.479225 | 1.65902 | 2.39841 | 0.032052 | -3.638 |
| CLEC2B | -2.43198 | 0.684007 | -3.24012 | 0.006394 | -2.19461 | PHC2 | -0.40355 | 4.3492 | -2.39838 | 0.032054 | -3.63806 |
| ENOX1 | 1.196428 | 0.743681 | 3.238781 | 0.00641 | -2.19695 | DEDD | 0.439705 | 1.99831 | 2.396213 | 0.032184 | -3.64163 |
| NUTM2A | 1.418989 | 0.034038 | 3.238031 | 0.00642 | -2.19826 | FKBP4 | -0.61814 | 2.870241 | -2.39447 | 0.03229 | -3.64451 |
| DTX3L | -1.1106 | 1.366655 | -3.23718 | 0.00643 | -2.19975 | FBXO27 | -1.07231 | -0.04224 | -2.39385 | 0.032327 | -3.64552 |
| C19orf71 | 0.950099 | -1.296 | 3.236965 | 0.006433 | -2.20012 | MAGEA4 | 0.607177 | -2.44557 | 2.393814 | 0.03233 | -3.64559 |
| MELTF | -1.76771 | 0.355227 | -3.23428 | 0.006466 | -2.20481 | MLKL | -1.67535 | 2.024459 | -2.3932 | 0.032366 | -3.6466 |
| PGAP1 | 0.733497 | 0.217528 | 3.234225 | 0.006467 | -2.20491 | CIDEB | -1.03862 | 0.541316 | -2.39278 | 0.032392 | -3.6473 |
| GFAP | 0.621673 | -2.11825 | 3.23394 | 0.006471 | -2.20541 | TMPRSS11B | 0.498316 | -2.28491 | 2.392516 | 0.032408 | -3.64773 |
| PCA3 | 0.627257 | -1.4328 | 3.231866 | 0.006497 | -2.20903 | LCP1 | -2.15862 | 2.42896 | -2.39191 | 0.032445 | -3.64873 |
| PCDHB15 | -1.4371 | -0.30076 | -3.23172 | 0.006498 | -2.20929 | SYN1 | -1.05206 | -0.56065 | -2.39176 | 0.032454 | -3.64897 |
| id | logFC | AveExpr | t | P.Value | B | id | logFC | AveExpr | t | P.Value | B |
| NUDT17 | 0.728201 | 0.005585 | 3.230008 | 0.00652 | -2.21228 | KLC3 | -1.71198 | 0.799002 | -2.39162 | 0.032463 | -3.6492 |
| TAT | 0.796727 | -1.34997 | 3.229912 | 0.006521 | -2.21244 | IFNL1 | 0.645096 | -1.37936 | 2.391177 | 0.03249 | -3.64994 |
| LSR | -1.49981 | 1.900203 | -3.22645 | 0.006565 | -2.21849 | CPEB1 | -1.67011 | 1.48508 | -2.39089 | 0.032507 | -3.65041 |
| IL10RA | -2.65372 | 0.164668 | -3.22572 | 0.006574 | -2.21977 | AEN | 0.579282 | 0.866259 | 2.390161 | 0.032552 | -3.65162 |
| DUOX2 | -2.25709 | 0.177042 | -3.22413 | 0.006594 | -2.22254 | MMP25 | -1.7786 | 1.388356 | -2.3901 | 0.032555 | -3.65171 |
| TMEM63C | -2.15623 | 0.83489 | -3.2189 | 0.006661 | -2.23168 | CABYR | 0.8561 | 1.689585 | 2.390067 | 0.032557 | -3.65177 |
| HLA-DQB1 | -2.1609 | 0.832022 | -3.21876 | 0.006663 | -2.23193 | AFMID | 0.390391 | 0.863589 | 2.389624 | 0.032585 | -3.6525 |
| GPX3 | -1.86449 | 1.76949 | -3.21872 | 0.006664 | -2.232 | KCNB1 | -1.50188 | 0.417583 | -2.3894 | 0.032599 | -3.65288 |
| CDH18 | 1.108811 | -2.06506 | 3.216524 | 0.006692 | -2.23583 | CENPO | 0.943963 | 1.780476 | 2.389386 | 0.032599 | -3.6529 |
| ATAD2B | 0.742275 | 1.074397 | 3.21595 | 0.006699 | -2.23684 | PLEKHH3 | 0.654702 | 2.161945 | 2.389098 | 0.032617 | -3.65337 |
| EPHX2 | -1.51537 | 2.213368 | -3.21521 | 0.006709 | -2.23813 | FRAS1 | 1.330997 | 3.046944 | 2.389091 | 0.032617 | -3.65338 |
| MYO10 | 0.749523 | 2.089407 | 3.213346 | 0.006733 | -2.24138 | TMEM54 | -1.55708 | 2.396195 | -2.38904 | 0.03262 | -3.65346 |
| MMAB | 0.922491 | 0.425595 | 3.211359 | 0.006759 | -2.24485 | CDK15 | 0.721288 | -2.1335 | 2.388764 | 0.032637 | -3.65392 |
| RAB6B | -1.67667 | -0.12919 | -3.21044 | 0.006771 | -2.24646 | SAMD14 | -0.85936 | 1.25251 | -2.38855 | 0.03265 | -3.65427 |
| OR51E2 | 1.625899 | -1.51894 | 3.208309 | 0.006799 | -2.25018 | MAMDC2 | -1.91325 | -0.23 | -2.38847 | 0.032655 | -3.6544 |
| SHISAL2A | -1.70043 | 0.372156 | -3.20498 | 0.006843 | -2.256 | KLK8 | -2.62075 | 0.170692 | -2.38843 | 0.032658 | -3.65448 |
| ZFP36L1 | -0.73687 | 5.426325 | -3.20492 | 0.006844 | -2.2561 | EXO1 | 1.328935 | 0.60772 | 2.388133 | 0.032676 | -3.65496 |
| FGFBP3 | 1.194086 | -0.7334 | 3.204815 | 0.006845 | -2.25629 | MPHOSPH9 | 0.581226 | 0.272999 | 2.387742 | 0.0327 | -3.65561 |
| ZNF530 | 0.77085 | -0.0228 | 3.204411 | 0.00685 | -2.25699 | ATG9A | 0.730448 | 2.914633 | 2.387329 | 0.032725 | -3.65629 |
| TNK1 | -0.70923 | 2.015628 | -3.2006 | 0.006901 | -2.26364 | LRRC52 | 1.036237 | -1.19686 | 2.387315 | 0.032726 | -3.65631 |
| ELOVL6 | 0.831097 | 2.147351 | 3.199121 | 0.006921 | -2.26623 | TXN | -0.43685 | 5.795614 | -2.38703 | 0.032744 | -3.65679 |
| RAI2 | -1.51816 | 2.449872 | -3.19897 | 0.006923 | -2.26649 | XAF1 | -1.39127 | 0.71593 | -2.38599 | 0.032807 | -3.6585 |
| TRAF3IP2 | -0.89024 | 2.062202 | -3.19871 | 0.006926 | -2.26695 | INTU | 0.551333 | 1.467987 | 2.385597 | 0.032831 | -3.65914 |
| SLC28A1 | 1.013236 | -0.99576 | 3.198504 | 0.006929 | -2.26731 | OAS2 | -1.63789 | 0.307518 | -2.38558 | 0.032832 | -3.65917 |
| KL | -1.28975 | -1.26173 | -3.19631 | 0.006958 | -2.27114 | CNTN3 | 1.26514 | 0.545842 | 2.385171 | 0.032858 | -3.65985 |
| id | logFC | AveExpr | t | P.Value | B | id | logFC | AveExpr | t | P.Value | B |
| ARSG | -1.50268 | 1.212459 | -3.19385 | 0.006992 | -2.27543 | PRPF3 | 0.365933 | 2.010922 | 2.384725 | 0.032885 | -3.66058 |
| CD8B | -2.98487 | 1.039511 | -3.19338 | 0.006998 | -2.27626 | XPNPEP1 | 0.386636 | 2.350958 | 2.384262 | 0.032914 | -3.66134 |
| MIXL1 | -1.41232 | -1.47053 | -3.19129 | 0.007026 | -2.2799 | DNMBP-AS1 | 0.342827 | -2.22765 | 2.383232 | 0.032977 | -3.66304 |
| RIT1 | 0.66043 | 0.172231 | 3.190931 | 0.007031 | -2.28053 | HACD1 | 1.278 | 2.656497 | 2.381858 | 0.033062 | -3.6653 |
| DNAJA4 | -2.15154 | 2.442857 | -3.19078 | 0.007033 | -2.28079 | SQOR | -1.61807 | 3.968289 | -2.38155 | 0.033081 | -3.66582 |
| IKZF1 | -2.59643 | 1.067574 | -3.18759 | 0.007077 | -2.28637 | NCAPG | 1.58335 | 0.376223 | 2.381508 | 0.033084 | -3.66588 |
| GATA6 | 1.608792 | 2.200691 | 3.186983 | 0.007085 | -2.28743 | API5 | -0.32455 | 1.386511 | -2.3813 | 0.033097 | -3.66622 |
| LRP11 | 0.97214 | 0.750152 | 3.186271 | 0.007095 | -2.28867 | LPCAT3 | -0.37286 | 3.655683 | -2.38076 | 0.03313 | -3.66711 |
| HRAT92 | 2.026917 | -0.73437 | 3.18536 | 0.007107 | -2.29026 | COPA | 0.76197 | 1.805519 | 2.380452 | 0.033149 | -3.66762 |
| TCF7L1 | 1.136496 | 2.534903 | 3.184139 | 0.007124 | -2.29239 | LAPTM4B | 0.848571 | 4.677263 | 2.380433 | 0.033151 | -3.66765 |
| MPP6 | 1.031841 | 0.867851 | 3.183601 | 0.007131 | -2.29333 | GID4 | -0.44578 | 2.674711 | -2.37969 | 0.033197 | -3.66888 |
| SLC15A1 | -2.28978 | 0.075042 | -3.1833 | 0.007136 | -2.29385 | GRB7 | -0.51612 | 0.930667 | -2.37955 | 0.033206 | -3.66911 |
| SP9 | 0.660866 | 0.732636 | 3.18276 | 0.007143 | -2.2948 | PDIA2 | -1.78272 | 1.429 | -2.379 | 0.03324 | -3.67001 |
| METTL4 | 0.57714 | 0.4147 | 3.182561 | 0.007146 | -2.29515 | POU2AF1 | -1.03052 | -1.73947 | -2.37884 | 0.033249 | -3.67027 |
| PLEKHA6 | -1.59495 | 1.680669 | -3.18179 | 0.007156 | -2.2965 | RAB9A | -0.48687 | 4.139812 | -2.37864 | 0.033262 | -3.6706 |
| GALK2 | -0.52274 | 3.409625 | -3.18178 | 0.007157 | -2.29652 | MITF | -1.2473 | 2.66146 | -2.37842 | 0.033276 | -3.67096 |
| MPZL2 | -2.38077 | -0.43859 | -3.18111 | 0.007166 | -2.29768 | CYP4F12 | 0.525314 | -2.10664 | 2.378223 | 0.033288 | -3.67129 |
| UACA | 0.827654 | 0.894748 | 3.179467 | 0.007189 | -2.30055 | NUP155 | 0.593612 | 1.841698 | 2.378016 | 0.033301 | -3.67163 |
| ULK4P1 | -1.95778 | -0.10783 | -3.17901 | 0.007195 | -2.30134 | TCEAL6 | -0.89811 | 4.539077 | -2.37776 | 0.033317 | -3.67205 |
| NDRG4 | -1.42816 | 3.750856 | -3.17876 | 0.007198 | -2.30178 | CNDP1 | -0.72059 | -1.85546 | -2.37775 | 0.033318 | -3.67207 |
| EVA1C | -1.54861 | 2.179977 | -3.17456 | 0.007257 | -2.30911 | FAM193A | 0.521676 | 1.440575 | 2.377513 | 0.033332 | -3.67246 |
| CECR7 | 1.189317 | -1.96588 | 3.174394 | 0.007259 | -2.30941 | NUPR2 | -0.77189 | -0.0086 | -2.37733 | 0.033344 | -3.67277 |
| WNT1 | -1.85207 | 0.433686 | -3.1742 | 0.007262 | -2.30975 | LMNB1 | 0.94023 | 1.726934 | 2.376329 | 0.033406 | -3.67441 |
| EGFEM1P | 1.954414 | -0.56984 | 3.174146 | 0.007263 | -2.30984 | OTULIN | 0.55823 | -0.51856 | 2.375902 | 0.033433 | -3.67511 |
| id | logFC | AveExpr | t | P.Value | B | id | logFC | AveExpr | t | P.Value | B |
| JAM2 | -1.11204 | 1.945504 | -3.17393 | 0.007266 | -2.31022 | TNNI3 | -1.63633 | 0.627567 | -2.37589 | 0.033434 | -3.67513 |
| GLT1D1 | -2.7881 | 0.581775 | -3.17373 | 0.007269 | -2.31056 | EPHX1 | 0.73134 | 2.869314 | 2.375318 | 0.03347 | -3.67607 |
| OPHN1 | 1.080467 | 0.716381 | 3.170996 | 0.007307 | -2.31534 | MED29 | 0.821168 | -2.26595 | 2.374918 | 0.033495 | -3.67673 |
| HOXC11 | 2.021844 | -2.00131 | 3.167881 | 0.007351 | -2.32078 | QSOX1 | 1.011605 | 3.102664 | 2.374767 | 0.033504 | -3.67698 |
| TMEM229B | -1.13508 | 1.389658 | -3.16506 | 0.007391 | -2.3257 | ZGRF1 | 0.567555 | 1.056952 | 2.374623 | 0.033513 | -3.67722 |
| FANCD2 | 1.253439 | -0.41375 | 3.161901 | 0.007437 | -2.33122 | NFIA | 1.110699 | -0.86274 | 2.373121 | 0.033608 | -3.67969 |
| RAB31 | -0.96141 | 4.468598 | -3.15841 | 0.007487 | -2.33732 | SYCE2 | 0.735561 | -1.08379 | 2.372328 | 0.033658 | -3.68099 |
| IARS2 | 0.464311 | 4.259923 | 3.157495 | 0.0075 | -2.33891 | PRTG | -1.18803 | -1.2571 | -2.37196 | 0.033681 | -3.68159 |
| GPC4 | 1.238399 | 3.439419 | 3.15655 | 0.007514 | -2.34056 | ANGPTL3 | 0.423807 | -2.3452 | 2.370725 | 0.033759 | -3.68363 |
| CHD3 | 0.881539 | 4.373877 | 3.155164 | 0.007534 | -2.34298 | ATG4D | 0.457788 | 0.058277 | 2.370595 | 0.033767 | -3.68384 |
| GFOD1 | 1.013709 | 2.819651 | 3.153736 | 0.007555 | -2.34547 | OR2M2 | 0.752562 | -1.89861 | 2.369269 | 0.033851 | -3.68602 |
| TTC13 | 0.878996 | -0.04737 | 3.152445 | 0.007574 | -2.34772 | CCDC18 | 0.68674 | 0.621211 | 2.369113 | 0.033861 | -3.68628 |
| RGMB | 1.1115 | 0.890364 | 3.148575 | 0.007631 | -2.35448 | GUCA2A | 0.460774 | -1.62232 | 2.368671 | 0.033889 | -3.68701 |
| DSCAM | 0.77037 | -1.20364 | 3.148086 | 0.007638 | -2.35533 | NCKAP5L | 0.401947 | 4.066429 | 2.368602 | 0.033893 | -3.68712 |
| TP53 | 0.735846 | -0.03445 | 3.145427 | 0.007677 | -2.35997 | LOXL2 | 1.63189 | 5.500002 | 2.368559 | 0.033896 | -3.68719 |
| KRT33B | 1.075145 | -0.71923 | 3.143735 | 0.007702 | -2.36293 | ATAD2 | 0.815403 | 2.683244 | 2.368504 | 0.033899 | -3.68728 |
| BMP4 | 2.389242 | 0.983311 | 3.14181 | 0.007731 | -2.36628 | SEC62 | -0.8556 | -1.42777 | -2.36814 | 0.033922 | -3.68788 |
| MYL4 | 0.480778 | -1.01179 | 3.140497 | 0.007751 | -2.36858 | RAMP1 | -1.40406 | 6.063942 | -2.36794 | 0.033935 | -3.68821 |
| HULC | 1.394953 | -1.03006 | 3.139965 | 0.007759 | -2.3695 | EPN3 | -1.15339 | -1.28633 | -2.36791 | 0.033937 | -3.68826 |
| BAX | 0.846511 | 0.858023 | 3.13901 | 0.007773 | -2.37117 | OR5H6 | 0.734729 | -1.72672 | 2.367781 | 0.033945 | -3.68847 |
| RAB3D | -0.6129 | -0.03872 | -3.13859 | 0.007779 | -2.3719 | TMEM151B | -1.15567 | 1.040229 | -2.36773 | 0.033949 | -3.68856 |
| TLE2 | -1.51407 | 3.486055 | -3.1379 | 0.00779 | -2.37311 | ZDHHC8P1 | -1.33302 | -0.29953 | -2.36721 | 0.033981 | -3.6894 |
| PSCA | 1.260531 | -0.25743 | 3.137808 | 0.007791 | -2.37327 | HIC2 | 0.689958 | 2.002025 | 2.367081 | 0.03399 | -3.68962 |
| LINC00032 | 0.692744 | -2.12261 | 3.133288 | 0.007859 | -2.38115 | CMYA5 | -1.28175 | -0.30907 | -2.36691 | 0.034001 | -3.68991 |
| SERHL2 | -1.21422 | 1.277042 | -3.13131 | 0.007889 | -2.38461 | DSC2 | -1.6281 | 0.015261 | -2.36581 | 0.034071 | -3.69172 |
| id | logFC | AveExpr | t | P.Value | B | id | logFC | AveExpr | t | P.Value | B |
| SMIM10 | -1.28209 | 0.940422 | -3.1304 | 0.007903 | -2.3862 | MMP11 | -1.49472 | 3.070315 | -2.36568 | 0.034079 | -3.69192 |
| DENND2D | -1.95599 | 1.412281 | -3.12972 | 0.007914 | -2.38738 | OR4Q3 | 0.570854 | -1.59007 | 2.365055 | 0.034119 | -3.69295 |
| PTPRN2 | -1.83492 | -0.11014 | -3.12933 | 0.00792 | -2.38805 | PDE6A | 0.337662 | -2.1815 | 2.364973 | 0.034124 | -3.69309 |
| ARL9 | -2.01518 | 1.506857 | -3.12911 | 0.007923 | -2.38844 | DOCK5 | 0.88201 | 1.34373 | 2.364707 | 0.034141 | -3.69352 |
| CWH43 | -1.80289 | -0.90405 | -3.1284 | 0.007934 | -2.38968 | HPDL | -1.56727 | 1.71365 | -2.36408 | 0.034181 | -3.69456 |
| SPRN | -1.44222 | 2.039571 | -3.12831 | 0.007935 | -2.38985 | LY6G5B | 0.683995 | 0.355619 | 2.364037 | 0.034184 | -3.69462 |
| ROR1 | 1.238942 | 0.562565 | 3.127741 | 0.007944 | -2.39083 | MCM3AP | 0.329452 | 3.511986 | 2.363798 | 0.034199 | -3.69502 |
| SIGIRR | -1.49795 | 3.852252 | -3.12744 | 0.007949 | -2.39136 | H2AW | 0.824062 | -0.17818 | 2.363742 | 0.034203 | -3.69511 |
| SLC22A13 | 1.038924 | -1.51732 | 3.124724 | 0.00799 | -2.39609 | TNFSF8 | 0.510681 | -1.30838 | 2.363341 | 0.034228 | -3.69577 |
| CRAT | -0.47526 | 4.460196 | -3.12453 | 0.007993 | -2.39643 | IQCK | -0.65263 | 2.677923 | -2.36321 | 0.034237 | -3.69599 |
| FNDC5 | -1.67979 | 1.363229 | -3.12444 | 0.007995 | -2.3966 | DIRAS3 | -2.02794 | 0.676153 | -2.36264 | 0.034273 | -3.69691 |
| SH3TC1 | -1.80567 | -0.45581 | -3.12068 | 0.008053 | -2.40315 | PLEKHA2 | -0.5799 | 4.175728 | -2.3623 | 0.034295 | -3.69748 |
| KRTDAP | -1.54679 | -1.06269 | -3.11934 | 0.008074 | -2.40549 | TMC8 | -1.74639 | -0.72745 | -2.36164 | 0.034337 | -3.69856 |
| MYO7A | -1.56641 | -0.34468 | -3.1193 | 0.008075 | -2.40556 | TRIM40 | 0.807519 | -2.02911 | 2.361396 | 0.034353 | -3.69896 |
| XIRP1 | 0.899213 | -1.39093 | 3.11739 | 0.008104 | -2.40889 | TMEM125 | -2.12286 | 0.618861 | -2.36136 | 0.034355 | -3.69902 |
| IGF2BP1 | 1.470792 | -0.8019 | 3.116649 | 0.008116 | -2.41018 | OR2A5 | 0.735049 | -1.47578 | 2.360969 | 0.034381 | -3.69966 |
| IL1R1 | -1.34868 | 1.765837 | -3.11621 | 0.008123 | -2.41094 | FAM210B | -0.46073 | 4.285266 | -2.36091 | 0.034385 | -3.69977 |
| PRXL2A | -2.35329 | 3.398553 | -3.11498 | 0.008142 | -2.4131 | CDK2AP2 | -0.61612 | 4.708651 | -2.36016 | 0.034433 | -3.70099 |
| TMEM164 | 0.56305 | 1.096404 | 3.1142 | 0.008154 | -2.41445 | PIK3R1 | -0.89641 | 2.962089 | -2.35952 | 0.034474 | -3.70205 |
| MAP3K13 | 0.591721 | 0.047963 | 3.113658 | 0.008163 | -2.4154 | SNCG | 0.419581 | -0.11559 | 2.35941 | 0.034481 | -3.70222 |
| AMBN | 3.084415 | -0.02796 | 3.113047 | 0.008173 | -2.41646 | CPSF4 | 0.430755 | 3.080548 | 2.358596 | 0.034533 | -3.70356 |
| MCF2L-AS1 | -2.25436 | 0.87162 | -3.11268 | 0.008178 | -2.4171 | BPNT1 | 0.527748 | 2.239397 | 2.358575 | 0.034535 | -3.70359 |
| OSR1 | 1.138937 | -1.89444 | 3.110288 | 0.008216 | -2.42127 | LINC00471 | 0.672185 | -0.99795 | 2.357386 | 0.034612 | -3.70555 |
| BICC1 | 0.879719 | -2.27534 | 3.106279 | 0.00828 | -2.42826 | SRP9 | 0.75709 | -1.9305 | 2.357248 | 0.034621 | -3.70577 |
| PEG10 | 2.554005 | 0.665054 | 3.106166 | 0.008282 | -2.42846 | ZNF45 | 0.347309 | 0.735564 | 2.356631 | 0.034661 | -3.70679 |
| id | logFC | AveExpr | t | P.Value | B | id | logFC | AveExpr | t | P.Value | B |
| XGY2 | 0.827038 | -2.07581 | 3.10611 | 0.008283 | -2.42856 | LINC01550 | 0.917384 | -1.69121 | 2.356537 | 0.034667 | -3.70694 |
| C16orf82 | 0.946258 | -1.22664 | 3.10608 | 0.008283 | -2.42861 | S1PR4 | -2.03823 | 0.107083 | -2.35639 | 0.034676 | -3.70718 |
| HAAO | -1.37552 | 1.248044 | -3.10548 | 0.008293 | -2.42965 | ARL8B | -0.35563 | 3.235467 | -2.35635 | 0.034679 | -3.70724 |
| HSD52 | 0.969972 | -2.05175 | 3.104463 | 0.008309 | -2.43143 | SLC22A10 | 0.490176 | -0.75768 | 2.356038 | 0.034699 | -3.70776 |
| CAND1 | 0.559021 | 0.905696 | 3.102242 | 0.008345 | -2.4353 | CHCHD10 | -1.44687 | 3.648489 | -2.35584 | 0.034712 | -3.70809 |
| STAT5B | -0.91827 | 2.1292 | -3.09937 | 0.008391 | -2.44031 | HCST | -1.5456 | 2.286059 | -2.35569 | 0.034721 | -3.70832 |
| CALML5 | 0.873163 | -0.8284 | 3.099266 | 0.008393 | -2.44049 | CD52 | -2.36984 | 0.909715 | -2.35559 | 0.034728 | -3.70849 |
| AIFM3 | -1.88359 | -0.13564 | -3.09842 | 0.008407 | -2.44196 | EXTL3 | 0.550038 | 4.715933 | 2.355314 | 0.034746 | -3.70895 |
| TARBP1 | 1.032928 | 1.147906 | 3.098376 | 0.008407 | -2.44204 | DCAF4L2 | 0.630864 | -1.478 | 2.354878 | 0.034774 | -3.70966 |
| DENND1C | -1.76006 | -0.09404 | -3.09566 | 0.008452 | -2.44678 | ZNF283 | 0.446923 | 1.907389 | 2.354174 | 0.03482 | -3.71082 |
| PRM1 | 0.488425 | -0.62454 | 3.095478 | 0.008455 | -2.4471 | GIMAP5 | -2.07561 | -0.92563 | -2.35276 | 0.034912 | -3.71313 |
| PNN | 0.762399 | -0.30586 | 3.0942 | 0.008476 | -2.44932 | SPATA31D3 | 0.789554 | -1.60287 | 2.352365 | 0.034938 | -3.71379 |
| LITAF | -1.14063 | 2.354812 | -3.09396 | 0.00848 | -2.44974 | PTAFR | -1.67428 | -0.73205 | -2.35233 | 0.03494 | -3.71384 |
| LINC00163 | 0.776408 | -2.28434 | 3.093444 | 0.008488 | -2.45064 | SPATA22 | 0.648068 | -1.78251 | 2.35229 | 0.034943 | -3.71391 |
| TRABD2B | 0.888126 | -0.6171 | 3.091533 | 0.008519 | -2.45397 | DLK2 | -1.38661 | 2.562713 | -2.3519 | 0.034968 | -3.71455 |
| TNFSF13 | -1.63618 | 0.638835 | -3.08985 | 0.008547 | -2.45691 | CCDC85A | 1.327902 | 0.041018 | 2.351702 | 0.034981 | -3.71487 |
| KCTD15 | 1.169724 | 0.782754 | 3.088852 | 0.008564 | -2.45865 | GFY | -1.16593 | 0.171957 | -2.3511 | 0.03502 | -3.71585 |
| AMIGO3 | 0.72 | 1.231839 | 3.08862 | 0.008567 | -2.45905 | FAM3C | -0.73772 | 2.994214 | -2.35019 | 0.03508 | -3.71734 |
| LOC388242 | -1.24755 | 1.952395 | -3.08843 | 0.00857 | -2.45937 | FOXN1 | 0.396906 | -0.60657 | 2.350136 | 0.035084 | -3.71744 |
| FAT1 | 1.58717 | 3.744343 | 3.088203 | 0.008574 | -2.45978 | SOX12 | 0.946809 | -0.25768 | 2.350032 | 0.035091 | -3.71761 |
| PWWP3B | -3.39748 | 1.584987 | -3.08664 | 0.0086 | -2.4625 | NEK8 | 0.899823 | -0.56088 | 2.349649 | 0.035116 | -3.71824 |
| IFITM3 | -1.08122 | 6.913363 | -3.08465 | 0.008633 | -2.46596 | UBR4 | 0.478178 | 5.558386 | 2.349615 | 0.035118 | -3.71829 |
| GATA5 | -2.26104 | -0.32211 | -3.08344 | 0.008654 | -2.46808 | SPANXA2-OT1 | 0.371083 | -2.51991 | 2.349187 | 0.035146 | -3.719 |
| TMPRSS15 | 0.684735 | -1.89598 | 3.081549 | 0.008685 | -2.47137 | DOLK | 0.633324 | 3.004737 | 2.348497 | 0.035191 | -3.72013 |
| id | logFC | AveExpr | t | P.Value | B | id | logFC | AveExpr | t | P.Value | B |
| ZNF789 | 0.767952 | 1.492942 | 3.078202 | 0.008741 | -2.47721 | MPP5 | 0.57624 | 2.380962 | 2.347921 | 0.035229 | -3.72107 |
| ASB13 | -0.53934 | 2.324575 | -3.0779 | 0.008747 | -2.47773 | INTS3 | 0.512345 | 2.209185 | 2.347182 | 0.035278 | -3.72228 |
| PRRC2C | 0.647265 | 2.67554 | 3.077654 | 0.008751 | -2.47816 | ORC1 | 1.245957 | 0.099658 | 2.346652 | 0.035313 | -3.72315 |
| KRTAP4-7 | 0.553004 | -0.50901 | 3.07761 | 0.008751 | -2.47824 | MCM2 | 0.903581 | 3.394801 | 2.346619 | 0.035315 | -3.72321 |
| PLK4 | 1.118791 | -1.35802 | 3.070942 | 0.008865 | -2.48986 | FAM81A | -1.64294 | -0.54378 | -2.34623 | 0.035341 | -3.72385 |
| VSIG2 | -2.0335 | -0.29956 | -3.07062 | 0.00887 | -2.49042 | ASS1 | -1.10065 | 2.816777 | -2.3461 | 0.035349 | -3.72406 |
| ZC3H12C | 0.879444 | 1.30372 | 3.07013 | 0.008879 | -2.49127 | FBXO41 | -0.78138 | 3.065581 | -2.34608 | 0.035351 | -3.72409 |
| GGT5 | -2.12177 | 3.613132 | -3.06976 | 0.008885 | -2.49191 | VAMP1 | 0.887587 | -0.33843 | 2.345903 | 0.035362 | -3.72438 |
| FGD3 | -2.21146 | 0.31364 | -3.06891 | 0.0089 | -2.49339 | R3HCC1 | -0.38355 | 3.303616 | -2.34537 | 0.035398 | -3.72526 |
| CPLX1 | -2.5949 | 1.658705 | -3.06843 | 0.008908 | -2.49423 | PLPPR5 | -1.60348 | -1.18613 | -2.3449 | 0.035429 | -3.72602 |
| UCHL1 | 1.095761 | 5.922481 | 3.067348 | 0.008927 | -2.49612 | ACAN | 0.796796 | -1.08209 | 2.344282 | 0.03547 | -3.72703 |
| TMEM147 | -0.41276 | 4.897229 | -3.06732 | 0.008927 | -2.49616 | LGALSL | -0.94734 | 2.762592 | -2.34422 | 0.035473 | -3.72713 |
| ADAM11 | -1.75382 | 1.078369 | -3.06708 | 0.008931 | -2.49658 | METTL24 | -1.21491 | -1.28251 | -2.34401 | 0.035487 | -3.72747 |
| ZNF700 | 0.669841 | -0.11888 | 3.066814 | 0.008936 | -2.49705 | SFTPD | -0.52651 | 0.151813 | -2.34302 | 0.035553 | -3.7291 |
| POLE | 1.099925 | 0.929353 | 3.066767 | 0.008937 | -2.49713 | LAYN | -0.86307 | 3.370567 | -2.34233 | 0.035599 | -3.73023 |
| SPINT2 | -2.45745 | 3.198727 | -3.06586 | 0.008952 | -2.49871 | GALNT1 | 0.531018 | 2.278012 | 2.342278 | 0.035603 | -3.73032 |
| CYGB | -0.83838 | 4.536259 | -3.06507 | 0.008966 | -2.50008 | ZBTB8A | -0.49897 | 2.494614 | -2.34216 | 0.03561 | -3.7305 |
| GRID2 | 0.609338 | -0.61497 | 3.061881 | 0.009021 | -2.50564 | EFNB1 | 0.47573 | 3.550375 | 2.341844 | 0.035631 | -3.73103 |
| MED28 | 0.840776 | -1.42165 | 3.061168 | 0.009034 | -2.50688 | SLC12A2 | 0.775199 | 1.050872 | 2.341304 | 0.035667 | -3.73191 |
| MSH6 | 0.659231 | 4.210438 | 3.060003 | 0.009054 | -2.50891 | PRKAB2 | 0.790972 | 1.963174 | 2.341004 | 0.035687 | -3.7324 |
| GBP2 | -0.89277 | 2.40766 | -3.05933 | 0.009066 | -2.51008 | NRP2 | 1.218446 | 1.804645 | 2.340388 | 0.035728 | -3.73341 |
| AWAT2 | 0.604432 | -2.21918 | 3.054592 | 0.009149 | -2.51833 | IKBKE | 0.776902 | -0.29593 | 2.339857 | 0.035764 | -3.73428 |
| TDRD10 | -1.54102 | 0.407743 | -3.05402 | 0.009159 | -2.51934 | VWA1 | -1.25177 | 3.295906 | -2.33921 | 0.035807 | -3.73534 |
| CEP97 | 0.606476 | -0.86687 | 3.054005 | 0.00916 | -2.51935 | FZD8 | 1.314998 | 0.450272 | 2.338494 | 0.035855 | -3.73651 |
| SLC14A1 | 1.308369 | -1.61865 | 3.052784 | 0.009181 | -2.52148 | BNIP1 | 0.439756 | 1.469371 | 2.338258 | 0.035871 | -3.7369 |
| id | logFC | AveExpr | t | P.Value | B | id | logFC | AveExpr | t | P.Value | B |
| LY75 | -1.40757 | -1.50666 | -3.04994 | 0.009232 | -2.52643 | CDC25A | 1.071988 | 1.221034 | 2.337967 | 0.03589 | -3.73737 |
| UCK2 | 0.95511 | 2.150599 | 3.049074 | 0.009247 | -2.52794 | PLK1 | 1.396741 | 1.016978 | 2.337325 | 0.035933 | -3.73842 |
| IRS1 | -1.07273 | 1.374578 | -3.04828 | 0.009261 | -2.52932 | SEPTIN3 | -1.24752 | -0.00806 | -2.33679 | 0.035969 | -3.73929 |
| LORICRIN | -1.79004 | -1.13145 | -3.0479 | 0.009268 | -2.52998 | HJURP | 1.751904 | 1.444906 | 2.336302 | 0.036002 | -3.7401 |
| ASXL3 | -1.35645 | -1.11243 | -3.04657 | 0.009292 | -2.5323 | HOOK1 | -1.51304 | -0.98693 | -2.33628 | 0.036003 | -3.74013 |
| SOX9 | 1.299341 | -0.76725 | 3.046378 | 0.009295 | -2.53263 | HTR5A | 0.354121 | -2.18367 | 2.335989 | 0.036023 | -3.74061 |
| FMN2 | 1.337776 | -0.8985 | 3.046376 | 0.009295 | -2.53264 | COLGALT2 | 1.175388 | -0.72236 | 2.335974 | 0.036024 | -3.74063 |
| ARHGAP35 | 0.495708 | 2.52198 | 3.045893 | 0.009304 | -2.53348 | GSC | 1.60291 | 0.581629 | 2.335886 | 0.03603 | -3.74078 |
| CCND1 | 1.421524 | 3.030863 | 3.045682 | 0.009308 | -2.53385 | OR52I1 | -0.98669 | -2.02015 | -2.33367 | 0.036179 | -3.74441 |
| MAST1 | -2.26716 | 1.88627 | -3.04532 | 0.009314 | -2.53447 | CIRBP-AS1 | 0.618083 | 0.775219 | 2.333637 | 0.036181 | -3.74446 |
| TECPR1 | 0.646881 | 3.274886 | 3.042225 | 0.00937 | -2.53986 | LINC00842 | 1.429914 | 1.710597 | 2.332542 | 0.036255 | -3.74625 |
| FAM72D | 1.377175 | 0.503679 | 3.041823 | 0.009377 | -2.54056 | CTRL | 0.610302 | -0.37142 | 2.332404 | 0.036265 | -3.74647 |
| NGB | -2.525 | -0.407 | -3.03971 | 0.009416 | -2.54424 | CSPG5 | -1.96403 | -0.2411 | -2.33237 | 0.036267 | -3.74653 |
| CCDC150 | 1.42506 | -0.65837 | 3.037119 | 0.009463 | -2.54875 | ATP1B1 | 0.774985 | 4.642298 | 2.332177 | 0.03628 | -3.74684 |
| SH3BGRL | -0.59514 | 3.820556 | -3.03631 | 0.009478 | -2.55015 | OR2T4 | 0.690514 | -1.67033 | 2.332042 | 0.036289 | -3.74706 |
| PITX2 | 2.724821 | -0.95836 | 3.034873 | 0.009504 | -2.55266 | CACNB1 | 0.699891 | 0.178816 | 2.331661 | 0.036315 | -3.74769 |
| HOXA5 | 1.909534 | 3.230376 | 3.033508 | 0.009529 | -2.55504 | SLC7A10 | -1.98823 | -0.75188 | -2.33148 | 0.036327 | -3.74799 |
| PSPN | 0.719606 | 0.879026 | 3.033078 | 0.009537 | -2.55578 | R3HDM1 | 0.467314 | 0.082039 | 2.331403 | 0.036332 | -3.74811 |
| HOXC4 | 1.945276 | 1.827664 | 3.029944 | 0.009595 | -2.56124 | SPTBN5 | 1.128959 | -0.19415 | 2.331122 | 0.036351 | -3.74857 |
| PBX1 | -1.12461 | 3.134801 | -3.02925 | 0.009608 | -2.56244 | TTC39A | -1.23276 | -1.00877 | -2.33098 | 0.036361 | -3.74881 |
| THEMIS2 | -1.25898 | 1.380746 | -3.02919 | 0.009609 | -2.56256 | COL5A3 | 1.198746 | 1.80837 | 2.330157 | 0.036417 | -3.75015 |
| TRIM2 | 0.787585 | 0.911825 | 3.028196 | 0.009627 | -2.56428 | NRG4 | 0.747758 | -1.2003 | 2.330017 | 0.036426 | -3.75038 |
| IL18RAP | -1.37997 | -0.67345 | -3.02674 | 0.009654 | -2.56681 | PTPRA | -0.30474 | 4.174986 | -2.32986 | 0.036437 | -3.75062 |
| KIF18A | 1.568587 | -0.96264 | 3.025882 | 0.00967 | -2.5683 | KCNA7 | -1.6937 | -1.04662 | -2.32949 | 0.036462 | -3.75123 |
| HSD17B8 | -1.06627 | 1.664623 | -3.0258 | 0.009672 | -2.56845 | RNF123 | 0.487661 | 2.53631 | 2.32884 | 0.036507 | -3.7523 |
| id | logFC | AveExpr | t | P.Value | B | id | logFC | AveExpr | t | P.Value | B |
| H2AC13 | 1.035182 | -1.40939 | 3.024837 | 0.00969 | -2.57012 | A1BG-AS1 | -0.67565 | 2.604856 | -2.32788 | 0.036572 | -3.75388 |
| NOS3 | -1.46413 | 2.515942 | -3.02346 | 0.009716 | -2.57252 | TRIM14 | -1.48381 | 0.627722 | -2.32759 | 0.036592 | -3.75435 |
| E2F7 | 1.69166 | 1.318682 | 3.022493 | 0.009734 | -2.5742 | SPHK1 | 0.647004 | 4.944825 | 2.327026 | 0.03663 | -3.75526 |
| DMRTA1 | 0.773017 | -1.07991 | 3.020702 | 0.009768 | -2.57731 | CADPS | 1.287367 | 0.634275 | 2.325966 | 0.036703 | -3.75699 |
| ELAPOR1 | -1.38791 | 1.371956 | -3.0201 | 0.009779 | -2.57836 | CHEK1 | 0.953514 | 1.308682 | 2.325381 | 0.036743 | -3.75795 |
| CAPN10 | 0.590486 | -1.96832 | 3.018887 | 0.009802 | -2.58047 | PSTPIP1 | -1.61637 | 1.120457 | -2.32471 | 0.036788 | -3.75904 |
| DLGAP3 | -1.60153 | 1.59413 | -3.01841 | 0.009811 | -2.5813 | RECQL5 | 0.607993 | 0.616986 | 2.324214 | 0.036823 | -3.75986 |
| CYP4F22 | -1.88472 | -1.15179 | -3.01481 | 0.009879 | -2.58757 | UBALD2 | -0.51739 | 3.960165 | -2.324 | 0.036838 | -3.76021 |
| SLC35G1 | 0.77522 | -0.49966 | 3.014449 | 0.009886 | -2.58819 | ZNF253 | -0.57489 | -1.98243 | -2.32318 | 0.036894 | -3.76155 |
| ZNF880 | 0.909191 | -0.24581 | 3.014119 | 0.009892 | -2.58877 | CBX4 | 1.208099 | 1.233001 | 2.323048 | 0.036903 | -3.76176 |
| LHFPL3-AS1 | 0.800416 | -2.18245 | 3.013499 | 0.009904 | -2.58984 | DIPK2A | 0.881428 | 0.187918 | 2.322589 | 0.036934 | -3.76251 |
| MANSC1 | -1.33213 | -0.56314 | -3.01293 | 0.009915 | -2.59082 | ZNF285 | 0.719518 | -0.81701 | 2.322465 | 0.036943 | -3.76271 |
| HLA-DPB1 | -2.43491 | 2.645823 | -3.01238 | 0.009926 | -2.59179 | LRIG1 | 0.830378 | 3.853601 | 2.322088 | 0.036969 | -3.76333 |
| ISLR | -2.687 | 5.574681 | -3.01164 | 0.00994 | -2.59308 | PABPC1P2 | 0.700859 | -1.98757 | 2.321546 | 0.037006 | -3.76421 |
| TP53AIP1 | 0.86128 | -1.14062 | 3.011327 | 0.009946 | -2.59362 | MAN1B1 | 0.555315 | 4.23369 | 2.321505 | 0.037009 | -3.76428 |
| BEGAIN | -1.19452 | 2.891147 | -3.00957 | 0.00998 | -2.59667 | ZNF589 | 0.532959 | -0.35551 | 2.321483 | 0.037011 | -3.76431 |
| NCSTN | 0.596931 | 4.647034 | 3.00744 | 0.010021 | -2.60038 | CCN6 | -1.4079 | -1.5326 | -2.32101 | 0.037043 | -3.76508 |
| ESAM | -1.20451 | 1.129111 | -3.0072 | 0.010025 | -2.60079 | GLB1L | 0.868867 | 1.596328 | 2.320965 | 0.037046 | -3.76516 |
| TEX19 | 1.163824 | -2.21354 | 3.006509 | 0.010039 | -2.602 | ARF3 | -0.38828 | 4.865729 | -2.32079 | 0.037058 | -3.76544 |
| ATP7B | 0.961609 | 2.499691 | 3.005694 | 0.010055 | -2.60341 | HEATR1 | 0.361969 | 2.86909 | 2.320581 | 0.037073 | -3.76579 |
| GSTTP2 | 0.657678 | -1.85285 | 3.004379 | 0.01008 | -2.6057 | SPATA24 | 0.574943 | -2.33891 | 2.319943 | 0.037117 | -3.76683 |
| CELA2A | 0.843275 | -0.75135 | 3.003969 | 0.010088 | -2.60641 | SNX19 | 0.522833 | 3.156026 | 2.319559 | 0.037144 | -3.76745 |
| FRK | 0.600034 | -2.27531 | 3.003177 | 0.010104 | -2.60779 | RGCC | -2.21255 | 2.490138 | -2.31901 | 0.037182 | -3.76835 |
| NDRG2 | -1.43555 | 4.7947 | -3.002 | 0.010126 | -2.60983 | PARD6B | -0.93013 | -1.58928 | -2.31865 | 0.037206 | -3.76894 |
| CAPRIN2 | 0.795856 | 1.902649 | 2.999783 | 0.01017 | -2.61369 | SPRR2G | 0.714975 | -0.92528 | 2.318581 | 0.037211 | -3.76905 |
| id | logFC | AveExpr | t | P.Value | B | id | logFC | AveExpr | t | P.Value | B |
| TTC21B | 0.471101 | 1.06042 | 2.997889 | 0.010207 | -2.61698 | BTNL9 | -1.35447 | -1.32496 | -2.31831 | 0.03723 | -3.7695 |
| EPHA5 | 2.080445 | -0.8225 | 2.997241 | 0.01022 | -2.61811 | ICA1 | -0.99532 | 1.682634 | -2.31811 | 0.037244 | -3.76981 |
| PRB3 | 0.436399 | 1.268677 | 2.996692 | 0.010231 | -2.61906 | ARHGAP9 | -1.95124 | 1.132607 | -2.31761 | 0.037279 | -3.77064 |
| AP4B1 | 0.558122 | 0.928086 | 2.994922 | 0.010266 | -2.62214 | LTK | -1.57459 | 3.582952 | -2.31646 | 0.037359 | -3.77251 |
| RASSF2 | -1.32093 | 3.20191 | -2.99233 | 0.010317 | -2.62663 | RGS7 | 1.198876 | -1.47453 | 2.316229 | 0.037375 | -3.77289 |
| ETV6 | 0.450579 | 1.571473 | 2.988126 | 0.010401 | -2.63395 | OR2AG1 | 0.704008 | -1.01214 | 2.314701 | 0.037481 | -3.77538 |
| SCT | -2.24736 | 0.586125 | -2.98759 | 0.010412 | -2.63487 | SELENOK | -0.60838 | 5.179132 | -2.31429 | 0.03751 | -3.77605 |
| PFAS | 0.828039 | 0.557633 | 2.986719 | 0.010429 | -2.63639 | PPY2P | 0.806413 | -1.87635 | 2.313953 | 0.037533 | -3.7766 |
| EPO | -1.94565 | -0.4957 | -2.9867 | 0.01043 | -2.63643 | PPARA | -0.4513 | 0.635521 | -2.31206 | 0.037666 | -3.77968 |
| FGF6 | 0.524614 | -1.83105 | 2.98665 | 0.010431 | -2.63651 | APLP2 | 0.57209 | 5.399601 | 2.311985 | 0.037671 | -3.77981 |
| PALMD | -2.52855 | 1.285792 | -2.98594 | 0.010445 | -2.63775 | AURKB | 1.468615 | 0.636856 | 2.311977 | 0.037672 | -3.77982 |
| TNFRSF18 | -1.87932 | 0.457985 | -2.98366 | 0.010491 | -2.6417 | NR1H4 | 0.522552 | -1.91541 | 2.310942 | 0.037744 | -3.7815 |
| KLHDC4 | 0.461345 | 1.785706 | 2.982717 | 0.01051 | -2.64334 | REEP6 | -1.41002 | 3.406278 | -2.31053 | 0.037773 | -3.78217 |
| PAK6 | -1.81894 | 0.477057 | -2.98249 | 0.010515 | -2.64373 | ABLIM2 | -1.70917 | 0.522942 | -2.30996 | 0.037814 | -3.78311 |
| NEO1 | 1.247039 | 0.29535 | 2.981799 | 0.010529 | -2.64494 | GIMAP7 | -2.81889 | -0.59479 | -2.30987 | 0.03782 | -3.78326 |
| NUTM2F | 1.086232 | -0.84705 | 2.980193 | 0.010562 | -2.64773 | TSR1 | 0.430003 | 2.668926 | 2.309639 | 0.037836 | -3.78363 |
| RNF135 | -0.74911 | 3.55773 | -2.97981 | 0.010569 | -2.64838 | LARGE2 | -1.40057 | 1.393856 | -2.30887 | 0.03789 | -3.78487 |
| TMEM31 | -1.26706 | -1.38987 | -2.97882 | 0.010589 | -2.65011 | ERI2 | 0.523739 | 0.359024 | 2.308791 | 0.037896 | -3.78501 |
| FXYD6 | -1.54755 | 2.58536 | -2.97865 | 0.010593 | -2.65041 | MYBPHL | 1.086307 | -1.24039 | 2.30867 | 0.037904 | -3.78521 |
| GPR19 | 1.085012 | -1.84714 | 2.977497 | 0.010617 | -2.65241 | PIK3CG | -1.6975 | -1.05068 | -2.30816 | 0.037941 | -3.78604 |
| TGM7 | 0.959295 | -1.32069 | 2.977283 | 0.010621 | -2.65278 | CPNE4 | -1.91111 | -1.40244 | -2.30781 | 0.037965 | -3.7866 |
| PI16 | 1.663048 | -0.03518 | 2.975654 | 0.010654 | -2.65561 | DNM1P35 | 0.687843 | -1.15896 | 2.3072 | 0.038008 | -3.7876 |
| CYB5RL | 0.691334 | -2.09497 | 2.974837 | 0.010671 | -2.65703 | TPTEP2 | 0.904409 | 0.504867 | 2.307019 | 0.038021 | -3.7879 |
| LRRN2 | -0.81974 | 2.755845 | -2.97442 | 0.01068 | -2.65776 | WDR89 | 0.476838 | -0.50181 | 2.306729 | 0.038042 | -3.78837 |
| IL18R1 | -1.35568 | -0.69448 | -2.97406 | 0.010687 | -2.65837 | ATL2 | 0.588388 | 2.395307 | 2.306589 | 0.038052 | -3.7886 |
| id | logFC | AveExpr | t | P.Value | B | id | logFC | AveExpr | t | P.Value | B |
| TNNC1 | 1.304325 | -0.91105 | 2.973239 | 0.010704 | -2.6598 | TEX22 | 0.961993 | -0.41511 | 2.305158 | 0.038153 | -3.79093 |
| TICRR | 1.823624 | -0.82516 | 2.972646 | 0.010716 | -2.66083 | POLR1A | 0.571249 | 2.632022 | 2.304994 | 0.038165 | -3.79119 |
| PSTK | 0.394291 | -0.02563 | 2.966556 | 0.010843 | -2.67141 | DNAJC16 | 0.453352 | 1.479006 | 2.304701 | 0.038185 | -3.79167 |
| RNF175 | -2.37303 | 0.582893 | -2.9653 | 0.010869 | -2.67358 | PLCD1 | -0.6647 | 3.410745 | -2.30456 | 0.038195 | -3.7919 |
| CLIC2 | -1.81647 | 0.626296 | -2.96421 | 0.010892 | -2.67548 | SLFN12 | 0.960316 | -0.0393 | 2.304176 | 0.038223 | -3.79252 |
| DEPDC5 | 0.650602 | -0.28264 | 2.963105 | 0.010915 | -2.6774 | IRF9 | -0.62278 | 2.397253 | -2.30373 | 0.038255 | -3.79326 |
| SPON2 | -1.69567 | 6.38823 | -2.96268 | 0.010924 | -2.67813 | ZMYM2 | 0.489972 | 1.677939 | 2.303619 | 0.038262 | -3.79343 |
| RAB38 | -2.232 | -0.23301 | -2.96194 | 0.01094 | -2.67942 | TSSK3 | 0.482431 | -0.92021 | 2.302915 | 0.038313 | -3.79458 |
| SKAP2 | 1.527535 | 1.36554 | 2.960419 | 0.010972 | -2.68206 | FUT1 | -1.2605 | 1.004467 | -2.30228 | 0.038358 | -3.79561 |
| OPN1SW | 0.641037 | -0.50177 | 2.959394 | 0.010994 | -2.68384 | CNNM3 | 0.597214 | 1.581028 | 2.302107 | 0.03837 | -3.79589 |
| FGF8 | -1.41129 | -0.66448 | -2.95938 | 0.010994 | -2.68385 | CLOCK | 0.589559 | 0.446105 | 2.301768 | 0.038394 | -3.79644 |
| UPK3B | 0.691444 | 0.637571 | 2.95842 | 0.011014 | -2.68553 | NEDD4L | 0.954038 | 0.799492 | 2.301587 | 0.038407 | -3.79674 |
| GGCX | 0.708706 | 2.629988 | 2.958253 | 0.011018 | -2.68582 | GPRC5D | 0.398311 | -0.88271 | 2.301034 | 0.038447 | -3.79764 |
| ZNF232 | -0.74769 | 1.8293 | -2.95795 | 0.011024 | -2.68634 | POLR3C | 0.427753 | 3.427418 | 2.300945 | 0.038453 | -3.79778 |
| BCOR | 1.384812 | 1.655461 | 2.957444 | 0.011035 | -2.68722 | HCFC1 | 0.515058 | -0.61297 | 2.30081 | 0.038463 | -3.798 |
| PHF24 | -1.23328 | -0.93252 | -2.95694 | 0.011046 | -2.6881 | DBF4B | 0.824019 | -0.97322 | 2.300561 | 0.038481 | -3.79841 |
| PDK1 | 0.732197 | 1.396106 | 2.954967 | 0.011088 | -2.69152 | FUBP1 | 0.41815 | 2.325302 | 2.299478 | 0.038558 | -3.80017 |
| STAC2 | -2.19424 | 1.911039 | -2.95384 | 0.011112 | -2.69348 | INSR | -0.9462 | 2.843713 | -2.2986 | 0.038621 | -3.80159 |
| ISCU | -0.57985 | 4.677151 | -2.94844 | 0.011228 | -2.70284 | TMEM131 | 0.524606 | 2.276734 | 2.298542 | 0.038625 | -3.80169 |
| ZNF524 | -0.66765 | 3.104404 | -2.94843 | 0.011229 | -2.70286 | BLM | 1.043317 | 1.193208 | 2.297611 | 0.038692 | -3.8032 |
| MGC12916 | 1.139281 | -1.88482 | 2.947431 | 0.01125 | -2.70459 | HYOU1 | 0.633247 | 1.879796 | 2.297303 | 0.038714 | -3.8037 |
| FBXW8 | 0.592109 | 0.703817 | 2.947289 | 0.011253 | -2.70484 | ZNF385D | -1.13328 | -0.34629 | -2.29712 | 0.038727 | -3.804 |
| LRRC73 | -1.32551 | 2.394737 | -2.94713 | 0.011257 | -2.70511 | XPO1 | 0.378037 | 2.190429 | 2.296809 | 0.03875 | -3.80451 |
| TMEM63A | 0.739335 | 0.857397 | 2.944344 | 0.011317 | -2.70995 | ZNF549 | 0.710807 | -0.44575 | 2.296215 | 0.038793 | -3.80547 |
| NTF3 | 1.454536 | 2.246441 | 2.943644 | 0.011333 | -2.71116 | TRIM11 | 0.357375 | 2.245783 | 2.29459 | 0.03891 | -3.80811 |
| id | logFC | AveExpr | t | P.Value | B | id | logFC | AveExpr | t | P.Value | B |
| MADCAM1 | -2.82376 | 0.480687 | -2.94355 | 0.011335 | -2.71133 | PTGDR | -1.29525 | 0.505626 | -2.29457 | 0.038912 | -3.80815 |
| GRTP1 | -1.68406 | 0.186394 | -2.9428 | 0.011351 | -2.71262 | SGCG | 1.530538 | -1.06382 | 2.294472 | 0.038919 | -3.80831 |
| TENT5A | 0.869155 | 2.248926 | 2.941912 | 0.01137 | -2.71416 | MUCL3 | 0.483358 | -1.8935 | 2.294306 | 0.038931 | -3.80858 |
| RAMP3 | -1.8643 | -0.39057 | -2.94183 | 0.011372 | -2.7143 | IQSEC1 | -0.68257 | 3.514522 | -2.29414 | 0.038943 | -3.80884 |
| LRFN1 | -1.11175 | 0.68985 | -2.94061 | 0.011399 | -2.71642 | GALK1 | -0.70277 | 2.53996 | -2.2941 | 0.038946 | -3.80892 |
| N4BP2L1 | -1.77855 | 0.988639 | -2.94016 | 0.011409 | -2.7172 | LINC00605 | -1.41649 | 0.125214 | -2.29263 | 0.039052 | -3.81129 |
| MIAT | -2.56325 | 1.427698 | -2.93863 | 0.011442 | -2.71985 | ZNF432 | 0.657994 | 0.71072 | 2.292526 | 0.03906 | -3.81147 |
| ESPL1 | 1.229279 | 2.519501 | 2.937659 | 0.011464 | -2.72154 | ZNF300P1 | 0.745328 | 1.517256 | 2.291978 | 0.039099 | -3.81236 |
| ATP6V1C1 | -0.44564 | 3.565916 | -2.93718 | 0.011475 | -2.72237 | PSAPL1 | 0.407128 | -2.47995 | 2.291897 | 0.039105 | -3.81249 |
| CSTB | -0.58637 | 5.120654 | -2.93573 | 0.011507 | -2.72489 | CAMKMT | 0.556201 | 0.775709 | 2.291708 | 0.039119 | -3.8128 |
| SEC11C | -0.78783 | 4.017216 | -2.9351 | 0.011521 | -2.72597 | DCTN5 | 0.44234 | 1.290953 | 2.290633 | 0.039197 | -3.81454 |
| ARMS2 | 0.606452 | -2.12337 | 2.93324 | 0.011562 | -2.7292 | ZBBX | 0.677878 | -2.04316 | 2.290456 | 0.03921 | -3.81483 |
| MT1G | -1.71129 | -0.17908 | -2.93247 | 0.011579 | -2.73053 | LINC02483 | 0.327601 | -2.42939 | 2.289778 | 0.03926 | -3.81593 |
| LINC00926 | -0.98428 | 2.306501 | -2.93181 | 0.011594 | -2.73167 | STK40 | -0.33526 | 4.917137 | -2.28969 | 0.039266 | -3.81607 |
| NCAPD2 | 0.755018 | 3.393884 | 2.931104 | 0.01161 | -2.7329 | RBM12B-AS1 | 0.589773 | -0.45089 | 2.28934 | 0.039292 | -3.81664 |
| CBLN4 | -1.91706 | -1.21121 | -2.92954 | 0.011645 | -2.7356 | BMS1P20 | 0.521805 | 0.640463 | 2.288756 | 0.039334 | -3.81759 |
| ARHGEF39 | 0.992362 | -0.03024 | 2.929245 | 0.011651 | -2.73612 | LTBP4 | -0.95227 | 4.506669 | -2.28804 | 0.039387 | -3.81876 |
| B4GALNT4 | -1.65004 | 1.907849 | -2.92888 | 0.01166 | -2.73676 | ERVV-2 | 0.542654 | -2.35669 | 2.287911 | 0.039396 | -3.81896 |
| ATP9A | 0.604873 | 3.858922 | 2.928704 | 0.011663 | -2.73706 | TPR | 0.489296 | 3.248013 | 2.287333 | 0.039438 | -3.8199 |
| SMTNL2 | -1.45955 | -1.32291 | -2.92854 | 0.011667 | -2.73734 | NANOS3 | -0.91915 | 2.151923 | -2.28671 | 0.039484 | -3.82092 |
| TEX13C | 0.519065 | -2.19529 | 2.927897 | 0.011682 | -2.73846 | CCDC170 | -1.43895 | 0.212997 | -2.28652 | 0.039498 | -3.82122 |
| SLC19A2 | 1.022766 | 2.100151 | 2.927702 | 0.011686 | -2.7388 | GAB1 | 0.848825 | -0.55821 | 2.286309 | 0.039513 | -3.82156 |
| PLTP | -1.50008 | 3.801011 | -2.92746 | 0.011691 | -2.73921 | SEMA5A | 0.683247 | 4.421418 | 2.286256 | 0.039517 | -3.82165 |
| AQP11 | 1.047729 | -0.3104 | 2.924617 | 0.011756 | -2.74414 | ZNRF4 | 0.65998 | -1.59767 | 2.286077 | 0.03953 | -3.82194 |
| id | logFC | AveExpr | t | P.Value | B | id | logFC | AveExpr | t | P.Value | B |
| HABP4 | 0.728654 | 3.916305 | 2.922193 | 0.011811 | -2.74834 | GSTT4 | 0.273103 | -2.27872 | 2.285651 | 0.039562 | -3.82263 |
| EPB41L2 | 1.181108 | 1.846352 | 2.921063 | 0.011836 | -2.7503 | INKA2 | 0.669184 | 2.137533 | 2.284976 | 0.039611 | -3.82372 |
| TEX13B | 0.491034 | -0.06317 | 2.92039 | 0.011852 | -2.75147 | INTS6 | 0.642071 | 0.49781 | 2.284149 | 0.039672 | -3.82507 |
| PDE6B | -0.62044 | -0.29862 | -2.92022 | 0.011856 | -2.75176 | EXOC2 | 0.932268 | 0.301469 | 2.283887 | 0.039692 | -3.82549 |
| ZNF391 | -1.07965 | -1.08222 | -2.92018 | 0.011857 | -2.75183 | SAMD10 | -0.64776 | 1.695164 | -2.28376 | 0.039701 | -3.8257 |
| CEL | 0.871471 | 0.277289 | 2.919579 | 0.01187 | -2.75287 | TACR1 | 0.617515 | -1.92912 | 2.283687 | 0.039706 | -3.82582 |
| PDGFA | 1.156684 | 1.717643 | 2.916484 | 0.011941 | -2.75823 | MT3 | -0.70864 | 3.216894 | -2.28313 | 0.039747 | -3.82671 |
| CLDN23 | -1.24626 | 0.509471 | -2.91603 | 0.011952 | -2.75902 | POLQ | 1.163237 | 1.135021 | 2.282913 | 0.039763 | -3.82707 |
| UBE2Q2P2 | 1.296518 | -0.53055 | 2.915834 | 0.011956 | -2.75936 | ISL1 | 0.462169 | -2.25067 | 2.282514 | 0.039793 | -3.82772 |
| CTPS2 | -0.78889 | 2.599131 | -2.91555 | 0.011963 | -2.75985 | CLN8 | 0.415217 | -2.2873 | 2.28194 | 0.039835 | -3.82865 |
| TENM4 | 1.422139 | 0.02058 | 2.915387 | 0.011967 | -2.76013 | ACADS | -0.68392 | -0.0715 | -2.28193 | 0.039836 | -3.82867 |
| PCDH7 | 1.458674 | 2.262179 | 2.915264 | 0.011969 | -2.76034 | LGI2 | -1.44555 | -0.05487 | -2.28045 | 0.039946 | -3.83107 |
| SLC44A3 | -0.68231 | 0.164402 | -2.91481 | 0.01198 | -2.76113 | SIX1 | 2.317713 | 1.021179 | 2.280394 | 0.03995 | -3.83116 |
| QSOX2 | 0.67336 | 2.382174 | 2.914393 | 0.011989 | -2.76185 | EPHA10 | -2.08774 | -0.6529 | -2.27983 | 0.039992 | -3.83208 |
| OVOL2 | -2.72667 | -0.12032 | -2.91418 | 0.011995 | -2.76223 | APOC4 | 0.387168 | 0.026177 | 2.279661 | 0.040004 | -3.83234 |
| PATE2 | 1.256508 | -1.24448 | 2.91224 | 0.012039 | -2.76558 | PEBP1 | -0.63273 | 5.19204 | -2.27919 | 0.040039 | -3.8331 |
| MYOM1 | 0.846907 | 0.168031 | 2.912129 | 0.012042 | -2.76577 | FAM124B | -1.19534 | -1.50315 | -2.27903 | 0.040051 | -3.83337 |
| AMPH | 1.078094 | 1.46819 | 2.911617 | 0.012054 | -2.76666 | PCYT1B | -0.99064 | -1.05386 | -2.279 | 0.040053 | -3.83341 |
| TTC31 | 0.525022 | 1.377103 | 2.911573 | 0.012055 | -2.76674 | ARL1 | -0.62691 | 3.169973 | -2.27806 | 0.040123 | -3.83494 |
| RIPOR3 | -2.12906 | 1.435411 | -2.91012 | 0.012089 | -2.76925 | RELT | 0.641022 | 3.662395 | 2.278018 | 0.040126 | -3.83501 |
| LRRC34 | -1.54863 | 0.17468 | -2.90911 | 0.012112 | -2.77099 | GPC1 | 0.936032 | 5.619062 | 2.277828 | 0.040141 | -3.83532 |
| DHFR | 0.737363 | -0.04381 | 2.907995 | 0.012138 | -2.77293 | PHYHIP | -1.66591 | 1.207623 | -2.27775 | 0.040146 | -3.83545 |
| INSL4 | 0.726088 | -2.21875 | 2.90658 | 0.012171 | -2.77538 | GRIK5 | -0.69717 | 0.441184 | -2.27762 | 0.040156 | -3.83566 |
| C1orf127 | 0.867673 | -0.65488 | 2.905415 | 0.012199 | -2.7774 | MPP1 | -1.10619 | -0.00993 | -2.27744 | 0.040169 | -3.83595 |
| SPATA33 | 0.860937 | 1.305059 | 2.90523 | 0.012203 | -2.77772 | CDC25C | 1.351418 | 0.07388 | 2.276646 | 0.040229 | -3.83723 |
| id | logFC | AveExpr | t | P.Value | B | id | logFC | AveExpr | t | P.Value | B |
| CAPG | -1.69554 | 3.705122 | -2.90416 | 0.012228 | -2.77957 | GPR12 | -0.86206 | -2.06629 | -2.2766 | 0.040232 | -3.83731 |
| CYTH4 | -2.35385 | -0.30154 | -2.90375 | 0.012238 | -2.78028 | C6orf141 | -1.66248 | 0.377783 | -2.27576 | 0.040295 | -3.83867 |
| ZNF223 | 0.588933 | 0.114739 | 2.901633 | 0.012288 | -2.78394 | ALG8 | 0.35455 | 4.517956 | 2.275386 | 0.040323 | -3.83927 |
| COL7A1 | -1.16042 | 4.32807 | -2.90095 | 0.012304 | -2.78513 | EYA3 | 0.331777 | -0.15354 | 2.274393 | 0.040397 | -3.84088 |
| ZNF204P | -1.88471 | 0.220891 | -2.90041 | 0.012317 | -2.78605 | CIT | 0.992375 | 1.718616 | 2.273445 | 0.040468 | -3.84241 |
| ATCAY | -3.28934 | 0.499446 | -2.89895 | 0.012351 | -2.78859 | SYT7 | -1.1985 | 1.068275 | -2.27296 | 0.040504 | -3.84319 |
| KCNE3 | -1.74586 | -0.66139 | -2.89794 | 0.012375 | -2.79034 | ETNK2 | -0.47895 | 1.775665 | -2.27198 | 0.040578 | -3.84478 |
| ARHGAP25 | -1.66444 | 1.656815 | -2.89786 | 0.012377 | -2.79047 | ZNF547 | 0.771484 | 0.068315 | 2.27172 | 0.040598 | -3.84521 |
| EPRS1 | 0.567453 | 4.462594 | 2.896251 | 0.012416 | -2.79326 | SPAG5 | 1.103515 | 3.418785 | 2.271574 | 0.040609 | -3.84545 |
| ASPM | 1.717191 | 2.366275 | 2.896205 | 0.012417 | -2.79334 | ALPK1 | 0.425966 | 1.249886 | 2.270783 | 0.040669 | -3.84673 |
| NFIX | 0.721843 | 2.39161 | 2.896122 | 0.012419 | -2.79348 | GRIN3B | -1.9103 | 1.648195 | -2.27078 | 0.040669 | -3.84674 |
| CAT | -0.70831 | 3.53181 | -2.89521 | 0.012441 | -2.79505 | CCNI2 | -0.87446 | -1.64767 | -2.27071 | 0.040674 | -3.84685 |
| C2CD4B | -1.90472 | -0.30968 | -2.895 | 0.012446 | -2.79543 | HLA-F | -1.04204 | 2.372712 | -2.27017 | 0.040714 | -3.84771 |
| LBX2 | -1.05684 | -0.54977 | -2.89446 | 0.012459 | -2.79636 | ANKRD17 | 0.350157 | 1.841414 | 2.26998 | 0.040729 | -3.84802 |
| EPOR | -1.25618 | 1.90635 | -2.89347 | 0.012482 | -2.79808 | C1GALT1 | 0.851227 | 0.705663 | 2.269679 | 0.040752 | -3.84851 |
| CHD1L | 0.709132 | 2.376974 | 2.893451 | 0.012483 | -2.7981 | ALG6 | 0.298973 | 2.757688 | 2.269614 | 0.040757 | -3.84862 |
| LHX5 | -0.8213 | -1.87554 | -2.89269 | 0.012501 | -2.79942 | TNNC2 | -1.12997 | 1.101649 | -2.26891 | 0.04081 | -3.84976 |
| WWC3 | 0.576042 | 3.687107 | 2.892625 | 0.012503 | -2.79953 | KIAA1549L | 1.332398 | 0.261659 | 2.268717 | 0.040825 | -3.85007 |
| POSTN | 3.113822 | 1.690847 | 2.890171 | 0.012562 | -2.80378 | SHH | 0.667906 | -0.66691 | 2.268418 | 0.040847 | -3.85055 |
| HS3ST1 | -2.08233 | 0.181985 | -2.89012 | 0.012563 | -2.80386 | HSD17B14 | -1.22266 | 1.565395 | -2.26809 | 0.040872 | -3.85109 |
| NACC2 | 0.737812 | 3.417159 | 2.888145 | 0.012611 | -2.80728 | POGLUT2 | 0.63983 | 1.970404 | 2.267555 | 0.040913 | -3.85195 |
| CNKSR3 | 1.044813 | 1.755742 | 2.887586 | 0.012625 | -2.80825 | LHX4 | 1.429796 | -1.71791 | 2.267244 | 0.040936 | -3.85245 |
| RGS19 | -0.62704 | 2.57089 | -2.8874 | 0.012629 | -2.80857 | DNAJB9 | -0.79406 | 3.525761 | -2.26686 | 0.040965 | -3.85307 |
| GPATCH2 | 0.639414 | 2.213017 | 2.886745 | 0.012645 | -2.8097 | ANKRD19P | 2.352739 | -1.61478 | 2.266834 | 0.040967 | -3.85311 |
| CBX7 | -0.86583 | 2.910063 | -2.88659 | 0.012649 | -2.80997 | MYBL2 | 1.429867 | 1.399883 | 2.266779 | 0.040972 | -3.8532 |
| id | logFC | AveExpr | t | P.Value | B | id | logFC | AveExpr | t | P.Value | B |
| ANKRD26P1 | 0.677147 | -1.79667 | 2.88649 | 0.012651 | -2.81014 | CENPA | 1.619504 | -0.43784 | 2.266544 | 0.040989 | -3.85358 |
| PLA2R1 | -1.95245 | 0.757741 | -2.88605 | 0.012662 | -2.8109 | C3orf22 | 0.724876 | -0.09521 | 2.266509 | 0.040992 | -3.85364 |
| PRRG3 | -1.96318 | -0.41857 | -2.8858 | 0.012668 | -2.81133 | NDUFA5 | -0.50563 | 2.872775 | -2.26533 | 0.041082 | -3.85554 |
| SLC1A4 | 0.917842 | 1.251242 | 2.883132 | 0.012733 | -2.81595 | AK8 | -0.531 | 0.557217 | -2.2644 | 0.041152 | -3.85705 |
| MMD2 | -0.9984 | -1.53185 | -2.87859 | 0.012845 | -2.82381 | TEX14 | 0.385769 | -2.36136 | 2.264257 | 0.041163 | -3.85728 |
| FOXA2 | -1.06921 | -0.45963 | -2.8767 | 0.012892 | -2.82708 | ZBTB18 | 0.439023 | -2.54513 | 2.263577 | 0.041215 | -3.85838 |
| ZMYM1 | 0.583399 | 1.06424 | 2.875915 | 0.012911 | -2.82842 | CSPP1 | 0.535732 | 0.971746 | 2.263149 | 0.041248 | -3.85907 |
| LL22NC03-63E9.3 | 0.787493 | -0.75593 | 2.875736 | 0.012916 | -2.82873 | DCAF5 | 0.518488 | 1.982438 | 2.262906 | 0.041267 | -3.85947 |
| JAG1 | 1.110867 | 0.896769 | 2.874889 | 0.012937 | -2.8302 | CYP19A1 | 0.87293 | -1.99705 | 2.262785 | 0.041276 | -3.85966 |
| DHH | -2.31755 | -0.28633 | -2.87471 | 0.012941 | -2.8305 | E2F1 | 1.201629 | 1.540841 | 2.262702 | 0.041282 | -3.8598 |
| POLB | 0.578831 | -1.89908 | 2.874507 | 0.012946 | -2.83086 | CHRD | -1.03076 | 3.182488 | -2.26219 | 0.041322 | -3.86063 |
| UBASH3B | 1.823438 | 0.657065 | 2.873621 | 0.012968 | -2.83239 | CEMIP2 | 0.852464 | 2.393654 | 2.261759 | 0.041354 | -3.86132 |
| UBAP2L | 0.788057 | 2.316861 | 2.873368 | 0.012975 | -2.83283 | GNRH2 | 0.663761 | -0.47607 | 2.261423 | 0.04138 | -3.86186 |
| SERPING1 | -2.50239 | 2.714522 | -2.87159 | 0.013019 | -2.8359 | KDM4C | 0.39753 | 1.147548 | 2.26101 | 0.041412 | -3.86253 |
| KLHL15 | 1.286851 | -0.98202 | 2.871534 | 0.013021 | -2.836 | TRIM23 | 0.350094 | -2.28451 | 2.260955 | 0.041416 | -3.86262 |
| CYP2C18 | 0.638286 | -2.23113 | 2.871141 | 0.01303 | -2.83668 | TSSK4 | 0.643334 | -1.28718 | 2.259949 | 0.041493 | -3.86424 |
| MAL | -2.70294 | 1.005769 | -2.87094 | 0.013036 | -2.83703 | SMARCA4 | 0.488819 | 5.376406 | 2.259898 | 0.041497 | -3.86433 |
| SAT1 | -1.25048 | 5.303464 | -2.86673 | 0.013141 | -2.84429 | CASQ1 | 1.073289 | -0.99268 | 2.259632 | 0.041518 | -3.86476 |
| LAG3 | -1.04414 | 3.046395 | -2.86546 | 0.013174 | -2.84649 | PIGY | -0.42939 | 3.905211 | -2.25891 | 0.041573 | -3.86592 |
| DTL | 1.557075 | 1.644416 | 2.864982 | 0.013186 | -2.84732 | SPANXA1 | 1.816565 | -1.70179 | 2.258801 | 0.041582 | -3.8661 |
| KCNK17 | -2.92119 | 0.343229 | -2.86476 | 0.013191 | -2.84771 | UGGT2 | 0.524759 | 3.830226 | 2.258253 | 0.041624 | -3.86698 |
| SULT4A1 | -1.42985 | 2.000996 | -2.86214 | 0.013258 | -2.85223 | IL5 | 0.627263 | -1.8255 | 2.2571 | 0.041713 | -3.86884 |
| KIAA0040 | -1.69752 | 1.207046 | -2.86199 | 0.013262 | -2.85248 | STX5 | -0.36393 | 3.821074 | -2.25709 | 0.041713 | -3.86886 |
| NOTCH1 | 0.754375 | 0.794897 | 2.860269 | 0.013306 | -2.85546 | RAP1A | -0.39979 | 3.926683 | -2.25665 | 0.041748 | -3.86958 |
| id | logFC | AveExpr | t | P.Value | B | id | logFC | AveExpr | t | P.Value | B |
| DNAH9 | 1.066193 | -1.52288 | 2.859939 | 0.013314 | -2.85602 | WASL | -0.43986 | 3.801547 | -2.25601 | 0.041797 | -3.87061 |
| PIP4P2 | -0.47762 | 2.97363 | -2.85962 | 0.013322 | -2.85657 | LARS2 | 0.424636 | 2.53295 | 2.255162 | 0.041863 | -3.87197 |
| HPN-AS1 | 0.560116 | -0.23558 | 2.859368 | 0.013329 | -2.85701 | FLT3LG | -0.57293 | 1.189309 | -2.25447 | 0.041916 | -3.87309 |
| PRDM10 | 0.497431 | 1.070648 | 2.859336 | 0.01333 | -2.85707 | SH2D4B | 0.591028 | -2.01465 | 2.254468 | 0.041917 | -3.87309 |
| ADGRG1 | -2.21703 | 3.009719 | -2.85877 | 0.013344 | -2.85804 | ANP32A | 0.67568 | 0.310428 | 2.253884 | 0.041962 | -3.87404 |
| CLSTN3 | -0.59506 | 3.882129 | -2.85733 | 0.013381 | -2.86053 | SLC7A9 | -0.78312 | -1.9846 | -2.25299 | 0.042031 | -3.87548 |
| ZNF430 | 0.664526 | -0.2309 | 2.856969 | 0.013391 | -2.86115 | NPM2 | -1.07064 | 1.898352 | -2.25242 | 0.042076 | -3.8764 |
| NUDT8 | -0.62215 | 2.429705 | -2.85664 | 0.013399 | -2.86172 | NUDT16 | -0.75267 | 1.810024 | -2.2519 | 0.042116 | -3.87724 |
| CAD | 0.726694 | 3.70387 | 2.855881 | 0.013419 | -2.86303 | AP1S3 | 1.131186 | 0.418305 | 2.251317 | 0.042162 | -3.87818 |
| GSTA3 | 0.707222 | -1.0479 | 2.855603 | 0.013426 | -2.86351 | BCO2 | -1.25611 | 0.48225 | -2.25084 | 0.042199 | -3.87895 |
| TCEAL1 | -0.76897 | 2.903887 | -2.85153 | 0.013531 | -2.87054 | ACBD6 | 0.510638 | 4.370543 | 2.250666 | 0.042213 | -3.87923 |
| KHDC4 | 0.650797 | 1.375061 | 2.851052 | 0.013544 | -2.87137 | USP34 | 0.424787 | 2.742512 | 2.249954 | 0.042268 | -3.88038 |
| PRB4 | 0.611471 | -0.58809 | 2.850549 | 0.013557 | -2.87224 | CGAS | -0.99548 | 1.28542 | -2.2499 | 0.042272 | -3.88046 |
| GIMAP2 | -1.7193 | -0.69218 | -2.85053 | 0.013557 | -2.87226 | MORC1 | 1.152805 | -1.23279 | 2.249815 | 0.042279 | -3.8806 |
| B3GALT6 | 0.571394 | 3.846345 | 2.84839 | 0.013613 | -2.87596 | TEAD4 | 0.646685 | 2.700224 | 2.249753 | 0.042284 | -3.8807 |
| ACRV1 | 0.606343 | -2.00221 | 2.848281 | 0.013616 | -2.87615 | KLF5 | 1.217619 | 0.709876 | 2.249743 | 0.042285 | -3.88072 |
| NBPF6 | 0.712899 | -0.58886 | 2.847966 | 0.013624 | -2.87669 | GIN1 | -0.42318 | 0.364359 | -2.24916 | 0.042331 | -3.88166 |
| DYNC1LI1 | 0.538444 | 1.037126 | 2.84747 | 0.013637 | -2.87755 | CD320 | 0.382352 | 4.298171 | 2.248596 | 0.042375 | -3.88257 |
| RAB11FIP5 | 0.80111 | 3.565016 | 2.846585 | 0.013661 | -2.87908 | DUSP26 | -1.05443 | 2.089845 | -2.24853 | 0.04238 | -3.88268 |
| ZNF230 | 0.459297 | -0.13944 | 2.844958 | 0.013703 | -2.88188 | ZNF668 | 0.48919 | 3.990968 | 2.248133 | 0.042411 | -3.88331 |
| MT1M | -1.57055 | -0.11896 | -2.84369 | 0.013737 | -2.88408 | TSHB | 0.405539 | -0.03651 | 2.247735 | 0.042442 | -3.88396 |
| SHOX2 | 2.132354 | -0.08918 | 2.843032 | 0.013754 | -2.88521 | FAM223B | 0.490249 | -1.19377 | 2.247595 | 0.042453 | -3.88418 |
| FGF21 | 0.496051 | -1.38531 | 2.842712 | 0.013763 | -2.88576 | CRISPLD1 | 1.19618 | -1.66729 | 2.246793 | 0.042516 | -3.88547 |
| MORC4 | 0.587071 | 2.930419 | 2.841437 | 0.013796 | -2.88796 | SLC16A6 | -1.47194 | -1.33135 | -2.24661 | 0.04253 | -3.88577 |
| SLC15A3 | -1.62327 | 3.240691 | -2.84084 | 0.013812 | -2.88898 | NBPF22P | 0.762419 | -1.52259 | 2.246513 | 0.042538 | -3.88592 |
| id | logFC | AveExpr | t | P.Value | B | id | logFC | AveExpr | t | P.Value | B |
| COLCA2 | -1.27753 | -1.48636 | -2.84069 | 0.013816 | -2.88925 | SERP2 | -0.90923 | 2.392128 | -2.24645 | 0.042543 | -3.88603 |
| GOLGA6L5P | 0.531746 | -1.56285 | 2.84057 | 0.013819 | -2.88945 | CCDC15 | 0.66648 | -0.38474 | 2.246247 | 0.042559 | -3.88635 |
| RASGRF2 | -1.65489 | -0.33577 | -2.84044 | 0.013823 | -2.88967 | SPIRE2 | -0.61872 | 2.183721 | -2.24608 | 0.042572 | -3.88662 |
| NTM | 1.759616 | 0.369007 | 2.839465 | 0.013849 | -2.89136 | ZNF200 | 0.407243 | 1.643131 | 2.246058 | 0.042574 | -3.88666 |
| ZCCHC2 | 0.837929 | 0.83241 | 2.837104 | 0.013912 | -2.89543 | NIT1 | 0.344645 | 2.869401 | 2.246024 | 0.042577 | -3.88671 |
| SYNGR3 | -1.39042 | 1.643823 | -2.83688 | 0.013918 | -2.89582 | MSL3 | 0.364174 | -2.29402 | 2.245535 | 0.042615 | -3.8875 |
| STK11IP | 0.637707 | 4.270695 | 2.836315 | 0.013933 | -2.89679 | SLC25A44 | 0.441255 | 3.488349 | 2.245339 | 0.042631 | -3.88782 |
| VEGFB | -0.62104 | 4.795476 | -2.83439 | 0.013985 | -2.90011 | FZD4 | 0.890981 | 3.03086 | 2.245208 | 0.042641 | -3.88803 |
| LINC00200 | 0.507819 | -2.15622 | 2.832952 | 0.014023 | -2.90259 | TBXT | 0.481359 | -2.05894 | 2.245118 | 0.042648 | -3.88817 |
| HOXB2 | 2.660944 | 1.363138 | 2.832235 | 0.014043 | -2.90383 | DRD4 | -1.36784 | 2.871592 | -2.24476 | 0.042676 | -3.88874 |
| PNPLA8 | -0.55316 | 3.06128 | -2.83217 | 0.014044 | -2.90394 | VPS33B | 0.423953 | 2.154403 | 2.244494 | 0.042697 | -3.88918 |
| ETNPPL | 0.794967 | -2.11549 | 2.831656 | 0.014058 | -2.90482 | SPATA41 | -0.87314 | -0.9097 | -2.24403 | 0.042734 | -3.88992 |
| NEK5 | 0.605855 | -2.30806 | 2.830606 | 0.014087 | -2.90663 | CASP8AP2 | 0.445792 | 0.228092 | 2.243745 | 0.042757 | -3.89038 |
| TRAM1L1 | 0.766771 | -0.21879 | 2.830497 | 0.01409 | -2.90682 | KLK6 | -2.46438 | 0.121369 | -2.24315 | 0.042804 | -3.89134 |
| TOR3A | 0.5398 | 3.023676 | 2.829545 | 0.014115 | -2.90846 | GAL3ST1 | -0.719 | -0.62151 | -2.24302 | 0.042814 | -3.89155 |
| RNF157 | -1.65359 | 0.280392 | -2.82897 | 0.014131 | -2.90945 | AADACL3 | 0.375391 | -0.52433 | 2.242914 | 0.042822 | -3.89172 |
| KIF16B | 0.593524 | 0.191257 | 2.828931 | 0.014132 | -2.90952 | CKAP5 | 0.436536 | 2.905347 | 2.242702 | 0.042839 | -3.89206 |
| URB2 | 0.687141 | 1.028539 | 2.828152 | 0.014153 | -2.91086 | LINC01634 | 0.347013 | -2.47401 | 2.2421 | 0.042887 | -3.89303 |
| CPNE5 | -1.53226 | 2.61671 | -2.8267 | 0.014193 | -2.91336 | DCAF8 | 0.478052 | 1.542943 | 2.241163 | 0.042961 | -3.89454 |
| PPP1R1B | -2.53325 | -0.18647 | -2.82657 | 0.014196 | -2.91359 | ADRA2C | -1.34017 | 4.88669 | -2.24038 | 0.043023 | -3.8958 |
| CBFA2T2 | 0.612444 | 0.651234 | 2.825708 | 0.01422 | -2.91508 | MAN2B1 | 0.617431 | 3.479526 | 2.24037 | 0.043024 | -3.89582 |
| NT5M | -0.78937 | 1.698458 | -2.82566 | 0.014221 | -2.91515 | WDHD1 | 0.665015 | 1.783781 | 2.240337 | 0.043027 | -3.89587 |
| LUZP1 | 0.835357 | 1.762538 | 2.825617 | 0.014222 | -2.91523 | TACC2 | 0.473537 | 1.256277 | 2.240214 | 0.043037 | -3.89607 |
| MLN | 0.659787 | -1.59353 | 2.824964 | 0.01424 | -2.91636 | LMCD1 | 1.089218 | 3.576436 | 2.240205 | 0.043037 | -3.89608 |
| DNM1P46 | -1.49612 | 1.682958 | -2.82357 | 0.014278 | -2.91877 | ANKMY1 | 0.602629 | 2.712999 | 2.240034 | 0.043051 | -3.89636 |
| id | logFC | AveExpr | t | P.Value | B | id | logFC | AveExpr | t | P.Value | B |
| GJA4 | -2.0767 | 0.554751 | -2.82318 | 0.014289 | -2.91944 | IMMT | 0.449926 | 1.760752 | 2.239762 | 0.043073 | -3.8968 |
| LRP1B | 2.492683 | -0.72707 | 2.822799 | 0.0143 | -2.92009 | MYBPC2 | -1.21847 | -0.99817 | -2.2396 | 0.043086 | -3.89706 |
| KCNJ13 | 0.897171 | -0.28032 | 2.822716 | 0.014302 | -2.92023 | TMEM250 | 0.523507 | 2.339518 | 2.239331 | 0.043107 | -3.89749 |
| EPS8L1 | -2.02831 | 2.406574 | -2.82267 | 0.014303 | -2.92031 | HOXA1 | 0.625246 | -2.37147 | 2.239184 | 0.043119 | -3.89773 |
| OR2W5 | 0.779198 | -1.78935 | 2.822235 | 0.014315 | -2.92106 | SPRY3 | 0.355926 | 0.095057 | 2.239124 | 0.043123 | -3.89782 |
| ALS2 | 0.531654 | 1.16282 | 2.822025 | 0.014321 | -2.92142 | SETDB1 | 0.592876 | 3.409409 | 2.238994 | 0.043134 | -3.89803 |
| ZNF175 | 0.580177 | 1.290968 | 2.82156 | 0.014334 | -2.92222 | TCEA3 | -1.42083 | 1.617828 | -2.23786 | 0.043224 | -3.89986 |
| PPP6R2 | 0.481083 | 2.810684 | 2.820231 | 0.01437 | -2.92451 | CDCA5 | 1.057174 | 2.764791 | 2.237088 | 0.043286 | -3.9011 |
| OR12D2 | 0.674521 | -2.22116 | 2.819906 | 0.014379 | -2.92507 | RNASEH2B | -0.70725 | 2.096838 | -2.23675 | 0.043313 | -3.90164 |
| CITED4 | -0.87751 | 4.799745 | -2.81915 | 0.0144 | -2.92638 | DDIT4L | -1.42655 | 1.255343 | -2.23646 | 0.043337 | -3.90212 |
| MARCHF9 | -0.86408 | 3.501317 | -2.81802 | 0.014431 | -2.92832 | FAM83B | -1.1813 | -1.40898 | -2.23537 | 0.043424 | -3.90387 |
| TFCP2L1 | -1.81315 | -0.76492 | -2.8173 | 0.014451 | -2.92956 | CACNA2D1 | 0.814549 | 1.56119 | 2.235355 | 0.043425 | -3.90389 |
| SELENBP1 | 1.707002 | 3.463259 | 2.816999 | 0.01446 | -2.93008 | SHC3 | 0.840315 | 1.873075 | 2.234694 | 0.043478 | -3.90495 |
| HLF | -2.13476 | -0.38973 | -2.8155 | 0.014501 | -2.93265 | MAP1LC3A | -0.46694 | 2.8013 | -2.23419 | 0.043519 | -3.90577 |
| MEIG1 | 0.6423 | -1.96969 | 2.815453 | 0.014503 | -2.93274 | JAML | -2.28193 | -0.29377 | -2.23395 | 0.043538 | -3.90615 |
| ALOX12B | 0.477834 | -0.35171 | 2.814858 | 0.014519 | -2.93377 | NPAS2 | 0.825283 | 1.39253 | 2.233927 | 0.043539 | -3.90619 |
| NOL4L | 0.558893 | -0.43202 | 2.814562 | 0.014528 | -2.93428 | CEP152 | 0.547314 | 0.609791 | 2.233551 | 0.04357 | -3.90679 |
| PYGB | 0.412299 | 4.825094 | 2.814264 | 0.014536 | -2.93479 | OBSL1 | 0.591474 | 4.047345 | 2.233162 | 0.043601 | -3.90742 |
| RASSF3 | 0.80887 | 0.206074 | 2.814073 | 0.014541 | -2.93512 | UNC93B1 | -0.70394 | 2.22779 | -2.23296 | 0.043617 | -3.90774 |
| MAGED1 | 0.559695 | 5.006293 | 2.812825 | 0.014576 | -2.93727 | NPFFR1 | 0.729086 | -1.05778 | 2.232784 | 0.043632 | -3.90802 |
| SPTAN1 | 0.554318 | 3.079604 | 2.81222 | 0.014593 | -2.93831 | SNORA74B | 0.485665 | 1.44219 | 2.232659 | 0.043642 | -3.90822 |
| ZNF174 | 0.721252 | 0.710231 | 2.811295 | 0.014619 | -2.9399 | PNPT1 | 0.447925 | 2.288207 | 2.232326 | 0.043668 | -3.90876 |
| PCP4L1 | -2.39641 | -0.29786 | -2.81074 | 0.014635 | -2.94087 | FNDC4 | -0.68223 | 1.913433 | -2.23163 | 0.043725 | -3.90989 |
| ARSH | 0.654884 | -2.00044 | 2.809342 | 0.014674 | -2.94327 | BARD1 | 0.955235 | 1.738371 | 2.231348 | 0.043747 | -3.91033 |
| HTRA4 | -1.24783 | -0.50921 | -2.80911 | 0.014681 | -2.94367 | RRP15 | 0.666413 | 0.725049 | 2.230806 | 0.043791 | -3.9112 |
| id | logFC | AveExpr | t | P.Value | B | id | logFC | AveExpr | t | P.Value | B |
| STRA6 | -1.5333 | 0.589449 | -2.80774 | 0.014719 | -2.94602 | ARHGAP8 | -1.16418 | -1.29254 | -2.23078 | 0.043794 | -3.91125 |
| C5orf63 | 0.532922 | -0.52912 | 2.807127 | 0.014736 | -2.94708 | GAL3ST3 | 0.425661 | -2.17799 | 2.230461 | 0.043819 | -3.91176 |
| ACTL8 | 0.743564 | -0.398 | 2.80656 | 0.014753 | -2.94806 | PFDN6 | -0.37851 | 3.092196 | -2.23029 | 0.043833 | -3.91204 |
| TDRD3 | 0.427274 | -1.97991 | 2.805373 | 0.014786 | -2.9501 | RGS20 | 1.348339 | 1.519223 | 2.230105 | 0.043848 | -3.91233 |
| SERTAD2 | 0.826445 | 2.15877 | 2.805196 | 0.014791 | -2.9504 | CERS5 | 0.38385 | 4.710204 | 2.229942 | 0.043861 | -3.91259 |
| ETV7 | -1.89083 | -0.08523 | -2.8042 | 0.01482 | -2.95212 | NSD2 | 0.566258 | 1.400411 | 2.229259 | 0.043916 | -3.91369 |
| PRAMEF19 | 0.546436 | -2.24917 | 2.802725 | 0.014862 | -2.95465 | TENM3-AS1 | 1.272411 | -0.53331 | 2.22901 | 0.043937 | -3.91409 |
| SCAMP3 | 0.410214 | 5.819914 | 2.802305 | 0.014874 | -2.95538 | ARID2 | 0.500623 | 1.852108 | 2.228878 | 0.043947 | -3.9143 |
| LYG2 | 0.793346 | -2.02498 | 2.802081 | 0.01488 | -2.95576 | P2RX1 | 1.008131 | -0.51153 | 2.228691 | 0.043963 | -3.9146 |
| WAC-AS1 | 0.989815 | -1.50968 | 2.79946 | 0.014955 | -2.96027 | INTS6L | 0.87353 | 0.111049 | 2.228147 | 0.044007 | -3.91548 |
| CADM4 | -1.24645 | 1.191304 | -2.79834 | 0.014987 | -2.9622 | ADAMTS13 | 0.845292 | 3.010565 | 2.227576 | 0.044053 | -3.91639 |
| ACACB | -1.19094 | 1.743253 | -2.7972 | 0.01502 | -2.96417 | MS4A5 | 0.551321 | -1.29626 | 2.22715 | 0.044088 | -3.91708 |
| C1orf210 | 0.889592 | -1.56905 | 2.795851 | 0.015059 | -2.96648 | UGT2A1 | 0.300082 | -2.28388 | 2.226753 | 0.04412 | -3.91771 |
| RBP4 | -1.84942 | 1.983432 | -2.79534 | 0.015074 | -2.96735 | NDUFB10 | -0.34791 | 5.150369 | -2.22637 | 0.044151 | -3.91833 |
| IFIH1 | -1.98505 | 1.691271 | -2.7951 | 0.015081 | -2.96778 | TPRX1 | 0.547804 | -0.54495 | 2.226364 | 0.044152 | -3.91834 |
| ATP2B3 | -1.22299 | 0.966469 | -2.79509 | 0.015081 | -2.96779 | NPAT | 0.586422 | -0.96363 | 2.225749 | 0.044202 | -3.91933 |
| RNF144A | -1.82448 | -0.3852 | -2.7948 | 0.015089 | -2.96828 | CDH2 | 1.621537 | 4.895208 | 2.225606 | 0.044214 | -3.91956 |
| NXNL2 | -2.05582 | 1.546231 | -2.79352 | 0.015127 | -2.97049 | LRRC10 | 0.798721 | -0.34881 | 2.224448 | 0.044308 | -3.92142 |
| KIF15 | 1.394375 | 1.278144 | 2.79305 | 0.01514 | -2.9713 | ZC3H7A | 0.363627 | 3.576934 | 2.223653 | 0.044373 | -3.92269 |
| HMGCR | 0.544432 | 3.130002 | 2.791305 | 0.015191 | -2.9743 | HUS1B | 0.601464 | -1.54099 | 2.223461 | 0.044389 | -3.923 |
| RASD2 | -0.8853 | 0.658778 | -2.79121 | 0.015194 | -2.97446 | FAM151B | 0.437295 | -1.10413 | 2.222214 | 0.044491 | -3.925 |
| KRTAP9-4 | 0.797577 | -2.06122 | 2.790774 | 0.015206 | -2.97521 | GPR31 | 0.879751 | -1.34393 | 2.222008 | 0.044508 | -3.92533 |
| HHAT | 0.683309 | 1.82492 | 2.78988 | 0.015233 | -2.97675 | CHRNB2 | -0.96057 | -0.64901 | -2.22195 | 0.044513 | -3.92542 |
| RPH3AL | -1.20547 | 1.573123 | -2.78876 | 0.015265 | -2.97868 | TIMM10B | -0.33873 | 3.853312 | -2.22119 | 0.044575 | -3.92664 |
| THUMPD2 | 0.490216 | 1.383329 | 2.787899 | 0.015291 | -2.98015 | ZNF438 | -0.65655 | 2.178634 | -2.22097 | 0.044593 | -3.92699 |
| id | logFC | AveExpr | t | P.Value | B | id | logFC | AveExpr | t | P.Value | B |
| SPSB4 | 1.075595 | 2.808632 | 2.786854 | 0.015321 | -2.98195 | CDCA2 | 1.195054 | 1.926325 | 2.220803 | 0.044607 | -3.92726 |
| CLHC1 | 0.867789 | 0.300758 | 2.785162 | 0.015371 | -2.98486 | UBE2L6 | -0.8606 | 3.084019 | -2.22034 | 0.044645 | -3.92801 |
| HOXA6 | 2.147499 | 3.865873 | 2.784732 | 0.015384 | -2.9856 | USP7 | 0.426563 | 0.734856 | 2.219817 | 0.044688 | -3.92884 |
| FAM43A | 0.766869 | 3.879083 | 2.784036 | 0.015404 | -2.98679 | MTG1 | 0.696776 | 1.422335 | 2.219408 | 0.044722 | -3.9295 |
| MTCL1 | 1.431741 | 0.197168 | 2.783319 | 0.015425 | -2.98803 | KIF13B | -0.35719 | 2.34987 | -2.2193 | 0.044731 | -3.92968 |
| RARRES1 | -2.29428 | 1.941456 | -2.78167 | 0.015474 | -2.99086 | ZNF276 | 0.665386 | 0.24965 | 2.218456 | 0.044801 | -3.93103 |
| LIMCH1 | 1.236764 | 2.4907 | 2.781645 | 0.015475 | -2.9909 | IGFBP4 | -1.5457 | 3.656968 | -2.21745 | 0.044884 | -3.93263 |
| ERCC6 | 0.559278 | 1.291401 | 2.781392 | 0.015483 | -2.99134 | PRAMEF7 | -1.2264 | -1.35965 | -2.21727 | 0.044899 | -3.93293 |
| KRTAP7-1 | 1.327653 | -1.6888 | 2.780595 | 0.015506 | -2.99271 | HTATSF1P2 | 0.689439 | -0.62019 | 2.217093 | 0.044913 | -3.93321 |
| DACT1 | 1.124968 | 2.826821 | 2.780175 | 0.015519 | -2.99343 | LGI1 | 0.912744 | -2.03629 | 2.217046 | 0.044917 | -3.93329 |
| ST6GALNAC3 | -1.39017 | 0.067201 | -2.78002 | 0.015523 | -2.99369 | TMEM170B | -0.72143 | -0.00503 | -2.21705 | 0.044917 | -3.93329 |
| LMTK3 | -1.92344 | 1.426249 | -2.77891 | 0.015556 | -2.9956 | ZNF367 | 0.967017 | -0.83251 | 2.215485 | 0.045047 | -3.93579 |
| DNAJC11 | 0.397796 | 2.620891 | 2.778648 | 0.015564 | -2.99605 | GLCE | 1.112517 | -0.30332 | 2.2154 | 0.045054 | -3.93593 |
| ANOS1 | 1.665597 | 1.19449 | 2.778551 | 0.015567 | -2.99622 | KRTAP5-9 | 0.644884 | -1.9469 | 2.215062 | 0.045082 | -3.93647 |
| MINK1 | 0.614774 | 0.756232 | 2.778187 | 0.015578 | -2.99684 | PIDD1 | 0.550328 | 4.475212 | 2.214967 | 0.04509 | -3.93662 |
| CENPL | 0.902821 | -0.20572 | 2.776568 | 0.015627 | -2.99963 | MTIF2 | 0.586729 | 1.743311 | 2.214452 | 0.045133 | -3.93745 |
| VPS45 | 0.567131 | 2.240605 | 2.776393 | 0.015632 | -2.99993 | MCM10 | 1.134879 | -0.77111 | 2.213361 | 0.045224 | -3.93919 |
| PLA2G6 | 0.796221 | -0.8401 | 2.775154 | 0.015669 | -3.00205 | HPCA | -1.66363 | -0.88814 | -2.21333 | 0.045226 | -3.93925 |
| TNFSF10 | -2.10208 | 1.666399 | -2.77457 | 0.015687 | -3.00306 | WDR27 | 0.74711 | 0.847214 | 2.213225 | 0.045235 | -3.93941 |
| ARL6 | -0.85287 | -1.39208 | -2.77249 | 0.015749 | -3.00663 | OR51V1 | 0.408265 | -2.12747 | 2.213207 | 0.045236 | -3.93944 |
| RAMP2 | -0.61932 | -1.80143 | -2.77249 | 0.015749 | -3.00663 | CELA2B | 0.455248 | -0.30854 | 2.212484 | 0.045297 | -3.9406 |
| SNX10 | -1.67278 | -1.21539 | -2.77142 | 0.015782 | -3.00847 | SFRP5 | -1.77757 | -0.73676 | -2.21242 | 0.045302 | -3.9407 |
| KIF12 | -1.63794 | -0.83356 | -2.77076 | 0.015802 | -3.0096 | ACYP2 | -0.69461 | 2.366036 | -2.21232 | 0.045311 | -3.94087 |
| ZNF221 | 0.675722 | -0.46218 | 2.770488 | 0.01581 | -3.01007 | PRDX6 | 0.315636 | 5.493812 | 2.212097 | 0.045329 | -3.94122 |
| id | logFC | AveExpr | t | P.Value | B | id | logFC | AveExpr | t | P.Value | B |
| SLC1A2 | -1.46268 | -0.10685 | -2.77036 | 0.015814 | -3.01029 | NRADDP | -1.11016 | -1.44172 | -2.21185 | 0.045349 | -3.94161 |
| GNAO1 | -1.44008 | 0.152068 | -2.77013 | 0.015821 | -3.01068 | TARS2 | 0.379597 | 2.616869 | 2.211819 | 0.045352 | -3.94166 |
| ITGA8 | -1.71643 | -0.00316 | -2.76967 | 0.015835 | -3.01146 | EXOC3L1 | -0.6183 | 1.289687 | -2.2116 | 0.045371 | -3.94202 |
| BSN | -1.12023 | -0.3207 | -2.76951 | 0.01584 | -3.01175 | ERCC8 | 0.544775 | -0.42844 | 2.211242 | 0.0454 | -3.94259 |
| COPS2 | -0.46548 | 3.464879 | -2.76946 | 0.015841 | -3.01183 | PPM1B | 0.383965 | 2.33126 | 2.211148 | 0.045408 | -3.94274 |
| LCTL | 1.236265 | -0.8235 | 2.768843 | 0.01586 | -3.01289 | WEE1 | 0.627186 | 2.186805 | 2.210563 | 0.045457 | -3.94368 |
| ABI3 | -1.75413 | 0.498382 | -2.7673 | 0.015907 | -3.01555 | ANKK1 | -0.94341 | -1.09809 | -2.21024 | 0.045484 | -3.94419 |
| ZNF688 | -0.48381 | 3.101678 | -2.76624 | 0.015939 | -3.01735 | C8orf37 | 0.490489 | 0.581914 | 2.21017 | 0.04549 | -3.9443 |
| CEP170 | 0.755281 | 1.828585 | 2.766133 | 0.015942 | -3.01754 | FLVCR1 | 0.565115 | 1.050813 | 2.209837 | 0.045518 | -3.94484 |
| FURIN | 1.10737 | -0.2392 | 2.764514 | 0.015992 | -3.02032 | COL16A1 | 1.295369 | 4.027065 | 2.209304 | 0.045563 | -3.94569 |
| CAMK2D | 0.649113 | 2.832415 | 2.760959 | 0.016101 | -3.02642 | SNX21 | -0.67638 | 3.517685 | -2.20901 | 0.045588 | -3.94616 |
| CD209 | -1.51136 | -1.35398 | -2.75687 | 0.016228 | -3.03344 | ZNF256 | 0.44239 | 1.774847 | 2.208728 | 0.045611 | -3.94661 |
| CHI3L1 | -4.36638 | 4.528712 | -2.75665 | 0.016235 | -3.03381 | DUSP10 | 1.126398 | 0.307156 | 2.208198 | 0.045656 | -3.94746 |
| PAQR5 | -1.95547 | 0.34837 | -2.75581 | 0.016261 | -3.03525 | LYPD3 | -1.43218 | -0.24847 | -2.20812 | 0.045662 | -3.94758 |
| SS18L1 | 0.809368 | 0.2107 | 2.754812 | 0.016292 | -3.03697 | KCNK3 | -1.16715 | 0.714029 | -2.20782 | 0.045687 | -3.94806 |
| ADCY8 | -1.12546 | -1.60104 | -2.75468 | 0.016296 | -3.0372 | IL20 | -1.04471 | -1.25296 | -2.20694 | 0.045762 | -3.94948 |
| ZNF578 | 0.730158 | 0.258506 | 2.754252 | 0.01631 | -3.03793 | H2AB2 | 0.421884 | -0.7132 | 2.206524 | 0.045797 | -3.95014 |
| ICAM3 | -0.8731 | 2.968504 | -2.75406 | 0.016316 | -3.03826 | NR3C1 | 0.586983 | 3.886123 | 2.206342 | 0.045812 | -3.95043 |
| EZH2 | 1.049011 | 1.792575 | 2.751862 | 0.016385 | -3.04203 | CEP85 | 0.911965 | 0.920508 | 2.206043 | 0.045837 | -3.95091 |
| ARMCX1 | -1.223 | 2.659956 | -2.75169 | 0.01639 | -3.04233 | MS4A15 | 0.683377 | -2.06832 | 2.206038 | 0.045838 | -3.95092 |
| CALHM4 | 0.567305 | -2.41599 | 2.750927 | 0.016414 | -3.04363 | TTTY18 | 0.58283 | -1.41999 | 2.205304 | 0.0459 | -3.95209 |
| HR | -0.96119 | 1.462109 | -2.74949 | 0.016459 | -3.0461 | ATP13A1 | 0.532316 | 3.144016 | 2.205066 | 0.04592 | -3.95247 |
| AACSP1 | 1.047978 | -1.88372 | 2.749333 | 0.016464 | -3.04636 | CARMIL1 | 0.66415 | -1.35988 | 2.204881 | 0.045936 | -3.95277 |
| GPHN | 0.869354 | 1.069891 | 2.748026 | 0.016505 | -3.0486 | TUB | -0.47771 | 1.383438 | -2.20472 | 0.045949 | -3.95302 |
| OR2K2 | 0.634268 | -2.36831 | 2.747704 | 0.016516 | -3.04915 | NFKBIE | -0.46534 | 3.346291 | -2.20472 | 0.04595 | -3.95303 |
| id | logFC | AveExpr | t | P.Value | B | id | logFC | AveExpr | t | P.Value | B |
| PURG | -1.1049 | -1.16501 | -2.74565 | 0.016581 | -3.05267 | FAM155A | -1.34855 | 2.812097 | -2.2042 | 0.045994 | -3.95386 |
| GTSE1-DT | 0.667955 | 0.249682 | 2.745641 | 0.016581 | -3.05269 | TOP3B | 0.523104 | 1.66157 | 2.204168 | 0.045996 | -3.95391 |
| TUBA8 | -1.01902 | 2.176743 | -2.74519 | 0.016595 | -3.05346 | NCOA5 | 0.364236 | 0.173664 | 2.204092 | 0.046002 | -3.95403 |
| RBP7 | -3.00079 | 2.123956 | -2.74455 | 0.016616 | -3.05456 | KDR | -1.9486 | 0.027147 | -2.20387 | 0.046022 | -3.95439 |
| MEX3B | 1.024542 | 1.475148 | 2.744381 | 0.016621 | -3.05485 | MTX1 | 0.445722 | 3.768323 | 2.203618 | 0.046043 | -3.95479 |
| ABCB6 | 1.122797 | 2.044289 | 2.744362 | 0.016622 | -3.05488 | GMPR | 0.803043 | 1.96599 | 2.203086 | 0.046088 | -3.95564 |
| CDK18 | -0.77663 | 0.066527 | -2.74377 | 0.016641 | -3.0559 | RHBDD3 | 0.29821 | 4.097939 | 2.202929 | 0.046101 | -3.95589 |
| MZT2B | -0.3958 | 6.97709 | -2.74341 | 0.016652 | -3.05651 | RBMY1B | 0.42001 | -1.70208 | 2.202266 | 0.046157 | -3.95695 |
| TMEM26 | -1.49312 | -0.97463 | -2.7433 | 0.016655 | -3.0567 | FAM138E | -0.65517 | -2.1226 | -2.202 | 0.04618 | -3.95737 |
| ZNF799 | 0.523177 | -0.61991 | 2.743299 | 0.016656 | -3.0567 | ZNF684 | 0.470601 | 0.048642 | 2.201908 | 0.046188 | -3.95752 |
| NOTO | -1.45348 | -0.90409 | -2.74268 | 0.016675 | -3.05776 | ARHGEF16 | -0.92567 | 1.736225 | -2.20166 | 0.046209 | -3.95792 |
| AJUBA | 0.796002 | 2.291143 | 2.742671 | 0.016676 | -3.05778 | SMC2 | 0.552595 | 1.173035 | 2.201639 | 0.046211 | -3.95795 |
| LINC00847 | -0.94181 | 1.411521 | -2.74159 | 0.01671 | -3.05963 | ITLN1 | 0.812103 | -1.82265 | 2.200998 | 0.046265 | -3.95898 |
| PANK1 | 0.924765 | 1.333998 | 2.741221 | 0.016722 | -3.06026 | LINC00685 | 0.71139 | 1.376091 | 2.200846 | 0.046278 | -3.95922 |
| SCNN1A | -1.50782 | -1.3781 | -2.74065 | 0.01674 | -3.06125 | GABRA4 | 0.730146 | -2.28181 | 2.200739 | 0.046287 | -3.95939 |
| TNFRSF21 | -0.79567 | 4.633697 | -2.73995 | 0.016763 | -3.06245 | CDC23 | 0.332766 | 2.860749 | 2.200549 | 0.046303 | -3.95969 |
| SH2D3A | -2.60363 | 0.210292 | -2.73823 | 0.016818 | -3.06539 | VIPR2 | -1.55879 | 1.224312 | -2.20042 | 0.046314 | -3.9599 |
| RBPMS2 | -0.81675 | -0.02073 | -2.73766 | 0.016837 | -3.06637 | FCMR | -0.97704 | 0.456039 | -2.20024 | 0.04633 | -3.96019 |
| IQCB1 | 0.554209 | 0.896251 | 2.737381 | 0.016845 | -3.06684 | TNFSF4 | 1.450289 | 0.214446 | 2.2002 | 0.046333 | -3.96025 |
| GJB2 | -2.04866 | 0.482755 | -2.73664 | 0.016869 | -3.06812 | GBGT1 | -0.6684 | 1.328497 | -2.19992 | 0.046357 | -3.9607 |
| UBE2Q1 | 0.751627 | 1.96515 | 2.736062 | 0.016888 | -3.0691 | GPR45 | 0.941108 | -1.25628 | 2.199742 | 0.046372 | -3.96098 |
| CHRDL2 | -2.00761 | 1.820589 | -2.73462 | 0.016935 | -3.07158 | GZMM | -0.75824 | 2.572019 | -2.19767 | 0.046549 | -3.96429 |
| ZNF556 | -1.22696 | -1.45593 | -2.73455 | 0.016937 | -3.07169 | LGALS9C | -1.27435 | 4.115515 | -2.19755 | 0.04656 | -3.96448 |
| PTGDS | -2.98523 | 4.932988 | -2.73425 | 0.016947 | -3.07221 | ULBP2 | 1.228342 | 1.27503 | 2.19702 | 0.046605 | -3.96533 |
| LAMB1 | 0.820138 | 5.64035 | 2.734027 | 0.016954 | -3.07259 | RAD51AP1 | 1.196413 | -0.92091 | 2.196445 | 0.046654 | -3.96625 |
| id | logFC | AveExpr | t | P.Value | B | id | logFC | AveExpr | t | P.Value | B |
| PPP1CB | -0.44753 | 3.170877 | -2.73364 | 0.016967 | -3.07326 | HMGCS1 | 0.647068 | 1.817037 | 2.196211 | 0.046674 | -3.96662 |
| AWAT1 | 0.797356 | -2.18642 | 2.73361 | 0.016968 | -3.0733 | SRR | 0.491575 | 0.824212 | 2.195997 | 0.046693 | -3.96696 |
| CDH8 | 1.553111 | -1.61712 | 2.733147 | 0.016983 | -3.07409 | SLC25A45 | 0.523119 | 0.169659 | 2.19576 | 0.046713 | -3.96734 |
| ZNF582-AS1 | 0.648569 | 1.939249 | 2.731207 | 0.017046 | -3.07741 | SLC27A2 | -1.55325 | -0.03651 | -2.19527 | 0.046755 | -3.96812 |
| ABCG1 | -1.3 | 2.097839 | -2.72998 | 0.017086 | -3.07951 | ATP8B2 | 0.545266 | 1.308009 | 2.194107 | 0.046856 | -3.96998 |
| GLMP | 0.585526 | 2.700851 | 2.729854 | 0.01709 | -3.07973 | CCL11 | 0.561391 | -1.96809 | 2.193642 | 0.046896 | -3.97072 |
| TONSL | 1.018985 | 1.054429 | 2.729623 | 0.017098 | -3.08013 | OR4D9 | 0.451087 | -2.15849 | 2.193506 | 0.046907 | -3.97094 |
| HTR3C | -1.32124 | -1.41777 | -2.72936 | 0.017106 | -3.08057 | OR2Y1 | 0.622451 | -0.88286 | 2.193279 | 0.046927 | -3.9713 |
| HOXA7 | 1.401895 | -0.2601 | 2.728341 | 0.01714 | -3.08232 | CEP85L | 0.518685 | -0.87461 | 2.19288 | 0.046961 | -3.97194 |
| MMACHC | 0.407826 | 0.711737 | 2.728063 | 0.017149 | -3.0828 | DIXDC1 | -0.76911 | 2.398206 | -2.19239 | 0.047004 | -3.97272 |
| SPDYC | 0.507686 | 0.447701 | 2.727586 | 0.017164 | -3.08361 | GAB3 | -0.9757 | 0.245807 | -2.19212 | 0.047027 | -3.97316 |
| TP53TG5 | 0.360948 | -1.63302 | 2.727566 | 0.017165 | -3.08365 | TNFRSF4 | -1.23283 | 1.279027 | -2.19193 | 0.047043 | -3.97345 |
| TCF15 | -2.7561 | 2.077199 | -2.72742 | 0.01717 | -3.08389 | RFPL1S | -1.21608 | -0.56205 | -2.19186 | 0.04705 | -3.97357 |
| CABCOCO1 | -1.72839 | -1.23198 | -2.72615 | 0.017211 | -3.08606 | GULP1 | -0.50835 | 1.973955 | -2.19182 | 0.047053 | -3.97363 |
| ATP8A2 | -2.45711 | 0.417675 | -2.72606 | 0.017215 | -3.08623 | NR1H3 | -0.90592 | 2.90209 | -2.19125 | 0.047102 | -3.97454 |
| SYNM | 0.984543 | 1.533566 | 2.725967 | 0.017218 | -3.08638 | BORA | 0.656133 | 0.922621 | 2.190864 | 0.047136 | -3.97516 |
| SIRPG | 0.910147 | -0.4976 | 2.724534 | 0.017265 | -3.08884 | PTH2 | -1.47236 | 1.778372 | -2.18953 | 0.047252 | -3.97728 |
| INPP4B | -1.72884 | 1.755405 | -2.7227 | 0.017325 | -3.09197 | TTYH1 | -1.83965 | 3.484546 | -2.18918 | 0.047282 | -3.97784 |
| CCK | -1.87236 | -0.81404 | -2.72036 | 0.017403 | -3.09598 | CENPJ | 0.354796 | 3.060226 | 2.189049 | 0.047293 | -3.97805 |
| SCAND2P | 0.910901 | -0.39745 | 2.719721 | 0.017425 | -3.09707 | STAR | -2.25027 | 0.601289 | -2.18859 | 0.047334 | -3.97879 |
| BEST4 | -0.87385 | 1.004686 | -2.71903 | 0.017448 | -3.09825 | RNF2 | 0.648253 | -0.16231 | 2.188537 | 0.047338 | -3.97887 |
| DOT1L | 0.711982 | 1.885789 | 2.717933 | 0.017484 | -3.10013 | MSRB1 | -0.53934 | 4.021247 | -2.18817 | 0.04737 | -3.97945 |
| OR4F21 | 0.840755 | -1.76065 | 2.717079 | 0.017513 | -3.10159 | ZNF667-AS1 | -0.84897 | 1.567707 | -2.18813 | 0.047373 | -3.97952 |
| LOC389641 | 0.639925 | -2.14723 | 2.716808 | 0.017522 | -3.10205 | CENPE | 1.291747 | 1.988605 | 2.18789 | 0.047394 | -3.9799 |
| WNT3 | -1.05904 | 2.391711 | -2.71672 | 0.017525 | -3.1022 | FEV | -1.55325 | 0.037031 | -2.18788 | 0.047395 | -3.97991 |
| id | logFC | AveExpr | t | P.Value | B | id | logFC | AveExpr | t | P.Value | B |
| CFAP44 | 0.575318 | -2.48852 | 2.716269 | 0.01754 | -3.10297 | FAM20A | -1.56186 | 3.8645 | -2.18777 | 0.047405 | -3.98009 |
| SIGLEC9 | -1.15265 | -1.34149 | -2.71626 | 0.017541 | -3.10299 | TTLL1 | 0.431362 | 1.650292 | 2.186753 | 0.047493 | -3.98171 |
| OR8D2 | 0.790642 | -1.87655 | 2.715494 | 0.017566 | -3.1043 | ZNF341 | 0.316874 | 3.074209 | 2.185922 | 0.047566 | -3.98303 |
| S1PR3 | -1.11623 | 3.727704 | -2.71391 | 0.01762 | -3.10701 | TSPAN33 | -0.88293 | 1.563297 | -2.18577 | 0.047579 | -3.98328 |
| CT45A1 | 1.052521 | -1.94214 | 2.713552 | 0.017632 | -3.10762 | CAPSL | 0.664232 | -0.74753 | 2.184743 | 0.047669 | -3.98491 |
| L3MBTL4 | -0.65071 | -2.07106 | -2.71244 | 0.017669 | -3.10951 | SDF4 | 0.606805 | 2.57135 | 2.184619 | 0.04768 | -3.98511 |
| MAGEL2 | -1.25837 | 1.883463 | -2.71233 | 0.017673 | -3.10972 | TUBB1 | 0.605607 | -1.93965 | 2.184497 | 0.047691 | -3.98531 |
| NECTIN1 | 1.036949 | 0.390867 | 2.709558 | 0.017767 | -3.11445 | DLD | 0.32587 | 0.855104 | 2.18382 | 0.04775 | -3.98638 |
| LOC100129148 | -1.93543 | -0.98181 | -2.70877 | 0.017794 | -3.1158 | KIF26B | 0.99703 | 2.904366 | 2.183806 | 0.047751 | -3.98641 |
| RASGRP2 | -1.79159 | 3.24172 | -2.70871 | 0.017796 | -3.1159 | H1-0 | -0.70571 | 3.549289 | -2.18312 | 0.047812 | -3.9875 |
| PDIA6 | 0.491295 | 4.654655 | 2.706665 | 0.017865 | -3.11939 | BDNF-AS | -0.5516 | 0.054086 | -2.18229 | 0.047885 | -3.98883 |
| TMC6 | -1.96565 | 0.618234 | -2.70551 | 0.017905 | -3.12136 | AUH | -0.46742 | 2.023874 | -2.18213 | 0.047899 | -3.98908 |
| TRAIP | 0.853786 | 3.162252 | 2.705312 | 0.017912 | -3.1217 | UTRN | 0.774025 | 2.701103 | 2.182047 | 0.047906 | -3.98921 |
| EMID1 | -1.55342 | 1.706518 | -2.70358 | 0.017971 | -3.12466 | PTCD1 | 0.345602 | 3.913229 | 2.181937 | 0.047915 | -3.98938 |
| C2orf72 | -1.33809 | -0.25436 | -2.7032 | 0.017984 | -3.12532 | EDC4 | 0.353975 | 3.839808 | 2.181828 | 0.047925 | -3.98956 |
| FDPS | 0.644783 | 4.794668 | 2.703125 | 0.017987 | -3.12544 | ODF3B | -0.93791 | 2.803026 | -2.1806 | 0.048033 | -3.99151 |
| BTLA | 0.515294 | -2.24517 | 2.70305 | 0.017989 | -3.12557 | PCCB | 0.806782 | 1.763981 | 2.180268 | 0.048063 | -3.99204 |
| CEACAM8 | 0.488222 | -2.27932 | 2.702412 | 0.018011 | -3.12666 | OR51M1 | 0.419309 | -2.20933 | 2.180048 | 0.048082 | -3.99239 |
| KCNN1 | -1.76862 | 0.197986 | -2.70219 | 0.018019 | -3.12704 | TTK | 1.491707 | 0.108924 | 2.178482 | 0.04822 | -3.99488 |
| ABCC4 | 0.967237 | 0.111094 | 2.701691 | 0.018036 | -3.12789 | DGCR5 | -1.68414 | -1.06978 | -2.17822 | 0.048243 | -3.9953 |
| SFT2D2 | 0.826916 | 2.12718 | 2.701103 | 0.018056 | -3.12889 | SFTPA1 | 0.440503 | -1.31344 | 2.176822 | 0.048368 | -3.99752 |
| FMO4 | 0.89919 | 0.04321 | 2.700975 | 0.018061 | -3.12911 | PNMA5 | -0.65939 | 2.57459 | -2.17595 | 0.048445 | -3.9989 |
| ATF6 | 0.457623 | 3.210546 | 2.699966 | 0.018096 | -3.13083 | ZFP82 | 0.43114 | 0.848381 | 2.175273 | 0.048505 | -3.99999 |
| HCK | -2.25567 | 0.344785 | -2.69945 | 0.018113 | -3.13171 | CCDC151 | -1.14661 | 0.551259 | -2.17522 | 0.04851 | -4.00008 |
| id | logFC | AveExpr | t | P.Value | B | id | logFC | AveExpr | t | P.Value | B |
| GRIK2 | 1.152334 | -0.91644 | 2.698414 | 0.01815 | -3.13348 | RHOD | -1.08481 | 2.024518 | -2.17492 | 0.048537 | -4.00055 |
| EFS | -1.21706 | 3.887217 | -2.69807 | 0.018162 | -3.13408 | LOC153910 | 0.466198 | -2.24289 | 2.172812 | 0.048725 | -4.0039 |
| RTN4R | -1.44017 | 2.632803 | -2.69785 | 0.018169 | -3.13445 | PPL | -0.82272 | 1.374724 | -2.17092 | 0.048894 | -4.00691 |
| TENM3 | 1.341984 | 0.915804 | 2.695203 | 0.018261 | -3.13897 | RPL23AP32 | 0.979517 | 1.926393 | 2.170656 | 0.048918 | -4.00733 |
| C1QTNF7 | -1.73395 | -0.5631 | -2.69477 | 0.018276 | -3.1397 | GRK3 | -0.77806 | 2.602867 | -2.16972 | 0.049002 | -4.00881 |
| CCT6B | 0.629103 | -0.71939 | 2.694219 | 0.018296 | -3.14064 | TAFA4 | -1.19684 | -1.60634 | -2.16966 | 0.049008 | -4.00891 |
| IDO2 | 1.59645 | -1.86637 | 2.694015 | 0.018303 | -3.14099 | RCC2 | 0.539386 | 3.803035 | 2.169655 | 0.049008 | -4.00892 |
| HVCN1 | -1.31458 | 0.951924 | -2.69265 | 0.018351 | -3.14332 | C9orf131 | 0.509814 | -0.56701 | 2.169497 | 0.049022 | -4.00917 |
| SPTB | -1.74508 | 0.352308 | -2.69254 | 0.018354 | -3.14351 | CKAP2L | 1.150386 | 0.487072 | 2.169214 | 0.049048 | -4.00962 |
| TMEM81 | 0.732017 | -0.48369 | 2.69247 | 0.018357 | -3.14363 | ADPGK | 0.448489 | 2.164884 | 2.168392 | 0.049121 | -4.01092 |
| CA6 | 0.6916 | -0.85322 | 2.690982 | 0.018409 | -3.14617 | TNFRSF1B | -0.99165 | -0.01628 | -2.16777 | 0.049177 | -4.01191 |
| CACNA1I | -1.34298 | -0.32305 | -2.68981 | 0.01845 | -3.14817 | PRRC2A | 0.438297 | 2.684386 | 2.166306 | 0.04931 | -4.01423 |
| EN2 | 0.627832 | 3.327134 | 2.688988 | 0.01848 | -3.14957 | CXCR4 | -2.01191 | 2.903826 | -2.16614 | 0.049325 | -4.01449 |
| CPXCR1 | 1.018726 | -1.82964 | 2.688723 | 0.018489 | -3.15002 | KLHL22 | 0.495943 | 2.402287 | 2.165213 | 0.049409 | -4.01597 |
| SCRG1 | 2.391289 | 0.760943 | 2.688474 | 0.018498 | -3.15045 | XAGE3 | 0.535711 | -1.80264 | 2.164738 | 0.049452 | -4.01672 |
| MSMB | 0.672419 | -2.21421 | 2.687805 | 0.018521 | -3.15159 | MSX2 | 1.163739 | -0.92915 | 2.164013 | 0.049517 | -4.01787 |
| NUDT4 | 0.879398 | -0.65535 | 2.687752 | 0.018523 | -3.15168 | EBAG9 | -0.59586 | 2.274667 | -2.16332 | 0.04958 | -4.01896 |
| C3orf18 | -0.58475 | 3.194688 | -2.6874 | 0.018536 | -3.15228 | GPR176 | 0.652708 | 2.200711 | 2.162982 | 0.049611 | -4.01951 |
| TNNT1 | -3.03611 | 3.092686 | -2.68728 | 0.01854 | -3.15249 | POLD1 | 0.557501 | 2.651402 | 2.162389 | 0.049665 | -4.02045 |
| MMP24 | -1.5855 | 2.734959 | -2.68715 | 0.018544 | -3.15271 | INPP4A | 0.366599 | 0.469965 | 2.162208 | 0.049681 | -4.02074 |
| AP3B2 | -1.10134 | 0.304889 | -2.68623 | 0.018577 | -3.15428 | ABCC6P1 | -0.90527 | -1.87719 | -2.16187 | 0.049712 | -4.02127 |
| BMERB1 | -0.80841 | 3.910589 | -2.68475 | 0.01863 | -3.1568 | HLA-DMB | -2.47069 | -0.33912 | -2.16155 | 0.049741 | -4.02178 |
| RIMS3 | -0.64503 | 0.549825 | -2.68441 | 0.018642 | -3.15738 | SSUH2 | 0.661825 | 0.096806 | 2.161455 | 0.04975 | -4.02193 |
| NOG | 2.131179 | 0.327266 | 2.683877 | 0.018661 | -3.15829 | PSAT1 | 1.540276 | 2.884524 | 2.161384 | 0.049757 | -4.02204 |
| NIPAL2 | -1.55792 | 0.374762 | -2.68386 | 0.018661 | -3.15831 | CNKSR2 | 0.643464 | -1.4821 | 2.161175 | 0.049776 | -4.02238 |
| id | logFC | AveExpr | t | P.Value | B | id | logFC | AveExpr | t | P.Value | B |
| SERAC1 | 0.786178 | 0.02061 | 2.683096 | 0.018689 | -3.15962 | SIM1 | 0.624273 | -0.85513 | 2.16093 | 0.049798 | -4.02276 |
| ZBED5-AS1 | -0.54261 | 1.646681 | -2.68299 | 0.018693 | -3.15981 | ADAM23 | 1.20626 | 1.337451 | 2.160705 | 0.049819 | -4.02312 |
